# Switching on Cytotoxicity of Water-Soluble Diiron Organometallics by UV Irradiation

**DOI:** 10.1021/acs.inorgchem.2c00504

**Published:** 2022-05-10

**Authors:** Lorenzo Biancalana, Manja Kubeil, Silvia Schoch, Stefano Zacchini, Fabio Marchetti

**Affiliations:** † Department of Chemistry and Industrial Chemistry, University of Pisa, Via G. Moruzzi 13, 56124 Pisa, Italy; ‡ Institute of Radiopharmaceutical Cancer Research, Helmholtz-Zentrum Dresden-Rossendorf, Bautzner Landstrasse 400, 01328 Dresden, Germany; § Department of Industrial Chemistry “Toso Montanari”, University of Bologna, Viale del Risorgimento 4, 40136 Bologna, Italy

## Abstract

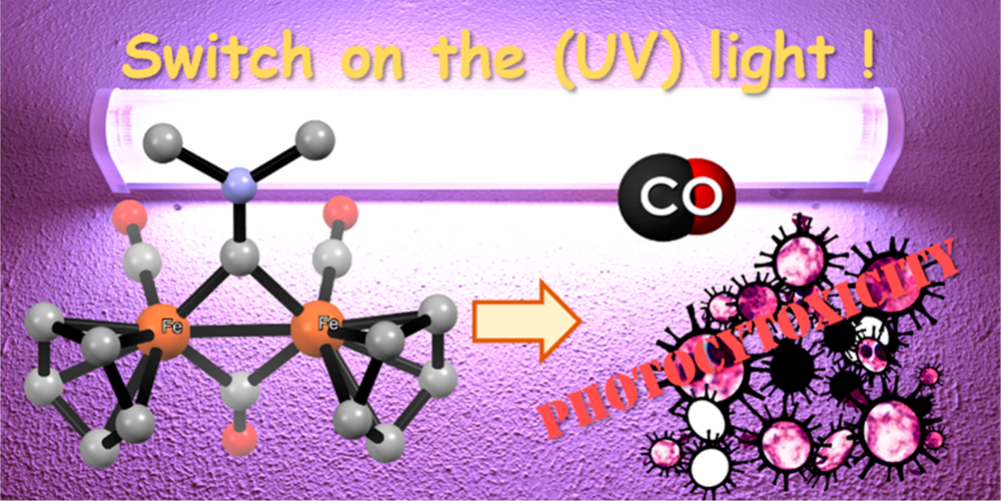

The diiron compounds [Fe_2_Cp_2_(CO)_2_(μ-CO)(μ-CSEt)]CF_3_SO_3_, [**1**]CF_3_SO_3_, K[Fe_2_Cp_2_(CO)_3_(CNCH_2_CO_2_)], K[**2**], [Fe_2_Cp_2_(CO)_2_(μ-CO)(μ-CNMe_2_)]NO_3_, [**3**]NO_3_, [Fe_2_Cp_2_(CO)_2_(PTA){μ-CNMe(Xyl)}]CF_3_SO_3_, [**4**]CF_3_SO_3_, and [Fe_2_Cp_2_(CO)(μ-CO){μ–η:^1^η^3^-C(4-C_6_H_4_CO_2_H)CHCNMe_2_}]CF_3_SO_3_, [**5**]CF_3_SO_3_, containing a bridging carbyne, isocyanoacetate, or vinyliminium ligand, were investigated for their photoinduced cytotoxicity. Specifically, the novel water-soluble compounds K[**2**], [**3**]NO_3_, and [**4**]CF_3_SO_3_ were synthesized and characterized by elemental analysis and IR and multinuclear NMR spectroscopy. Stereochemical aspects concerning [**4**]CF_3_SO_3_ were elucidated by ^1^H NOESY NMR and single-crystal X-ray diffraction. Cell proliferation studies on human skin cancer (A431) and nontumoral embryonic kidney (HEK293) cells, with and without a 10-min exposure to low-power UV light (350 nm), highlighted the performance of the aminocarbyne [**3**]NO_3_, nicknamed **NIRAC** (Nitrate-Iron-Aminocarbyne), which is substantially nontoxic in the dark but shows a marked photoinduced cytotoxicity. Spectroscopic (IR, UV–vis, NMR) measurements and the myoglobin assay indicated that the release of one carbon monoxide ligand represents the first step of the photoactivation process of **NIRAC**, followed by an extensive disassembly of the organometallic scaffold.

## Abstract 2

A diiron cyclopentadienyl aminocarbyne complex (**NIRAC**), soluble and inert in water and nontoxic in the dark, exerts antiproliferative activity on cancer cells upon photoactivation by short time low-power UV irradiation.

## 1 Introduction

The search for new effective anticancer drugs based on transition metals is at the forefront of research and the design of photoactivable metal complexes is a promising strategy to enhance selectivity and reduce side effects.^1^ Ideally, a nontoxic prodrug is injected and then activated by localized irradiation, resulting in the formation of reactive species finally leading to cell death.

Besides, the photoinduced release of carbon monoxide has been intensively studied in the last two decades due to the therapeutic effects provided by this small molecule in low concentrations,^2^ and many different metal-based photoactivable CO-releasing molecules (photoCORM) have been studied in biological settings.^3,4^ Interestingly, some Mn(I), Re(I), and Ru(II) complexes manifested photoinduced antiproliferative activity on cancer cells or antimicrobial activity upon exposure to UV (305–365 nm)^5,6^ or visible (>440 nm)^7^ light for a relatively short time (typically 10–15 or 30–45 min, respectively). These biological effects have been often associated with the intracellular release of CO, although the role of the metal fragment deserves consideration.^6,8^

**Scheme 1:**
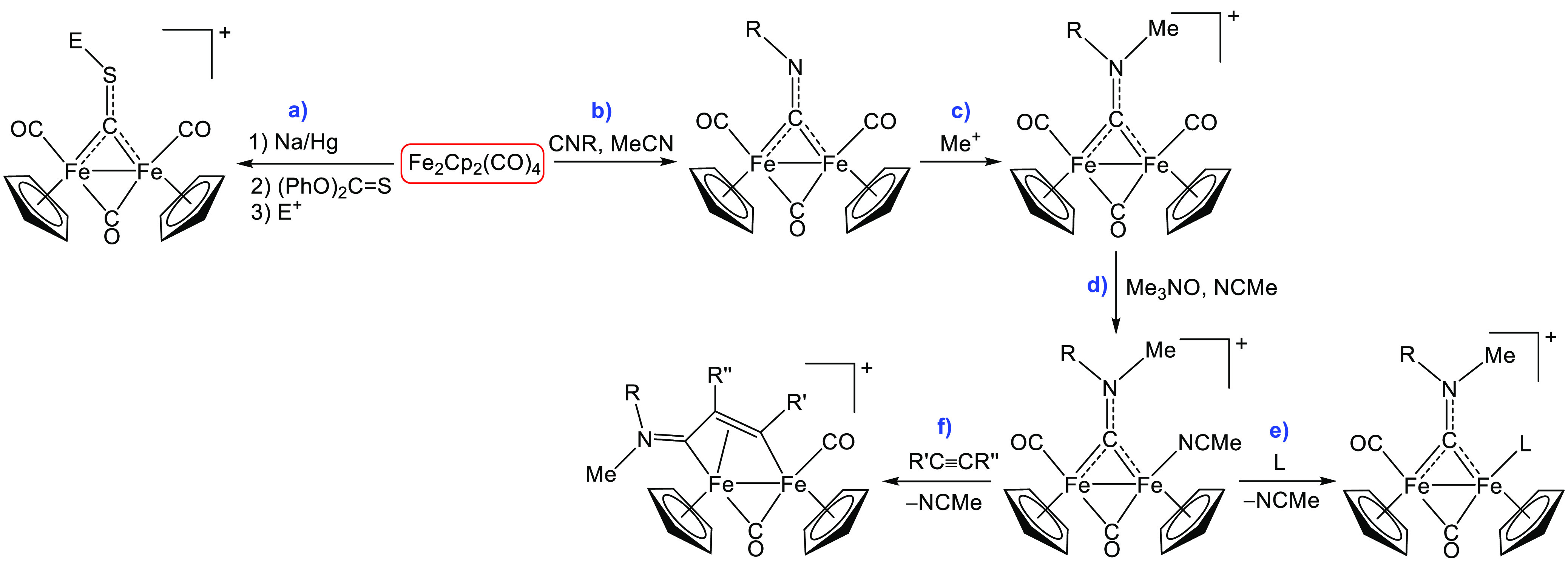
Synthesis of Diiron Carbonyl Complexes with a *Bridging Hydrocarbyl Ligand* from [Fe_2_Cp_2_(CO)_4_] **a)***Thiocarbyne* complexes via preliminary reduction to [FeCp(CO)_2_]^−^ (E = Me, Et; CF_3_SO_3_^–^ or BF_4_^–^ salts);^11^**b)***isocyanide* complexes (R = alkyl or aryl);^12^**c)***aminocarbyne* complexes (most frequently CF_3_SO_3_^–^ and also Cl^–^, BF_4_^–^, and NO_3_^–^ salts);^13^**d)** aminocarbyne complexes with a labile acetonitrile ligand;^14^**e)** aminocarbyne derivatives via acetonitrile substitution (L = phosphane, isocyanide, amine, imine, DMSO);^15^**f)***vinyliminium* complexes via alkyne insertion into the Fe-carbyne bond (R′ = alkyl, aryl, CO_2_Me, SiMe_3_, pyridyl, thiophenyl; R′′ = H, Me, Et, Ph, CO_2_Me; CF_3_SO_3_^–^ salts).^16^

Iron is an appealing candidate to design metal-based drugs, due to its biocompatibility, versatility, and low toxicity in many forms.^9^ Indeed, a number of monoiron(II) cyclopentadienyl and thiolate-bridging diiron(I) complexes have been investigated as CORMS or photoCORMs,^3d^ while ferrocene derivatives recently emerged as promising anticancer and/or antimalarial agents.^10^ In this respect, the commercial [Fe_2_Cp_2_(CO)_4_] can be employed as a starting material to access robust cationic dinuclear derivatives by means of straightforward synthetic pathways. In more detail, thiocarbyne complexes are available from [Fe_2_Cp_2_(CO)_4_] by a reductive route which has been known since the 1970s (Scheme 1, path a).^11^ Differently, thermal CO/CNR substitution affords neutral monoisocyanide derivatives as mixtures of terminal- and bridging-CNR isomers (path b);^12^ subsequent nitrogen alkylation gives an aminocarbyne ligand which firmly occupies a bridging position (path c).^13^ Further CO removal, using the TMNO (trimethylamine-*N*-oxide) strategy, results in the formation of relatively stable acetonitrile complexes (path d)^14^ which, however, undergo MeCN substitution by a variety of monodentate ligands to yield cationic aminocarbyne derivatives (path e).^15^ Differently, in the presence of alkynes, acetonitrile displacement is followed by fast alkyne insertion into the iron–carbyne bond, according to a quite general reaction: note that over 100 diiron vinyliminium complexes with the general structure provided by path f) in Scheme 1 have been reported to date.^16^

Aminocarbyne^17^ and vinyliminium^18^ complexes based on the [Fe_2_Cp_2_(CO)_*x*_] (*x* = 2, 3) scaffold have been the subject of recent studies by some of us to assess their potential as anticancer agents. These compounds, aside from the metal element, show other ideal prerequisites for a pharmacological application such as simple and high-yielding preparation up to gram scale, appreciable solubility in water, amphiphilicity, and inertness in aqueous solution. Some aminocarbyne and vinyliminium complexes revealed highly cytotoxic *in vitro* on various cancer cell lines, including 3D models, with a considerable degree of selectivity with respect to nontumoral cells. In general, the biological activity for each category of compounds is highly affected by the nature of the substituents on the bridging ligand, and quite often the most hydrophilic compounds are poorly cytotoxic. All diiron complexes are able to release carbon monoxide in an aqueous solution, albeit very slowly and only as a result of a prolonged thermal treatment (37 °C, 72 h; GC detected).^17b,18c^ Such a process is likely initiated by a thermally induced CO release step, and in the absence of suitable ligands in solution (*e.g*., DMSO), the decarbonylated organometallic scaffold undergoes fast disassembly and aerobic oxidation, with concomitant formation of iron(III) oxides.^17,18a^ Notably, a significant increase of intracellular CO levels and reactive organic species (ROS) was detected upon treatment with the two most performing aminocarbyne complexes, suggesting that their biological activity benefits from the combined effects provided by the metallic frame and the released carbon monoxide.^2d,17b^

In light of these premises, we were interested in investigating whether diiron cyclopentadienyl complexes could be activated by irradiation, particularly those hydrophilic complexes known (or expected) to be scarcely cytotoxic otherwise. Herein we selected five complexes, one for each category illustrated in Scheme 1, including three novel compounds, for a preliminary investigation of their potential as photoactivable anticancer drugs.

## 2 Results and Discussion

### 2.1 Synthesis and Characterization

**Figure 1:**
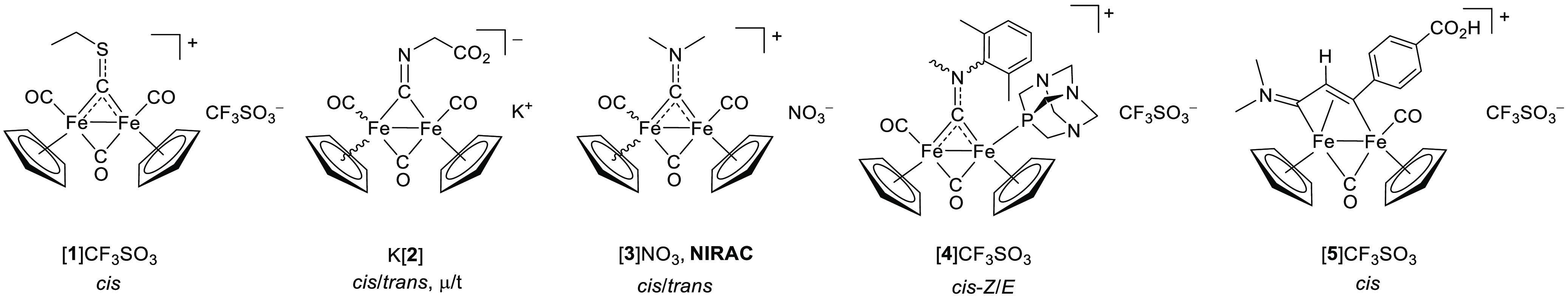
Selected water-soluble diiron bis-cyclopentadienyl carbonyl compounds investigated in this work, bearing a thiocarbyne ([**1**]CF_3_SO_3_), isocyanide (K[**2**]; terminal isocyanide form not shown), aminocarbyne ([**3**]NO_3_, [**4**]CF_3_SO_3_), or vinyliminium ([**5**]CF_3_SO_3_) bridging ligand. Stereochemical details are specified for each compound.

The {Fe_2_Cp_2_} complexes selected for the present investigation are reported in Figure 1. The preparation of the thiocarbyne compound [**1**]CF_3_SO_3_ from [Fe_2_Cp_2_(CO)_2_(μ-CO)(μ-CS)] and ethyl triflate is described here for the first time, although other salts containing the same cation **1**^**+**^ were reported.^11a,11b^ The vinyliminium compound [**5**]CF_3_SO_3_^18a^ was prepared according to the literature. In these compounds, the net positive charge associated with the organometallic scaffold combined with a suitable counteranion (CF_3_SO_3_^–^) supplies an appreciable water solubility (*see below*). By contrast, neutral isocyanide compounds [Fe_2_Cp_2_(CO)_3_(CNR)] are generally insoluble in water. In this respect, potassium isocyanoacetate, easily obtained from the commercial ethyl ester (see the Experimental Section), combines the isocyanide function with a water-solubilizing group (carboxylate). Therefore, K[**2**] was synthesized in one step from [Fe_2_Cp_2_(CO)_4_] via CO/isocyanide exchange (Scheme 1b) in refluxing MeCN and isolated as a red-purple solid in 62% yield. Complex **2**^**–**^ was previously obtained as the [K(18-crown-6)]^+^ salt from a more elaborated route, involving the preliminary photolytic generation of [Fe_2_Cp_2_(CO)_3_(NCMe)].^19^

Along the same line of thought, we decided to replace the typical CF_3_SO_3_^–^ anion in the aminocarbyne complex **3**^+^ with the more hydrophilic NO_3_^–^. Note that, recently, we proposed the association of the nitrate anion to cationic ruthenium arene complexes resulting in a considerable improvement of the water solubility.^20^ Therefore, methylation of [Fe_2_Cp_2_(CO)_3_(CNMe)] was carried out with excess CH_3_I in CH_2_Cl_2_, followed by alumina chromatography and a final ion metathesis step with AgNO_3_ in MeOH. The novel compound [**3**]NO_3_, nicknamed **NIRAC**, was isolated as a dark red solid in 70% yield.

Similarly, the phosphane PTA (1,3,5-triaza-7-phosphaadamantane) is a widely employed ligand to enhance the aqueous solubility of metal complexes.^17,21^ In this regard, the novel compound [**4**]CF_3_SO_3_ was prepared from the tris-carbonyl precursor [Fe_2_Cp_2_(CO)_2_(μ-CO){μ-CNMe(Xyl)}]CF_3_SO_3_ via a classical two-step TMNO procedure (Scheme 1d+e); MeCN/phosphane exchange took place in refluxing THF, and the resulting dark green-brown solid was recovered by filtration in 87% yield. Compound [**4**]CF_3_SO_3_ represents one of the few diiron derivatives containing the PTA ligand.^17a,22^

Complexes [**1**]CF_3_SO_3_, K[**2**], [**3**]NO_3_, and [**4**]CF_3_SO_3_ were characterized by elemental analysis and IR and multinuclear NMR spectroscopy; spectra are given in Figures S1–S15 in the Supporting Information.

In [**1**]CF_3_SO_3_, the infrared carbonyl bands were detected at 2026, 2003, and 1837 cm^–1^ (CH_2_Cl_2_ solution), similar to those reported for the homologous tetrafluoroborate salt.^11b^ NMR spectra (acetone-*d*_6_ solution) comprise a unique set of signals, indicating the *cis* arrangement adopted by the Cp ligands; inequivalence of the Cp, CO, and CH_2_ groups is suggestive of hindered rotation around the μ–C–S bond.^11a^ The diagnostic ^13^C NMR resonance accounting for the carbyne falls at 406 ppm.

The IR spectra of K[**2**] (in the solid state and in the MeOH solution) are indicative of four isomers. Two bands of comparable intensity, ascribable to μ–C–N stretching (*e.g*., 2140 and 1719 cm^–1^ in the solid state), are related to isomers containing a terminal or bridging isocyanide ligand (μ/t in Figure 1). On the other hand, two sets of carbonyl bands were recognized in each spectrum, suggesting the occurrence of *cis*/*trans* isomerism (with reference to the mutual geometry of the Cp’s). In analogous [Fe_2_Cp_2_(CO)_3_(CNR)] complexes, *cis* and *trans* isomers were usually observed, interconverting into each other in solution via the Adams-Cotton mechanism.^12,23^ In the solid-state IR spectrum of K[**2**], the higher frequency CO absorptions (1979, 1778) are less intense then those occurring at lower frequencies (1933, 1737), suggesting the prevalence of the *trans* isomers. The reverse situation was recognized in the methanol solution, pointing out a prevalence of *cis* species, in agreement with the relatively high polarity of the medium.^12^ The ^1^H and ^13^C NMR spectra of K[**2**] in CD_3_OD and DMSO-*d*_6_ display broadened resonances, in alignment with the different isomers undergoing a fluxional process on the NMR time scale.

The IR and ^1^H/^13^C NMR features of [**3**]NO_3_ in the MeCN or D_2_O solution, respectively, resemble those of the related triflate salt.^13^ The dominant *cis* isomer is accompanied by a minor amount (*ca*. 10%) of the *trans* isomer.^24^ The nitrate anion manifests itself with an intense and broad absorption at 1338 cm^–1^ in the solid-state IR spectrum and a diagnostic ^14^N NMR resonance at −5 ppm in D_2_O. Interestingly, the carbonyl and N–C(carbyne) stretching wavenumbers of **3**^+^ in the solid state are significantly affected by the anion (2021, 1991, 1814, 1592 *vs*. 1992, 1972, 1825, 1607 cm^–1^ for CF_3_SO_3_^–^ and NO_3_^–^ salts, respectively; Figure S3).

**Scheme 2:**
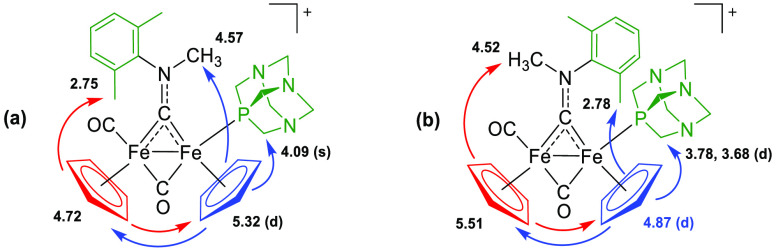
Structures of *cis*-*E* (**a**) and *cis*-*Z* (**b**) Diastereomers of **4**^**+**^ Substituents/groups with the highest priority according to CIP rules are highlighted in green. NOE effects observed upon irradiation of the cyclopentadienyl bonded to the {Fe(CO)} (red) or the {Fe(PTA)} unit (blue) are represented by the arrows. Selected ^1^H NMR chemical shifts (ppm) are reported next to each group (d for doublet).

In the solid-state IR spectrum of [**4**]CF_3_SO_3_, intense bands at 1992 and 1810 cm^–1^ are due to the terminal and bridging carbonyls, respectively, and a medium intensity band at 1506 cm^–1^ has been attributed to the N–C(carbyne) stretching. The NMR spectra of [**4**]CF_3_SO_3_ in acetone-*d*_6_ contain two sets of resonances, corresponding to *cis*-*Z* and *cis*-*E* diastereomers (10:1 ratio, by ^1^H NMR), differing in the relative orientation of the *N*-substituents with respect to the μ–C–N bond, which holds some double bond character. For instance, two signals are present in the ^31^P spectrum, related to the PTA ligand (*Z*: −18.9 ppm, *E*: −22.8 ppm). The stereochemistry was unequivocally assigned by ^1^H NOESY experiments, upon irradiation of each cyclopentadienyl (Figures S14 and S15), taking advantage of the spin–spin coupling to identify the Cp adjacent to the phosphorus atom (labeled Cp^P^, ^3^*J*_HP_ ≈ 1.5 Hz); the results are summarized in Scheme 2. Briefly, NOE effects between Cp ligands were observed for both isomers, confirming their localization on the same side of the Fe_2_(μ-C)_2_ unit (*cis* stereochemistry), while other NOE peaks indicated their proximity to *N*-CH_3_, *N*-xylyl, or PCH_2_ groups. Thus, the *cis*-*Z* isomer, featuring the Xyl substituent on the aminocarbyne ligand pointing toward the PTA ligand, is the major isomer in the solution. Interestingly, in the *E* isomer, the Cp next to the carbonyl ligand resonates at a lower chemical shift with respect to Cp^P^ (4.72 and 5.32 ppm, respectively), while the opposite is observed for the *Z* counterpart (5.51, 4.87 ppm).

**Figure 2:**
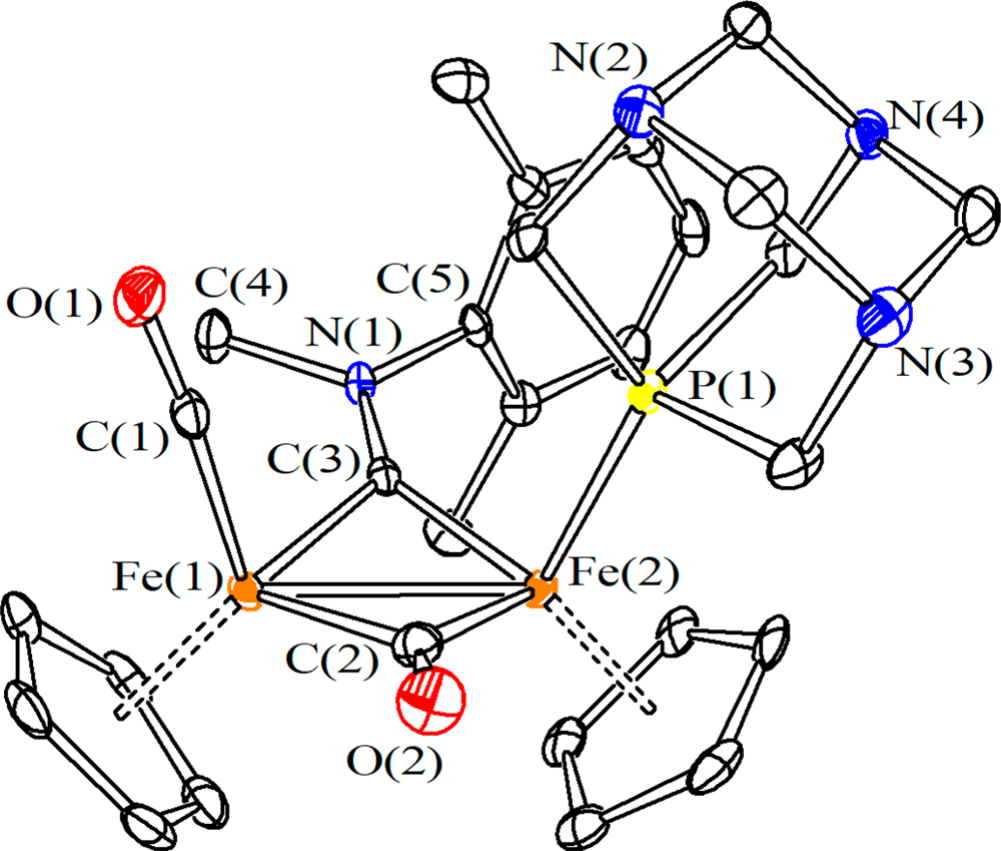
View of the structure of the cation within *cis*-*Z*-[**4**]CF_3_SO_3_·0.5CH_3_CN. Displacement ellipsoids are at the 50% probability level. Hydrogen atoms have been omitted for clarity. Main bond distances (Å) and angles (deg): Fe(1)–Fe(2) 2.4985(5), Fe(1)–C(1) 1.765(3), Fe(1)–C(2) 1.957(3), Fe(2)–C(2) 1.899(3), Fe(1)–C(3) 1.885(3), Fe(2)–C(3) 1.855(3), Fe(2)–P(1) 2.1974(7), C(1)–O(1) 1.133(3), C(2)–O(2) 1.169(3), C(3)–N(1) 1.308(3), Fe(1)–C(1)–O(1) 174.7(2), Fe(1)–C(2)–Fe(2) 80.74(11), Fe(1)–C(3)–Fe(2) 83.83(11), Fe(1)–C(3)–N(1) 132.24(19), Fe(2)–C(3)–N(1) 143.7(2), C(3)–N(1)–C(4) 123.7(2), C(3)–N(1)–C(5) 122.6(2), C(4)–N(1)–C(5) 113.7(2).

The structure of [**4**]CF_3_SO_3_·0.5CH_3_CN was ascertained by a single-crystal X-ray diffraction study (Figure 2). The structure of the cation **4**^**+**^ closely resembles that previously reported for [Fe_2_Cp_2_(CO)(PTA)(μ-CO)(μ-CNMe_2_)]^+^, being the main bonding parameters are very similar.^17a^ The organometallic cation shows the *Z* stereochemistry, as also found in the crystal structure of the analogous diphenylphosphane derivative [Fe_2_Cp_2_(CO)_2_(PPh_2_H){μ-CNMe(Xyl)}]CF_3_SO_3_.^15b^ It should be remarked that, by contrast, the xylyl group and various N-, S-, or C-donor ligands (L) are usually located on opposite directions (*E* stereochemistry) in the solid-state structures of [Fe_2_Cp_2_(CO)_2_(L){μ-CNMe(Xyl)}]^+^ complexes.^25^

Aqueous solubility (21 °C) of [**1**]CF_3_SO_3_, [**4**]CF_3_SO_3_, and [**5**]CF_3_SO_3_ ranges from 1 to 6 mmol·L^–1^; these values are suited for biological applications (*cf*. the reported water solubility of the anticancer drug cisplatin is 8.4 mmol·L^–1^).^26^ Instead, K[**2**] (>0.5 mol·L^–1^) and [**3**]NO_3_ (≥0.11 mol·L^–1^; *ca*. 3.4-fold increase with respect to the CF_3_SO_3_^–^ salt^17a^) are much more soluble, confirming the beneficial role played by K^+^ and NO_3_^–^ as counterions in this respect.

The cationic complexes remain intact in water, with the ^1^H NMR spectra in D_2_O showing only resonances due to the starting material (Figures S18–S22). Two sets of signals of comparable intensity are present in the ^13^C NMR spectrum of a concentrated solution of K[**2**] (Figure S23), representing terminal/bridging coordination isomers. For instance, the isocyanide carbon and the methylene group give rise to resonances at 160/48 ppm for [Fe_2_Cp_2_(μ-CO)_2_(CO)(CNCH_2_CO_2_)]^−^ and 267/65 ppm for [Fe_2_Cp_2_(CO)_2_(μ-CO)(μ-CNCH_2_CO_2_)]^−^, respectively, as expected for a terminal and a bridging isocyanide, respectively.^12^ Solutions of the diiron compounds in water show medium-intensity absorptions (ε ≈ 3–5·10^3^ M^–1^·cm^–1^) in the UV region (308–340 nm); bands in the visible range extend up to 600 nm but are rather weak (Figure S24).

### 2.2 Spectroscopic Studies under UV Irradiation

**Figure 3:**
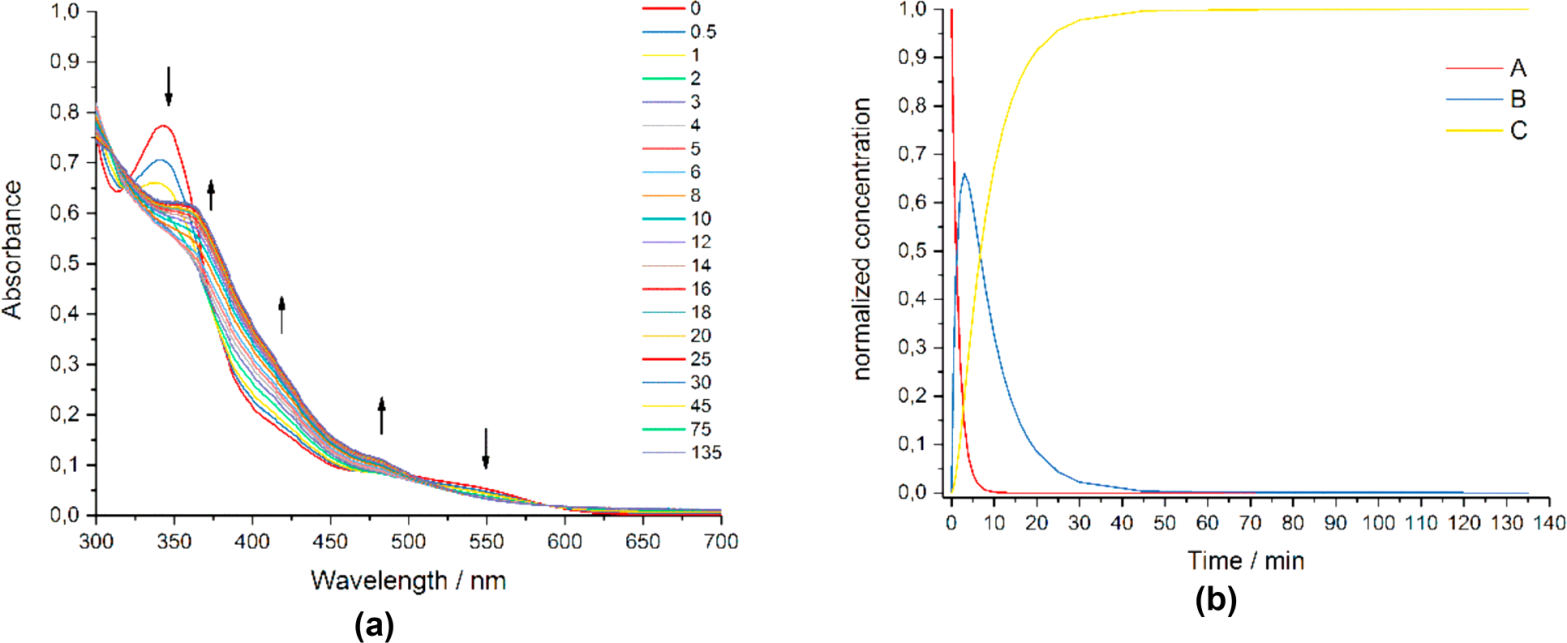
(a) UV/vis spectra recorded on a solution of [**3**]NO_3_, **NIRAC** (150 μM) in H_2_O after different times of exposure to 350 nm radiation (*E*_v_ ∼ 6 mW/cm^2^) at 25 °C. (b) Concentration profiles for **3**^+^ (A) and the associated photoproducts (B and C), derived by fitting the experimental data using MCR-ALS analysis. *k*_1_ = 0.65 ± 0.06 min^–1^ and *k*_2_ = 0.135 ± 0.008 min^–1^.

The behavior of the complexes upon irradiation was investigated, in order to identify possible photoactivation processes. First, a solution of [**3**]NO_3_ in water was exposed to 350 nm light (up to 135 min) and monitored by UV–vis spectroscopy. Under such conditions, the absorptions at 342 and 533 nm were progressively replaced by new bands at 360, 420, and 470 nm (Figure 3a). These changes are appreciable even after a very short irradiation time (<1 min). By comparison, no variation of the UV–vis spectrum was observed when the same solution was kept in the dark for at least one month, demonstrating the marked inertness of the complex in an aqueous solution at room temperature.

UV–vis data were treated with the Multivariate Curve Resolution-Alternating Least Squares (MCR-ALS) method, and two kinetic processes were identified (Figure 3b). The determined values of *k*_n_ are considered conditional rate constants and constitute a good evaluation of the quantum yield under comparable measurement conditions and similar extinction coefficients (see the Experimental Section).^5d^

On the other hand, only a progressive decrease of intensity of the ^13^C NMR resonances belonging to **3**^+^ was observed for a D_2_O solution of [**3**]NO_3_ exposed to 350 nm radiation, even after 4 h (Figure S25).

The absence of new NMR signals is not surprising, if compared to the aerobic oxidative process ascertained in an aqueous solution at 37 °C (see the Introduction). In fact, no cyclopentadienyl complex other than the starting material was ever detected in a solution by NMR and no organic material was present in the final iron-containing precipitate, judging by IR and RAMAN analyses.^17,18^ In this regard, the release of cyclopentadiene (CpH) from [FeCpX(CO)_2_] (X = Cl, Br, I) in an aqueous solution by blue-light irradiation was reported.^27^

**Figure 4:**
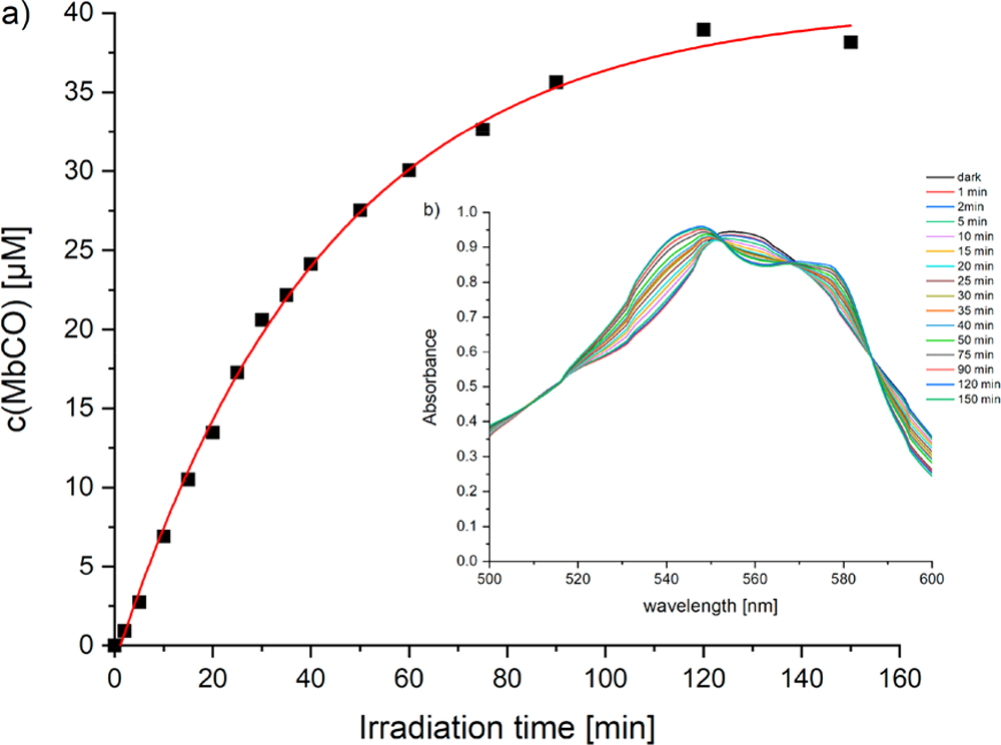
Time-dependent formation of carbonmonoxy-myoglobin (MbCO) upon excitation at 350 nm (*E*_v_ = 6 mW/cm^2^, 25 °C) of a solution of [**3**]NO_3_, **NIRAC** (17 μM), sodium dithionite (8 mM), and myoglobin (Mb, 68 μM) in 10 mM PBS buffer under nitrogen atmosphere. The inset shows the corresponding UV/vis spectral changes in the Q-band region, representative of Mb to MbCO conversion.

Since the above-mentioned thermal decomposition pathway of aminocarbyne complexes entails their complete decarbonylation (see the Introduction), we investigated the release of carbon monoxide from [**3**]NO_3_ promoted by UV irradiation. The myoglobin assay monitors spectroscopically the conversion of deoxy-myoglobin (Mb) to carbonmonoxy-myoglobin (MbCO).^28^ A fresh solution of [**3**]NO_3_ and reduced myoglobin was incubated in the dark for 15 min and then exposed to 350 nm light (*E*_v_ = 6 mW/cm^2^) at 25 °C while being monitored by UV–vis spectroscopy. A 4-fold excess of Mb to [**3**]NO_3_ was chosen, to ensure one protein for each carbonyl that can potentially be released from the metal coordination sphere. New bands at 540 and 577 nm, diagnostic of MbCO, grew with increasing irradiation time, while the intensity of the Q-band at 557 nm of Mb decreased (Figure 4). The formation of Mb into MbCO was followed until no more spectral changes in the Q-band were observed (150 min). At this point, about 38 μM MbCO was formed, corresponding to *ca*. 2.2 equivalents of CO released from [**3**]NO_3_.

In another experiment, a solution of [**3**]NO_3_ in water spiked with DMSO (0.8% *v*/*v*) was exposed to 350 nm radiation (*E*_v_ ≈ 6 mW/cm^2^) at 37 °C for a variable time (0–60 min), followed by lyophilization and analysis of the residue via IR (ATR) spectroscopy. The carbonyl stretching bands of **3**^+^ (1990, 1971, and 1823 cm^–1^) progressively decreased with irradiation time, along with the appearance of new bands around 1985 and 1799 cm^–1^ (Figure S26). These changes are consistent with the formation of [Fe_2_Cp_2_(CO)(DMSO)(μ-CO)(μ-CNMe_2_)]^+^, **6**^**+**^, upon CO/DMSO replacement, and are appreciable after 10-min irradiation.^29^ In this respect, complex **6**^**+**^ was prepared in one step from [**3**]CF_3_SO_3_, Me_3_NO·2H_2_O, and DMSO in refluxing THF, purified by alumina chromatography and isolated as [**6**]CF_3_SO_3_ in 75% yield (see the Experimental Section and Figures S5, S16, and S17).

**Scheme 3:**
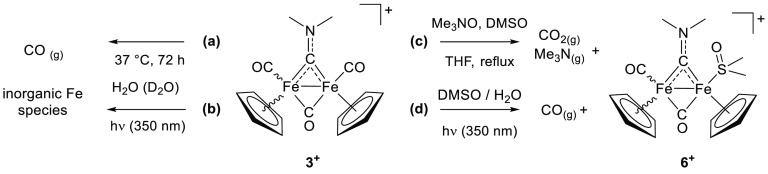
Activation Processes of the Aminocarbyne **3**^+^ with Reference to Identified Products Only Thermal (**a**) and photolytic (**b**) disassembly in an aqueous solution and thermochemical (**c**) and photolytic (**d**) CO/DMSO replacement in a water/DMSO solution.

Despite the collected data being far from exhaustive, a parallel could be traced between the thermal and photoactivation of the tricarbonyl aminocarbyne **3**^**+**^ (Scheme 3). Electronic excitation in water triggers a rapid, presumably complete disruption of the diiron framework, with a huge acceleration with respect to thermal effects alone (*cf*. 86% of intact [**3**]NO_3_ in a solution after 72 h at 37 °C; see the Experimental Section). Conversely, in the presence of DMSO, the reactive species formed by photoinduced cleavage of one carbonyl ligand can, in part, be trapped (stabilized) by sulfoxide coordination.

Likewise, changes in the solid-state IR spectra of the tricarbonyl thiocarbyne [**1**]CF_3_SO_3_ (0.8% *v*/*v* DMSO in water) following exposure to 350 nm radiation could be tentatively assigned to a CO/DMSO exchange process. However, jointly with K[**2**] and [**5**]CF_3_SO_3_ (in a MeCN or H_2_O solution, respectively), only minor changes in the IR spectra are observed in the first 10 min (Figures S27–S29). Instead, irradiation of the dicarbonyl PTA-substituted aminocarbyne [**4**]CF_3_SO_3_ (in MeCN) caused substantial variations in the carbonyl absorption pattern, accompanied by the appearance of new bands at 2010 and 2056 cm^–1^ (Figure S30) suggestive of the formation of a piano-stool Fe(II) complex by oxidative cleavage of the Fe(I)–Fe(I) precursor.^30^ For instance, bands at 2005 and 2047 cm^–1^ were reported for the related [Fe(η^5^-indenyl)(CO)_2_(PTA)]^+^.^30b^ These data indicate that dicarbonyl aminocarbyne cations may undergo a different photoactivation mechanism compared to their tricarbonyl precursors.

The highest carbonyl stretching wavenumber, in a CH_2_Cl_2_ or MeOH solution, decreases along the following sequence: [**1**]CF_3_SO_3_ (2038 cm^–1^) > [**3**]NO_3_ (2022 cm^–1^) > [**5**]CF_3_SO_3_ (1993 cm^–1^) > K[**2**] (1991 cm^–1^) > [**4**]CF_3_SO_3_ (1977 cm^–1^). Based on this scale, the sensitivity to 350 nm irradiation is not correlated with the Fe–CO bond strength.^30^ Looking at the electronic transitions in an aqueous solution, the photoactivable aminocarbyne complexes **3**^**+**^ and **4**^**+**^ display an absorption peak (λ_max_ 340 nm) close to the irradiation wavelength (350 nm), while compounds that are minimally photoresponsive (**1**^**+**^, **2**^–^, and **5**^**+**^) are characterized by blue-shifted absorptions (311, 335, and 308 nm, respectively). Thus, 350 nm radiation is sufficient to activate **3**^**+**^ and **4**^**+**^, while a higher energy UV treatment would probably be required for the other complexes.

### 2.3 Cell Proliferation

**Figure 5:**
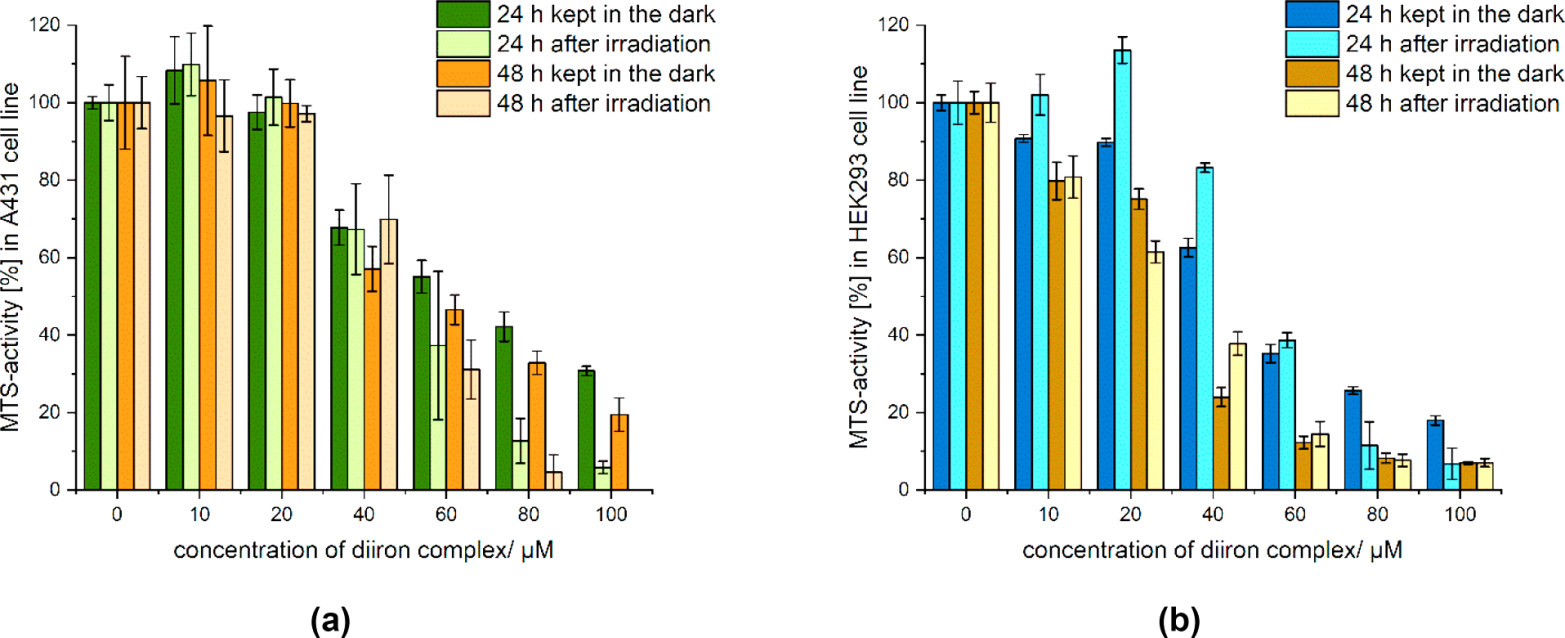
MTS assays of [**1**]CF_3_SO_3_ (0–100 μM) in A431 (**a**) and HEK293 (**b**) cells with or without a 10-min exposure to 350 nm radiation (*E*_v_ = 6 mW/cm^2^). Cell viability was measured after 24 and 48 h, respectively, at 37 °C, and expressed relative to untreated cells (ANOVA at α = 0.05). IC_50_ (A431 cells, 48 h) ≈ 49 μM (dark), 52 μM (irradiated). IC_50_ (HEK293 cells, 48 h) ≈ 32 μM (dark), 33 μM (irradiated).

**Figure 6:**
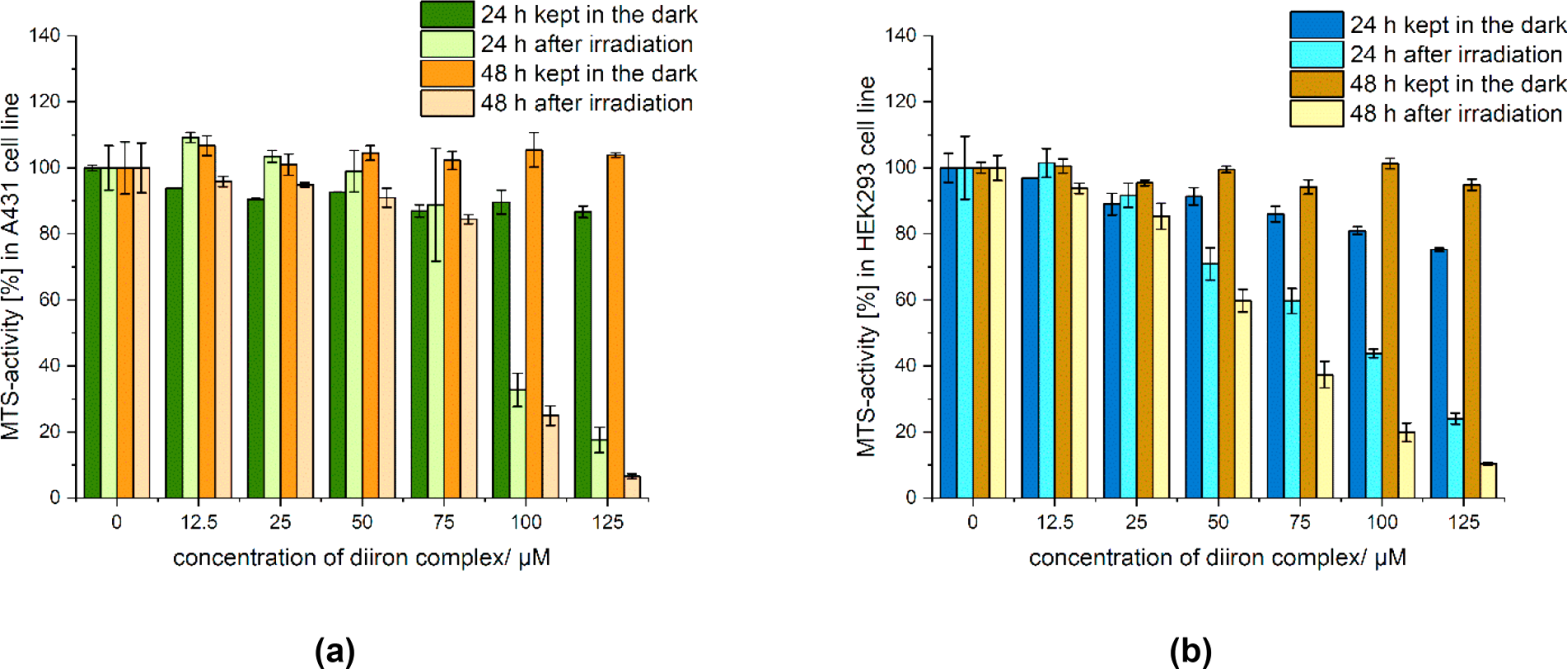
MTS assays of [**3**]NO_3_, **NIRAC**, (0–125 μM) in A431 (**a**) and HEK293 (**b**) cells with or without a 10-min exposure to 350 nm radiation (*E*_v_ = 6 mW/cm^2^). Cell viability was measured after 24 and 48 h, respectively, at 37 °C, and expressed relative to untreated cells (ANOVA at α = 0.05). IC_50_ (A431 cells, 48 h) > 125 μM (dark), ≈90 μM (irradiated). IC_50_ (HEK293 cells, 48 h) > 125 μM (dark), 70 μM (irradiated).

Proliferation experiments were carried out on A431 (human skin cancer) and HEK293 (nontumoral human embryonic kidney) cells treated with increasing concentrations (0–100/125 μM) of the diiron compounds dissolved in water/DMSO 0.8% *v*/*v* ([**1**]CF_3_SO_3_, [**4**]CF_3_SO_3_, [**5**]CF_3_SO_3_) or PBS (K[**2**], [**3**]NO_3_). Experiments were performed in 48-well plates exposed to 350 nm radiation (*E*_v_ ≈ 6 mW/cm^2^) for 10 min at 37 °C. Controls (without addition of diiron complexes) were likewise treated with water/DMSO or PBS, kept in the dark, or irradiated. Cell viability was assessed with the MTS assay after 24 and 48 h of incubation at 37 °C; results are displayed in Figures 5, 6, and S31–S37.

Compound [**1**]CF_3_SO_3_ is moderately cytotoxic on both cell lines kept in the dark (IC_50_ after 48 h: 50 μM on A431, 30 μM on HEK293; Figure 5). The other complexes are substantially nontoxic without exposure to 350 nm radiation against A431 cancer cells up to the maximum concentration tested.

A similar scenario is observed for nonirradiated HEK293 cells, except for a significant reduction in cell viability upon treatment with the highest concentrations (60–100 μM) of K[**2**] for 48 h and a more modest effect for [**4**]CF_3_SO_3_ in the same conditions. Overall, cell viability profiles (*dark* cytotoxicity) for [**1**]CF_3_SO_3_, [**3**]NO_3_, and [**5**]CF_3_SO_3_ resemble those previously found on other cancerous and noncancerous cell lines for the same species (**3**^**+**^ as CF_3_SO_3_^–^ salt).^17a,18a^

Exposure to 350 nm radiation resulted in minor differences in A431 and HEK293 cell viability for [**1**]CF_3_SO_3_, K[**2**], and [**5**]CF_3_SO_3_, up to the maximum concentration tested, in agreement with their modest or absent response to 350 nm irradiation (see above).^31^ Conversely, a marked decrease in viability (>50%) was ascertained for A431 cells treated with [**3**]NO_3_, **NIRAC** (100 and 125 μM), the same compound being inactive in the absence of irradiation under the same conditions (Figure 6a). Compound [**3**]NO_3_ manifested a photoinduced cytotoxicity also toward nontumoral HEK293 cells (Figure 6b); in this respect, higher sensitivity of this cell line to carbon monoxide was previously reported.^5d^ Note that the irradiation time used for cell viability experiments is compatible with the kinetics of the photoactivation of [**3**]NO_3_ (see Figures 3 and 4), indicating that the photoproducts are responsible for cytotoxicity.

A decrease in viability of A431 cells upon irradiation can be also noticed for [**4**]CF_3_SO_3_ after a 24-h incubation (≥60 μM; Figure S34a); nevertheless, the higher viability observed after 48 h suggests caution on interpreting these data.

## 3 Conclusions

Here, we have presented one of the first studies on the photoinduced cytotoxicity of cyclopentadienyl diiron complexes. More specifically, five hydrophilic compounds with different bridging hydrocarbyl ligands were investigated for their antiproliferative activity and photoactivation under UV irradiation. This series includes three novel compounds, which were designed to enhance the aqueous solubility. The tricarbonyl aminocarbyne compound **NIRAC**, readily accessible in multigram scales from inexpensive precursors and very soluble in water thanks to the nitrate anion, emerged with the best performance, being nontoxic in the dark but able to decrease cell viability in a dose-dependent manner upon irradiation at 350 nm. In detail, photoexcitation of **NIRAC** using low power near-UV light promotes carbon monoxide release which triggers the fast disruption of the diiron framework, according to kinetics well-fitting the short irradiation treatment (10 min) used in cellular studies. The other compounds manifested a lower sensitivity to irradiation in terms of cellular effects, which is not correlated to their Fe–CO bond strength (IR spectra). These preliminary results encourage further studies on the photoactivation of diiron complexes based on the {Fe_2_Cp_2_} core in the medicinal (anticancer) setting, taking advantage of their easy availability and wide structural variability^32^ to design new compounds that can be activated by longer, biocompatible wavelengths.

## 4 Experimental Section

### 4.1 General Experimental Details

[Fe_2_Cp_2_(CO)_4_] (99%) was purchased from Strem Chemicals; other reactants and solvents were obtained from Alfa Aesar, Merck, Apollo Scientific, or TCI Chemicals and were of the highest purity available. Ethyl triflate and methyl iodide (4 °C), ethyl isocyanoacetate (−20 °C), and 1,3,5-triaza-7-phosphatricyclo[3.3.1.1]decane (PTA) were stored under N_2_. Contaminated labware was treated with NaOH/EtOH (alkylating agents) or HCl/EtOH (isocyanides). Complexes [Fe_2_Cp_2_(CO)_2_(μ-CO)(μ-CS)],^33^ [Fe_2_Cp_2_(CO)_3_(CNMe)],^12^ [Fe_2_Cp_2_(CO)_2_(μ-CO){μ-CNMe(Xyl)}]CF_3_SO_3_,^13^ and [Fe_2_Cp_2_(CO)(μ-CO){μ–η^1^:η^3^-C(4-C_6_H_4_CO_2_H)CHCNMe_2_}]CF_3_SO_3_ ([**5**]CF_3_SO_3_)^18a^ were prepared according to published procedures. The synthesis of [**1**]CF_3_SO_3_, K[**2**], and [**3**]NO_3_ was carried out under dry N_2_ using standard Schlenk techniques and solvents distilled over appropriate drying agents (MeCN from CaH_2_, CH_2_Cl_2_ from P_2_O_5_). The synthesis of potassium isocyanoacetate, [**4**]CF_3_SO_3_, and [**6**]CF_3_SO_3_ was carried out under N_2_ using deaerated solvents. All the other operations were conducted under air with common laboratory glassware. Chromatographic separations were carried out on neutral alumina columns (Merck). NMR spectra were recorded at 25 °C on a Bruker Avance II DRX400 instrument equipped with a BBFO broadband probe. Chemical shifts are referenced to the residual solvent peaks (^1^H, ^13^C) or to external standards (^14^N to CH_3_NO_2_, ^19^F to CFCl_3_, ^31^P to 85% H_3_PO_4_).^34^^1^H and ^13^C spectra were assigned with the assistance of ^1^H{^31^P}, ^1^H NOESY, and ^1^H–^13^C *gs*-HSQC experiments. NMR signals due to minor isomers are italicized. IR spectra of solid samples (650–4000 cm^–1^) were recorded on a PerkinElmer Spectrum One FT-IR spectrometer equipped with a UATR sampling accessory; IR spectra of solutions were recorded on a PerkinElmer Spectrum 100 FT-IR spectrometer using a CaF_2_ liquid transmission cell (2300–1500 cm^–1^). IR and UV–vis spectra were processed with Spectragryph software.^35^ Carbon, hydrogen, nitrogen, and sulfur analyses were performed on a Vario MICRO cube instrument (Elementar).

### 4.2 Synthesis and Characterization of Compounds

#### [Fe_2_Cp_2_(CO)_2_(μ-CO)(μ-CSEt)]CF_3_SO_3_, [**1**]CF_3_SO_3_ (Chart 1)

The synthesis of related tetrafluoroborate and iodide salts was previously reported.^11a,11b^ The CF_3_SO_3_^–^ salt was reported without the synthetic procedure.^36^ Compound [Fe_2_Cp_2_(CO)_2_(μ-CO)(μ-CS)] (*ca*. 0.6 mmol, in admixture with [Fe_2_Cp_2_(CO)_4_]) was dissolved in anhydrous CH_2_Cl_2_ (8 mL) under N_2_ and treated with CF_3_SO_3_Et (0.10 mL, 0.77 mmol). The dark-red mixture was stirred for 6 h at room temperature and then transferred on top of an alumina column (h 3, d 3.4 cm). Impurities were eluted with neat CH_2_Cl_2_ and THF, and then a red band was collected with MeCN. Volatiles were removed under vacuum, and the residue was triturated after prolonged soaking in Et_2_O/hexane (1:1 v/v mixture, 50 mL). The suspension was filtered; the resulting carmine red solid was washed with Et_2_O/hexane and dried under vacuum (40 °C). Yield: 214 mg, 65%. Soluble in CH_2_Cl_2_, CHCl_3_, acetone, MeCN, sparingly soluble in Et_2_O, insoluble in hexane. Anal. Calcd for C_17_H_15_F_3_Fe_2_O_6_S_2_: C: 37.25, H: 2.76, S: 11.70. Found: 37.10, H: 2.61, S: 11.52. IR (solid state): υ̃/cm^–1^ = 3102w, 2974w, 2938w, 2026s (CO), 2003s (CO), 1837s (μ-CO), 1454w, 1432w, 1420w, 1382w, 1362w, 1275s-sh, 1258s (SO_3_), 1225s-sh (SO_3_), 1152s (SO_3_), 1029s (CS), 999s-sh, 973w, 910w, 860m, 850m, 767w, 755w, 700m-sh, 688s. IR (CH_2_Cl_2_): υ̃/cm^–1^ = 2038s (CO), 2007m-sh (CO), 1850m (μ-CO). IR (MeCN): υ̃/cm^–1^ = 2038s (CO), 2006m-sh (CO), 1850m (μ-CO). ^1^H NMR (acetone-*d*_6_): δ/ppm = 5.72, 5.64 (s, 10H, Cp); 4.41, 4.17 (dq, ^2^*J*_HH_ = 14.8 Hz, ^3^*J*_HH_ = 7.5 Hz, 2H, CH_2_); 1.67 (t, ^3^*J*_HH_ = 7.6 Hz, 3H, CH_3_). No changes in the ^1^H NMR spectrum were observed after 14 h at room temperature. ^13^C{^1^H} NMR (acetone-*d*_6_): δ/ppm = 405.5 (CS); 252.2 (μ-CO); 208.1, 207.9 (CO); 122.5 (d, ^1^*J*_CF_ = 322 Hz, CF_3_); 93.0, 92.3 (Cp); 50.5 (CH_2_), 13.5 (CH_3_). ^19^F{^1^H} NMR (acetone-*d*_6_): δ/ppm = −78.8.

**Chart 1:**
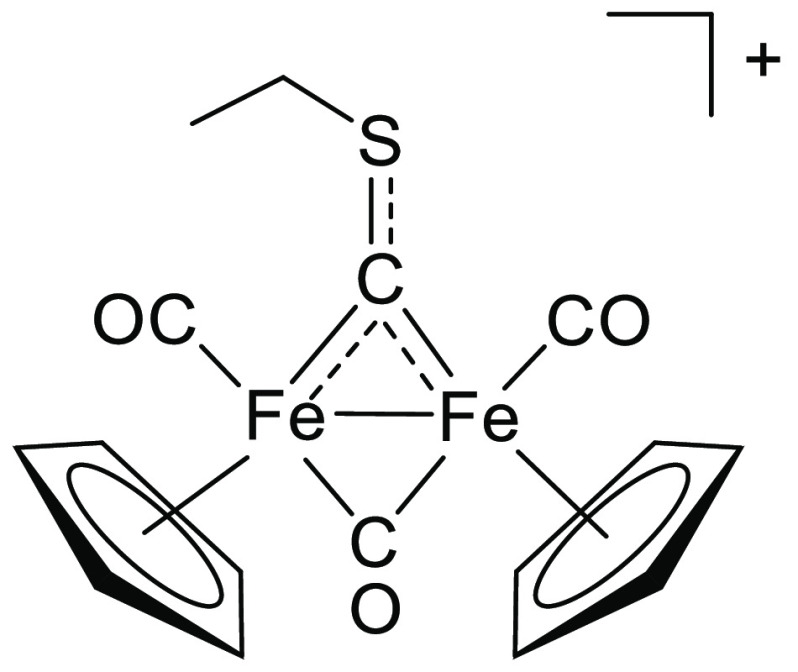
Structure of **1**^**+**^

#### Potassium 2-Isocyanoacetate, K[C≡NCH_2_CO_2_] (Chart 2)

The title compound was prepared according to a modified literature procedure.^37^ A solution of KOH (395 mg, 7.0 mmol) in water (1 mL) was added to a solution of ethyl isocyanoacetate (0.76 mL, 6.95 mmol) in THF (10 mL) under N_2_. The pale orange suspension was stirred for 5 h at room temperature under protection from the light. The final mixture (two pale yellow immiscible liquids) was dried under vacuum, and the residue was triturated in MeCN (20 mL). The suspension was filtered; the resulting colorless solid was thoroughly washed with MeCN and then Et_2_O and dried under vacuum at 40 °C, over P_2_O_5_. Yield: 755 mg, 88%. The solid was kept under N_2_ at −20 °C for long-term storage. Soluble in water, MeOH, DMSO; insoluble in all other common organic solvents. Anal. Calcd for C_3_H_2_KNO_2_: C, 29.26; H, 1.64; N, 11.37. Found: C, 28.51; H, 1.78; N, 11.21. IR (solid state): υ̃/cm^–1^ = 3505w, 3330w, 3246w, 2174w-sh, 2157s (CN), 1609s-br (CO_2,asym_), 1577s-sh, 1428w, 1417w, 1394s (CO_2,sym_), 1369s, 1296s, 1261w, 1236w, 972m, 902s, 696s. IR (MeOH): υ̃/cm^–1^ = 2177w-sh (CN), 2156m (CN), 1636s (CO_2_). ^1^H NMR (DMSO-*d*_6_): δ/ppm = 3.70 (s, CH_2_). ^1^H NMR (CD_3_OD): δ/ppm = 4.05 (s, CH_2_). ^13^C{^1^H} NMR (CD_3_OD): δ/ppm = 170.4 (CN), 44.4 (m*, CH_2_) [*H/D exchange]. Commercially available potassium isocyanoacetate (Merck, 85%) contains *potassium N-formyl glycinate*, which cannot be easily removed due to similar solubility properties. ^1^H NMR (CD_3_OD): δ/ppm = 8.07 (s, 1H, HCO), 3.77 (s, 2H, CH_2_).

**Chart 2:**
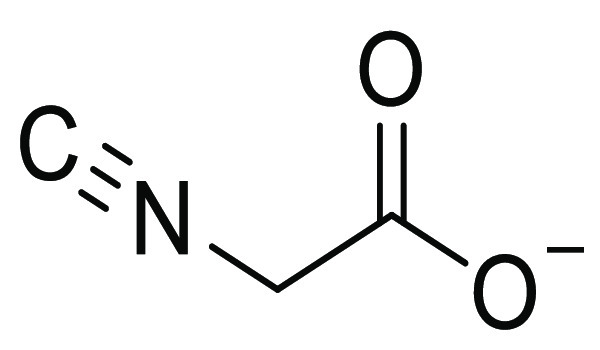
Structure of [C≡NCH_2_CO_2_]^−^

#### K[Fe_2_Cp_2_(CO)_3_(CNCH_2_CO_2_)], K[**2**] (Chart 3)

The organometallic anion was previously isolated as the [K(18-crown-6)]^+^ salt in a two-step procedure with [Fe_2_Cp_2_(CO)_3_(NCMe)] as the intermediate.^19^ In a Schlenk flask under N_2_, a mixture of [Fe_2_Cp_2_(CO)_4_] (283 mg, 0.80 mmol) and potassium 2-isocyanoacetate (99 mg, 0.80 mmol) in anhydrous MeCN (6 mL) was stirred at reflux for 5 h. The resulting suspension (red solid and dark red solution) was filtered over a Celite column, and the solid was washed thoroughly with MeCN and Et_2_O, to ensure complete removal of unreacted [Fe_2_Cp_2_(CO)_4_]. Next, the solid on the column was dissolved and eluted using deaerated MeOH under N_2_. The methanolic eluate was dried under vacuum, affording a raspberry red/purple glassy solid. Yield: 222 mg, 62%. The solid was kept under N_2_ for long-term storage (hygroscopic). The reaction is very sensitive to moisture: rigorously anhydrous MeCN is required. The addition of protic species (*e.g*., MeOH, [Et_3_NH]Cl) results in complete recovery of [Fe_2_Cp_2_(CO)_4_], likely due to the instability of CNCH_2_CO_2_H.^38^ Soluble in water, MeOH, EtOH, DMSO, poorly soluble in DMF, insoluble in MeCN, CH_2_Cl_2_, Et_2_O, and other common organic solvents. Anal. Calcd for C_16_H_12_Fe_2_KNO_5_: C, 42.79; H, 2.69; N, 3.12. Found: C, 42.22; H, 2.88; N, 3.05. IR (solid state): υ̃/cm^–1^ = 3111w, 2967w, 2931w; 2202w-sh*, 2140s (C≡N); 1979m-sh, 1933s (CO); 1778m-sh, 1737s-sh (μ-CO); 1719s (μ-CN); 1618s, 1595s (CO_2,asym_); 1417m, 1370s (CO_2,sym_), 1286s, 1114w, 1059w, 1012m, 1004m, 977w, 900w, 839m-sh, 817m, 969w. IR (MeOH): υ̃/cm^–1^ = 2204*w-sh, 2106m (C≡N); 1991s, 1951m (CO); 1797m, 1762w (μ-CO); 1724m (μ-CN), 1670m; 1635s (CO_2,asym_) [*CpFe(II) *impurity*]. ^1^H NMR (DMSO-*d*_6_): δ/ppm = 4.74, 4.66 (s-br, 10H, Cp); 4.03 (br, 2H, CH_2_). ^13^C{^1^H} NMR (DMSO-*d*_6_): δ/ppm = 279.3 (μ-CO); 213.9 (CO); 165.0 (br, CO_2_); 158.7, 156.3 (CN); 87.9, 87.1 86.4 (Cp); 50.0, 49.2 (br, CH_2_). ^1^H NMR (CD_3_OD): δ/ppm = 4.97, 4.95 (s-br, 10H, Cp), 4.30 (s, 2H,** CH_2_) [**undergoes H/D exchange]. ^13^C{^1^H} NMR (CD_3_OD): δ/ppm = 213br (CO), 170 (CN), 160.7 (CO_2_), 88.8, 83.3 (Cp); the CH_2_ signal is hidden by the solvent residual peak.

**Chart 3:**
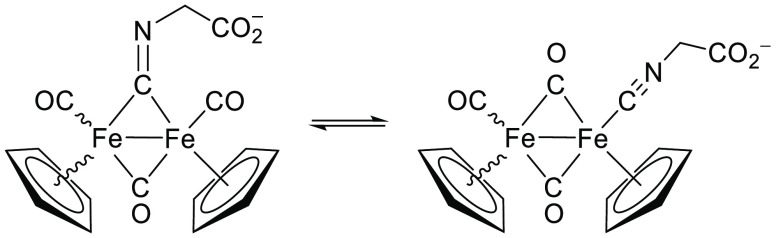
Structures of **2**^–^a

a Wavy bonds represent *cis*/*trans* isomerism.

#### [Fe_2_Cp_2_(CO)_2_(μ-CO)(μ-CNMe_2_)]NO_3_, [**3**]NO_3_, **NIRAC** (Chart 4)

Compound [Fe_2_Cp_2_(CO)_3_(CNMe_2_)]I was prepared according to a modified literature procedure.^39^ A solution of [Fe_2_Cp_2_(CO)_3_(CNMe)]^12^ (*ca*. 3.5 mmol, from methyl isocyanide) in CH_2_Cl_2_ (30 mL) was treated with methyl iodide (1.3 mL, 22 mmol) and stirred at room temperature under N_2_ for 72 h. The final solution was charged on an alumina column. Impurities were eluted using CH_2_Cl_2_, and then a red fraction corresponding to [Fe_2_Cp_2_(CO)_2_(μ-CO)(μ-CNMe_2_)]I was eluted with CH_3_OH. AgNO_3_ (594 mg, 3.5 mmol) was added to the eluted methanol solution; the mixture was stirred for 1 h, during which time progressive precipitation of a white solid (AgI) occurred. The final mixture was dried under reduced pressure. The residue was suspended in MeCN/CH_2_Cl_2_ 1:2 *v*/*v* and filtered through a Celite pad. Subsequent evaporation of the solvent under reduced pressure afforded a dark red solid, which was washed with Et_2_O and hexane and dried under vacuum (40 °C). Yield: 1087 mg, 70%. Soluble in water, MeOH, MeCN, less soluble in acetone, CH_2_Cl_2_, insoluble in Et_2_O, hexane. Anal. Calcd for C_16_H_16_Fe_2_N_2_O_6_: C, 43.25; H, 3.63; N, 6.31. Found: C, 43.15; H, 3.57; N, 6.41. IR (solid state): υ̃/cm^–1^ = 3398br, 3112sh, 3081w-m, 2943w, 2870vw, 2211w, 2176w-m; 1992vs (CO), 1972vs (CO), 1940m-sh, 1825vs (μ-CO), 1790m-sh, 1667w-m, 1607s (μ-CN), 1448w, 1433w, 1418w, 1396m, 1366m-s, 1328br-vs (NO_3_), 1191m-s, 1163m-s, 1116vw, 1067br-w, 1026m, 1008m, 952w, 860s, 829m, 760br-vs IR (MeCN): υ̃/cm^–1^ = 2022s (CO), 1989m (CO), 1833s (μ-CO), 1603m (μ-CN). ^1^H NMR (CD_3_OD): δ/ppm = 5.38, 5.26 (s, 10H, Cp); 4.31, 4.23 (s, 6H, NCH_3_). ^1^H NMR (D_2_O): δ/ppm = 5.34, 5.22 (s, 10H, Cp); 4.27, 4.19 (s, 6H, NCH_3_). Isomer (*cis*/*trans*) ratio (^1^H NMR): 8.8 (CD_3_OD), ca. 22 (D_2_O). ^13^C{^1^H} NMR (D_2_O): δ/ppm = 260.2 (μ-CO); 207.6 (CO); 91.2, 89.7 (Cp); 53.5 (NCH_3_); the signal for μ-CN falls outside the selected spectral window (>300 ppm). ^14^N NMR (D_2_O): δ/ppm = −5.1 (s, Δν_1/2_ = 19 Hz, NO_3_^–^).

**Chart 4:**
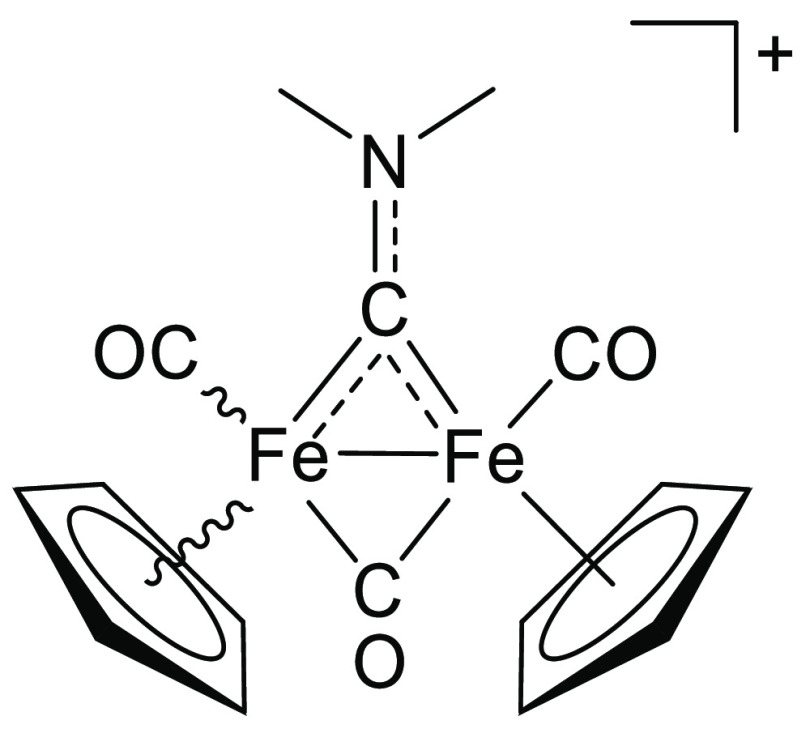
Structure of **3**^**+**^a

a Wavy bonds represent *cis*/*trans* isomerism.

**[3]CF**_**3**_**SO**_**3**_.^13^ The solid-state IR was recorded for comparative purposes. IR (solid state): υ̃/cm^–1^ = 3126w, 3097w, 2945w, 2174w, 2021s (CO), 1991s (CO), 1958m-sh, 1814s (μ-CO), 1774w-sh, 1592s (μ-CN), 1454w, 1440w, 1422w, 1393m, 1279s-sh, 1257s (SO_3_), 1223s-sh (SO_3_), 1186m, 1149s (SO_3_), 1069w, 1031s, 1009m-sh, 881m-sh, 872m, 848m, 837m-sh, 761s, 755s-sh.

#### [Fe_2_Cp_2_(CO)(PTA)(μ-CO){μ-CNMe(Xyl)}]CF_3_SO_3_, [**4**]CF_3_SO_3_ (Chart 5)

A solution of [Fe_2_Cp_2_(CO)_2_(μ-CO){μ-CNMe(Xyl)}]CF_3_SO_3_ (87 mg, 0.14 mmol) and Me_3_NO·2H_2_O (17 mg, 0.15 mmol) in MeCN (5 mL) was stirred at room temperature under N_2_. After 1 h, conversion was checked by IR, and then volatiles were removed under vacuum. The dark brown residue was dissolved in THF (10 mL), and PTA (24 mg, 0.15 mmol) was added. The solution was stirred at reflux temperature under N_2_ for 2 h, progressively turning dark green with precipitate formation. Conversion was checked by IR and then the suspension was cooled at room temperature and filtered (G4 glass filter). The dark green-brown solid was washed with THF, Et_2_O, and hexane and dried under vacuum (40 °C). Yield: 92 mg, 87%. Soluble in water, DMSO, MeCN, MeOH, poorly soluble in THF, CH_2_Cl_2_, acetone, ^i^PrOH; insoluble in toluene, Et_2_O, hexane. X-ray quality crystals of [**4**]CF_3_SO_3_ (*Z* isomer) were obtained from a MeCN solution layered with Et_2_O and settled aside at −20 °C. Anal. Calcd for C_29_H_34_F_3_Fe_2_N_4_O_5_PS: C, 46.42; H, 4.57; N, 7.47; S, 4.27. Found: C, 46.5; H, 4.44; N, 7.32; S, 4.36. IR (solid state): υ̃/cm^–1^ = 3101w, 3080w, 2952w, 2928w, 2880w, 1992s (CO), 1947w-sh (CO), 1810s (μ-CO), 1773w, 1506m-sh (μ-CN), 1494m, 1471w, 1448w, 1436w, 1417w, 1380m, 1290w, 1259s (SO_3_), 1244s-sh, 1221s (SO_3_), 1172m-sh, 1151s (SO_3_), 1106m, 1084m, 1044w, 1027s, 1015s, 973s, 948s, 888m, 853m, 843m, 805m, 787m, 768s, 743m, 733s, 633s. IR (CH_2_Cl_2_): υ̃/cm^–1^ = 1977m (CO), 1794m (μ-CO). IR (MeCN): υ̃/cm^–1^ = 1974s (CO), 1792m (μ-CO), 1508w (μ-CN). ^1^H NMR (acetone-*d*_6_): δ/ppm = 7.50–7.40 (m, 3H, C^3^H + C^4^H); 5.51, 4.73 (s, 5H, Cp); 5.33, 4.87 (d, ^3^*J*_HP_ = 1.5 Hz, 5H, Cp^P^); 4.58, 4.53 (s, 3H, NCH_3_); 4.44–4.31 (m, 6H, NCH_2_); 4.09 (s), 3.79, 3.68 (d/m, ^2^*J*_HH_ = 15 Hz) (6H, PCH_2_); 2.79, 2.75, 2.41, 2.35 (s, 6H, C^5^H). Isomer (*Z*/*E*) ratio *ca*. 10 (^1^H NMR, acetone-*d*_6_). ^13^C{^1^H} NMR (acetone-*d*_6_): δ/ppm = 331.1 (d, ^2^*J*_CP_ = 14 Hz, μ-CN); 264.4 (d, ^2^*J*_CP_ = 19 Hz, μ-CO); 215.3 (CO); 149.1 (C^1^); 135.2, 134.6, 133.7, 132.7 (C^2^); 131.1, 130.5 (C^4^); 130.3, 130.2, 130.18, 130.0 (C^3^); 90.9, 89.9 (Cp); 88.5, 87.8 (Cp^P^); 72.9, 72.8 (d, ^3^*J*_CP_ = 7 Hz, NCH_2_); 56.5, 56.1 (NCH_3_); 55.5, 54.5 (d, ^1^*J*_CP_ = 13 Hz, PCH_2_); 20.0, 19.2, 19.0, 18.8 (C^5^). ^19^F{^1^H} NMR (acetone-*d*_6_): δ/ppm = −78.8. ^31^P{^1^H} NMR (acetone-*d*_6_): δ/ppm = −18.9, −22.8.

**Chart 5:**
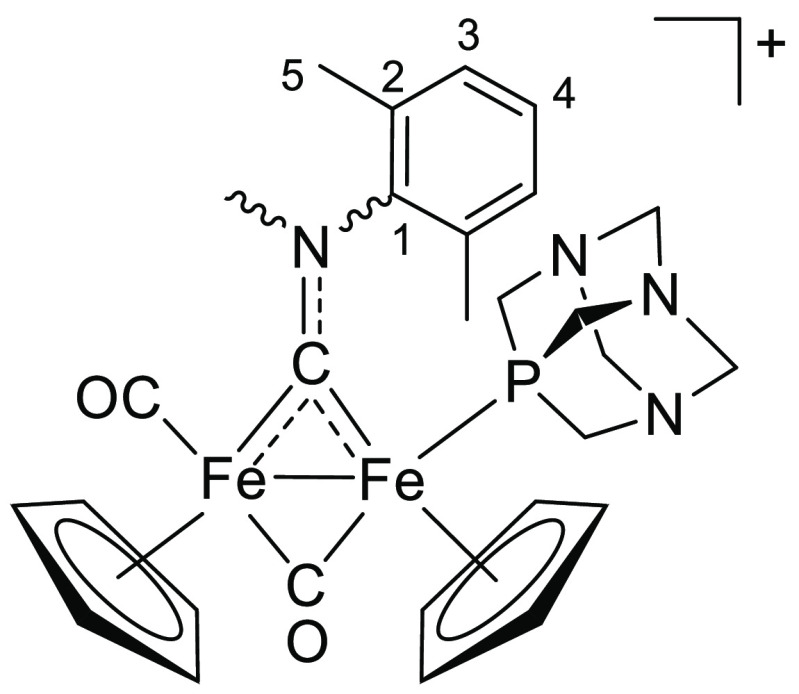
Structure of **4**^**+**^a

a Numbering refers to C atoms; wavy bonds represent *E*/*Z* isomerism.

#### [Fe_2_Cp_2_(CO)(κS-DMSO)(μ-CO)(μ-CNMe_2_)]CF_3_SO_3_, [**6**]CF_3_SO_3_ (Chart 6)

A solution of [Fe_2_Cp_2_(CO)_2_(μ-CO)(μ-CNMe_2_)]CF_3_SO_3_ (121 mg, 0.228 mmol) and Me_3_NO·2H_2_O (17 mg, 0.15 mmol) in THF (8 mL) was treated with DMSO (0.10 mL, 1.4 mmol) and stirred at reflux under N_2_. After 4 h, conversion was checked by IR (CH_2_Cl_2_), and the brown suspension was moved on top of an alumina column (h 4, d 2.3 cm). Impurities were eluted with THF, and then a dark green-brown band was eluted with MeOH/THF 2:1 *v*/*v*. Volatiles were removed under vacuum, and the residue was dissolved in CH_2_Cl_2_ and filtered over Celite. The filtrate was thoroughly dried under vacuum (40 °C) and then triturated in Et_2_O. The suspension was filtered, and the resulting dark brown solid was washed with Et_2_O and pentane and dried under vacuum (40 °C). Yield: 100 mg, 75%. Soluble in CH_2_Cl_2_, acetone; insoluble in Et_2_O, pentane; soluble but not stable in CDCl_3_. Anal. Calcd for C_18_H_22_F_3_Fe_2_NO_6_S_2_: C, 37.20; H, 3.82; N, 2.41; S, 11.03. Found: C, 37.28; H, 3.76; N, 2.37; S, 11.0. IR (solid state): υ̃/cm^–1^ = 3092w, 2981w, 2928w, 2178w, 1971s (CO); 1804m-sh, 1790s (μ-CO); 1592m (μ-CN), 1434w, 1420w, 1397m, 1364w, 1323w, 1257s (SO_3_), 1224s-sh (SO_3_), 1192m, 1141s (SO_3_) 1093s-sh (SO), 1069m-sh, 1029s, 1013s-sh, 970m-sh, 856m, 843m, 757s, 680m. IR (CH_2_Cl_2_): υ̃/cm^–1^ = 2005s (CO), 1980m-sh (CO), 1804s (μ-CO), 1587m (μ-CN). IR (MeCN): υ̃/cm^–1^ = 2002s (CO), 1805s (μ-CO), 1587m (μ-CN). ^1^H NMR (acetone-*d*_6_): δ/ppm = 5.34, 5.28 (s, 5H, Cp); 5.21, 5.17 (s, 5H, Cp′); 4.61, 4.34 (s, 3H, NCH_3_); 4.31, 4.27 (s, 3H, NCH_3_′); 3.58, 3.26 (s, 3H, SCH_3_); 3.19, 3.03 (s, 3H, SCH_3_′). Isomer (*cis*/*trans*) ratio: *ca*. 19 (^1^H NMR, acetone-*d*_6_). ^13^C{^1^H} NMR (acetone-*d*_6_): δ/ppm = 326.5 (μ-CN); 268.0 (μ-CO); 212.4 (CO); 90.3 (Cp); 88.9 (Cp′); 54.8 (NMe′); 53.6 (NMe); 52.9 (SMe); 49.3 (SMe′). ^19^F NMR (acetone-*d*_6_): δ/ppm = −78.7.

**Chart 6:**
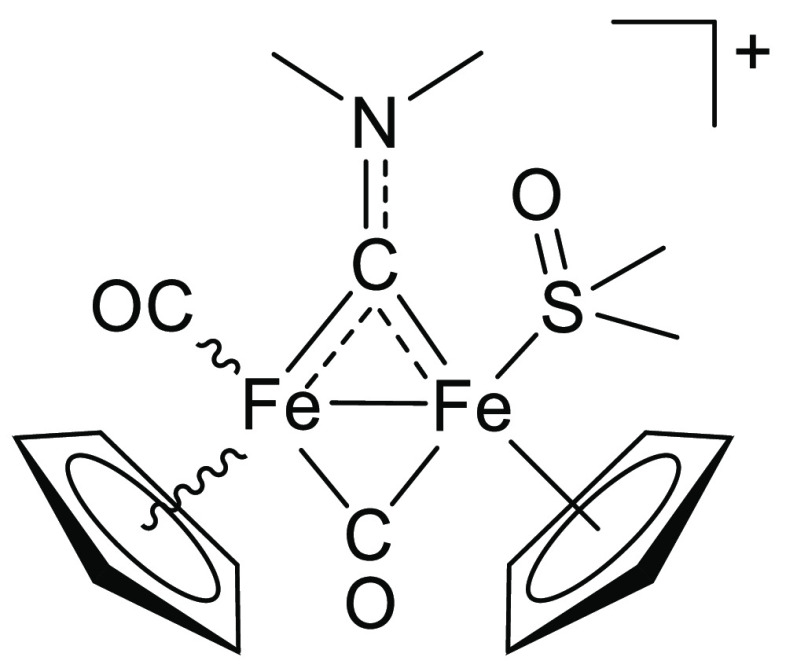
Structure of **6**^**+**^a

a Wavy bonds represent *cis*/*trans* isomerism.

#### ^1^H/^13^C NMR and UV–Vis Spectra in Aqueous Solution

UV–vis spectra (250–800 nm) were recorded on an Ultraspec 2100 Pro spectrophotometer using PMMA cuvettes (1 cm path length).

[**1**]CF_3_SO_3_. UV (H_2_O, 1.7 × 10^–3^ M): λ/nm (ε/M^–1^·cm^–1^) = 280sh (1.1·10^4^), 311sh (8.7·10^3^), 500sh (9.5·10^2^). ^1^H NMR (D_2_O): δ/ppm = 5.46, 5.41 (s, 10H, Cp); 4.19, 4.01 (m, 2H, CH_2_); 1.62 (t, ^3^*J*_HH_ = 7.6 Hz, 3H, CH_3_).

K[**2**]. UV (H_2_O, 1.8 × 10^–3^ M): λ/nm (ε/M^–1^·cm^–1^) = 335 (4.6·10^3^), 510 (3.9·10^2^). ^1^H NMR (D_2_O) δ/ppm = 5.1, 4.9–4.8 (br, Cp), 4.5, 4.4 (br, CH_2_). Two sets of signals are observed in the ^13^C{^1^H} spectrum, ascribable to compounds with a bridging or terminal isocyanide ligand, by comparison with literature data.^12^ [Fe_2_Cp_2_(μ-CO)_2_(CO)(CNCH_2_CO_2_)]^−^. ^13^C{^1^H} NMR (D_2_O): δ/ppm = 293.8 (μ-CO); 211.1 (CO); 171.8 (CO_2_); 160.3 (CN); 87.9, 87.2 (Cp); 48.5 (CH_2_). [Fe_2_Cp_2_(CO)_2_(μ-CO)(μ-CNCH_2_CO_2_)]^−^. ^13^C{^1^H} NMR (D_2_O): δ/ppm = 283.2 (μ-CO); 267 (br, μ-CN); 213 (br, CO); 178.3 (CO_2_); 88.2 (Cp); 64.9 (CH_2_).

[**3**]NO_3_. UV (H_2_O, 1.9 × 10^–3^ M): λ/nm (ε/M^–1^·cm^–1^) = 280sh (8.6·10^3^), 340 (5.5·10^3^), 481 (5.3·10^2^), 533sh (3.8·10^2^). ^1^H NMR (D_2_O): δ/ppm = 5.34, 5.22 (s, 10H, Cp); 4.27, 4.19 (s, 1H, 6H, NCH_3_).

[**4**]CF_3_SO_3_. UV (H_2_O, 3.7 × 10^–4^ M): λ/nm (ε/M^–1^·cm^–1^) = 340sh (3.0·10^3^), 422sh (9.0·10^2^), 510 (4.3·10^2^), 590 (3.1·10^2^). ^1^H NMR (D_2_O): δ/ppm = 7.50–7.32 (m, 3H, C_6_H_3_); 5.38, 5.15 (s, 5H, Cp); 4.73, 4.66 (s, Cp); 4.42–4.23 (m, 9H, NCH_3_ + NCH_2_); 3.92 (s), 3.67 (d, ^2^*J*_HP_ = 16.4 Hz), 3.60–3.53 (m) (6H, PCH_2_); 2.68, 2.63, 2.30, 2.26 (s, 6H, NCH_3_).

[**5**]CF_3_SO_3_. UV (H_2_O, 7.5 × 10^–4^ M): λ/nm (ε/M^–1^·cm^–1^) = 268 (2.6 × 10^4^), 308sh (1.5 × 10^4^), 414sh (3.1 × 10^3^), 526 (7.5 × 10^2^), 607 (5.0 × 10^2^). ^1^H NMR (D_2_O): δ/ppm = 8.03, 7.78 (d, ^3^*J*_HH_ = 8.2 Hz, 4H, C_6_H_4_); 5.27, 5.13 (s, 10H, Cp); 4.62 (s, 1H, CH); 3.88, 3.33 (s, 6H, NCH_3_).

#### Behavior of [**3**]NO_3_ in Water at 37 °C over 72 h

The selected compound was dissolved in a D_2_O solution containing Me_2_SO_2_ (4.0 × 10^–3^ M, 0.7 mL). The red solution (*c*_Fe_2__ ≈ 10^–2^ M) was filtered over Celite and analyzed by ^1^H NMR (delay time = 10 s; number of scans = 20). Next, the solution was heated at 37 °C for 72 h, and NMR analyses were repeated. The residual amount of starting material in the final solution (86%; with respect to the initial spectrum) was calculated by the relative integral of Cp signals with respect to Me_2_SO_2_ as the internal standard. No change in the *cis*/*trans* ratio was observed.

### 4.3 X-ray Crystallography

Crystal data and collection details for [**4**]CF_3_SO_3_·0.5CH_3_CN are reported in Table 1. Data were recorded on a Bruker APEX II diffractometer equipped with a PHOTON100 detector using Mo–Kα radiation. Data were corrected for Lorentz polarization and absorption effects (empirical absorption correction SADABS).^40^ The structure was solved by direct methods and refined by full-matrix least-squares based on all data using *F*^2^.^41^ Hydrogen atoms were fixed at calculated positions and refined by a riding model. All non-hydrogen atoms were refined with anisotropic displacement parameters.

**Table 1:** Crystal Data and Measurement Details for [**4**]CF_3_SO_3_·0.5CH_3_CN

	[**4**]CF_3_SO_3_·0.5CH_3_CN
formula	C_30_H_35.5_F_3_Fe_2_N_4.5_O_5_PS
FW	770.86
*T*, K	100(2)
λ, Å	0.71073
crystal system	monoclinic
space group	*P*2_1_/c
*a*, Å	10.4934(6)
*b*, Å	24.3729(14)
*c*, Å	12.9622(7)
β, deg	111.780(2)
cell volume, Å^3^	3078.5(3)
Z	4
*D*_*c*_, g·cm^–3^	1.663
μ, mm^–1^	1.130
*F*(000)	1588
crystal size, mm	0.21 × 0.18 × 0.14
Θ limits, deg	1.671–26.999
reflections collected	45009
independent reflections	6652 [*R*_int_ = 0.0358]
data/restraints/parameters	6652/23/438
goodness of fit of *F*^2^	1.247
*R*_1_ (*I* > 2σ(*I*))	0.0408
*wR*_2_ (all data)	0.0951
largest diff peak and hole, e Å^–3^	1.336/–0.500

### 4.4 Photolysis Studies

Photolysis experiments were conducted using a CCP-ICH2 photoreactor from Luzchem (https://www.luzchem.com) fitted with 16 deuterium lamps with emission wavelengths centered at 350 nm (fwhm ± 25 nm; *E*_v_ ∼ 6 mW/cm^2^). A quartz fluorescence cuvette from Hellma (*V* = 3 mL; *d* = 1 cm) was used as the reaction vessel.

To determine how much light is absorbed through a photoreaction, ferrioxalate actinometry experiments were performed as described in the literature.^5d,42^ A photon flow (*I*_abs_) of about 3.8 × 10^–8^ Einstein/s was determined. The reaction rate of a photoreaction is best described by eq 1. Conditional rate constants can be derived where pseudo-first-order kinetics is observed
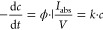
1where *c* represents concentration, *t* represents time,  represents the absorbed light flux density (photon flux per irradiated volume), *A* represents absorbance, and Φ represents quantum yield. The quantum yield can be directly derived when the photon flux is known. For IR studies, 4–5 mg of each sample was dissolved in 3 mL of H_2_O (K[**2**]), 0.8% v/v DMSO in water ([**1**]CF_3_SO_3_, [**3**]NO_3_), or MeCN ([**4**]CF_3_SO_3_, [**5**]CF_3_SO_3_) and irradiated for 5, 10, 30, and 60 min at 350 nm at 37 °C. Aliquots (700 μL) of each time point were lyophilized, and the IR (ATR) spectrum of the solid samples was recorded (1600–2300 cm^–1^).

### Myoglobin Assay

Lyophilized equine heart myoglobin (13 mg, Sigma-Aldrich) was dissolved in 8.5 mL of a 10 mM PBS buffer solution (pH 7.4). The solution was filtered to ensure optical clarity and degassed under nitrogen. A myoglobin solution (2.5 mL) was added to a sealed 3 mL fluorescence cuvette, and *ca*. 3 mg of sodium dithionite (8 mM) was then added. The UV spectrum of the deoxy-Mb solution was then recorded to determine the exact concentration (*A*_560_ = 13800 L·mol^–1^·cm^–1^).^43^ An aliquot of a stock solution of [**3**]NO_3_ (3 mM, 14.2 μL) in 10 mM PBS buffer was then added to the reduced myoglobin solution, with final concentrations of 17 μM [**3**]NO_3_ and 68 μM myoglobin (1:4 molar ratio). The cuvette was sealed with a plug to prevent the escape of CO or reoxidation of the myoglobin. Solutions were initially kept in the dark to study the stability of the complexes in the presence of sodium dithionite for 15 min and then exposed to UV light (350 nm, *E*_v_ = 6 mW/cm^2^, 25 °C). The number of CO equivalents released per mole of diiron complex was calculated as reported.^44^

### 4.5 Cell Experiments

Human A431 (skin epidermoid carcinoma) and HEK293 (human embryonic kidney) cell lines were purchased from CLS (Cell Lines Service GmbH, Eppelheim, Germany) and DSMZ Leibniz Institute (DSMZ–German Collection of Microorganisms and Cell Cultures). The cells were cultivated in Dulbecco’s Modified Eagle Medium (DMEM) without the phenol red indicator and supplemented with heat inactivated 10% fetal bovine serum (FBS), penicillin, and streptomycin. All ingredients of cell medium were purchased from BIOCHROM or Thermo Fisher Scientific. The MTS assay cell experiments were performed in Cellstar transparent flat bottom 48-well plates. The cells were seeded with a density of 10 000 cells per well. Each well contained 200 μL of media. Cells were incubated for 24 h at 37 °C in a humidified atmosphere enriched with 5% CO_2_. The selected diiron compound was added after 24 h of seeding. A freshly prepared 12.5 mM stock solution of the diiron compounds in 100% DMSO ([**1**]CF_3_SO_3_, [**4**]CF_3_SO_3_, and [**5**]CF_3_SO_3_) or in PBS (K[**2**] and [**3**]NO_3_) was diluted with cell medium depending on the final concentration (10/20/40/60/80/100 μM for **1**^**+**^, **2**^**–**^, and **4**^**+**^ and 12.5/25/50/75/100/125 μM for **3**^**+**^ and **5**^**+**^). The final concentration of DMSO in each well was 0.8% *v*/*v*. Two negative controls were used: either DMSO-containing cell medium (0.8% *v*/*v*) or just cell medium with final volumes of 200 μL per well. The outer wells were filled with phosphate buffer (PBS) to prevent the samples from evaporating. After 4 h from treatment with the respective complex, cells were irradiated at 350 nm for 10 min using the Luzchem photoreactor CCP-ICH2 at 37 °C. Fresh DMEM medium (100 μL) without the phenol red indicator was added to each well. The well plates were then stored in the incubator. Control cells were also irradiated, in order to compensate for the cell-killing effects of UV light.^45^ Cell viability was determined using the commercially available Promega CellTiter 96 AQueous One Solution Cell Proliferation Assay MTS kit. Twenty-four and 48 h after the irradiation, 60 μL of the MTS reagent was added to each well and left in the incubator for an additional 1–2 h. The formed formazan was detected at 492 nm using the TECAN microplate reader Sunrise. IC_50_ values were obtained by the fitting four-parameter logistic nonlinear regression model, also known as the Hill equation using GraphPad Prism (version 9.3.0).

## Supporting Information Available

IR, NMR, and UV–vis spectra of compounds in organic solvents, aqueous solution and upon UV irradiation. Cell viability experiments (PDF)
