# Supplementary material for: Switching on Cytotoxicity of Water-Soluble Diiron Organometallics by UV Irradiation
Source: Inorg Chem. 2022 May 10;61(20):7897–909. doi: 10.1021/acs.inorgchem.2c00504 (PMC9951222; doi:10.1021/acs.inorgchem.2c00504)

Supporting Information

Switching On Cytotoxicity of Water Soluble Diiron Organometallics by UV Irradiation

Lorenzo Biancalana,[a] Manja Kubeil,[b] Silvia Schoch,[a] Stefano Zacchini,[c] Fabio Marchetti [a]*

[a] Department of Chemistry and Industrial Chemistry, University of Pisa, Via G. Moruzzi 13, I-56124 Pisa, Italy.

[b] Institute of Radiopharmaceutical Cancer Research, Helmholtz-Zentrum Dresden-Rossendorf, Bautzner Landstrasse 400, 01328 Dresden (Germany)

[c] Department of Industrial Chemistry "Toso Montanari", University of Bologna, Viale del Risorgimento 4, I-40136 Bologna, Italy.

* Email: lorenzo.biancalana@unipi.it

**Table of contents**	**Pages**
Solid state IR spectra (**Figures S1–S5**)	S2–S6
NMR spectra in organic solvents (**Figures S6–S17**)	S7–S12
^{1}H, ^{13}C NMR and UV-Vis spectra in water (D_2O) (**Figures S18–S24**)	S13–S16
^{13}C NMR, IR and UV-vis spectra upon UV irradiation (**Figures S25-S30**)	S17–S20
Cell viability experiments (**Figures S31-S37**)	S21–S26

Solid state IR spectra

Figure S1. Solid-state IR spectrum (650-4000 cm^{-1}) of $[Fe_2Cp_2(CO)_2(\mu\text{-}CO)(\mu\text{-}CSEt)]CF_3SO_3$, [**1**]$CF_3SO_3$.

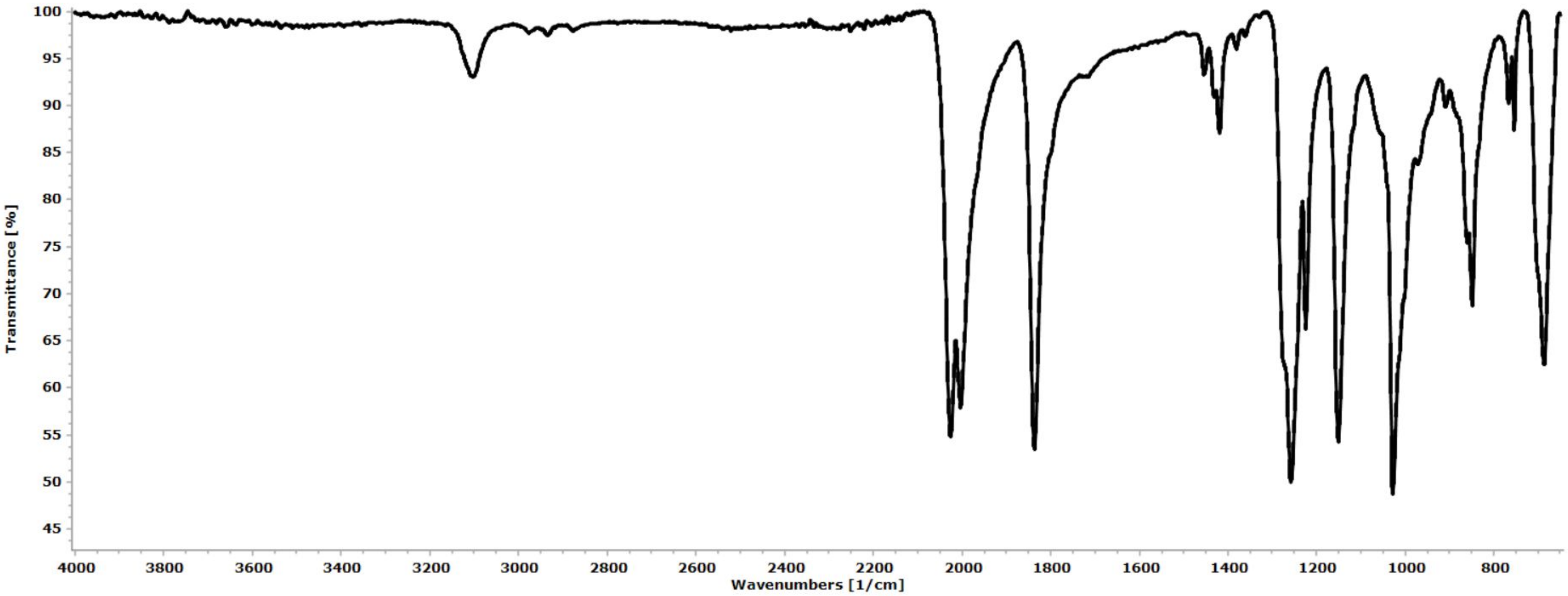

Figure S2. Solid-state IR spectrum (650-4000 cm^{-1}) of $K[Fe_2Cp_2(CO)_3(CNCH_2CO_2)]$, K[**2**]. The intense absorption around 3300 cm^{-1} is due to moisture.

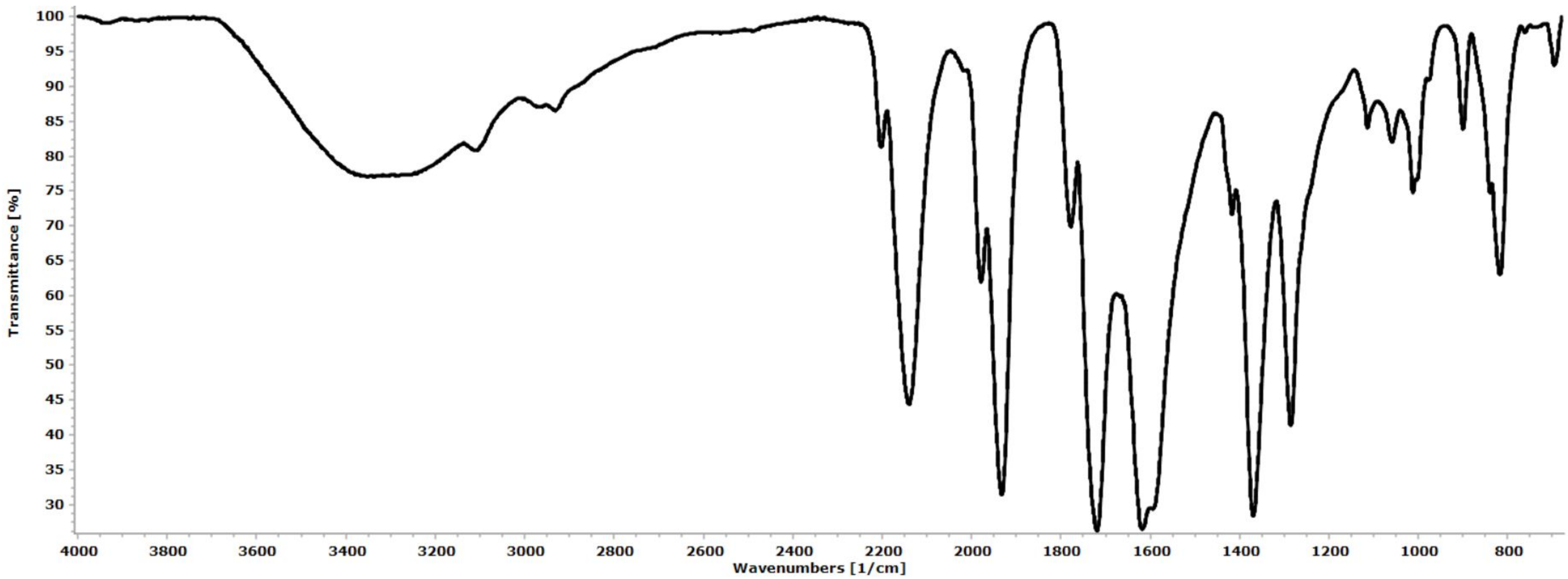

Figure S3. Solid-state IR spectrum (650-4000 cm^{-1}) of $[Fe_2Cp_2(CO)_2(\mu\text{-}CO)(\mu\text{-}CNMe_2)]^+$, **3**$^+$, as NO_3^- (black line) or $CF_3SO_3^-$ (blue line) salt.

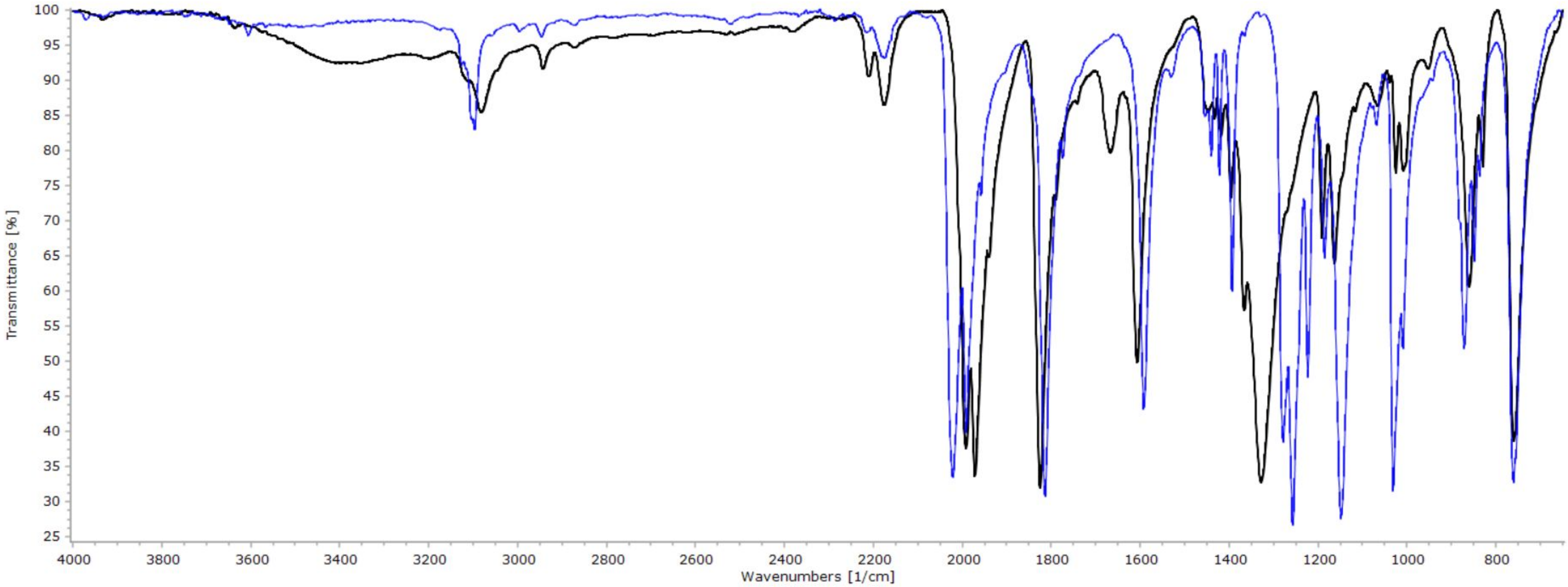

Figure S4. Solid-state IR spectrum (650-4000 cm^{-1}) of [Fe_2Cp_2(CO)(PTA)(μ-CO){μ-CNMeXyl}]CF_3SO_3, [**4**]CF_3SO_3 (*Z*/*E* ratio *ca*. 10:1).

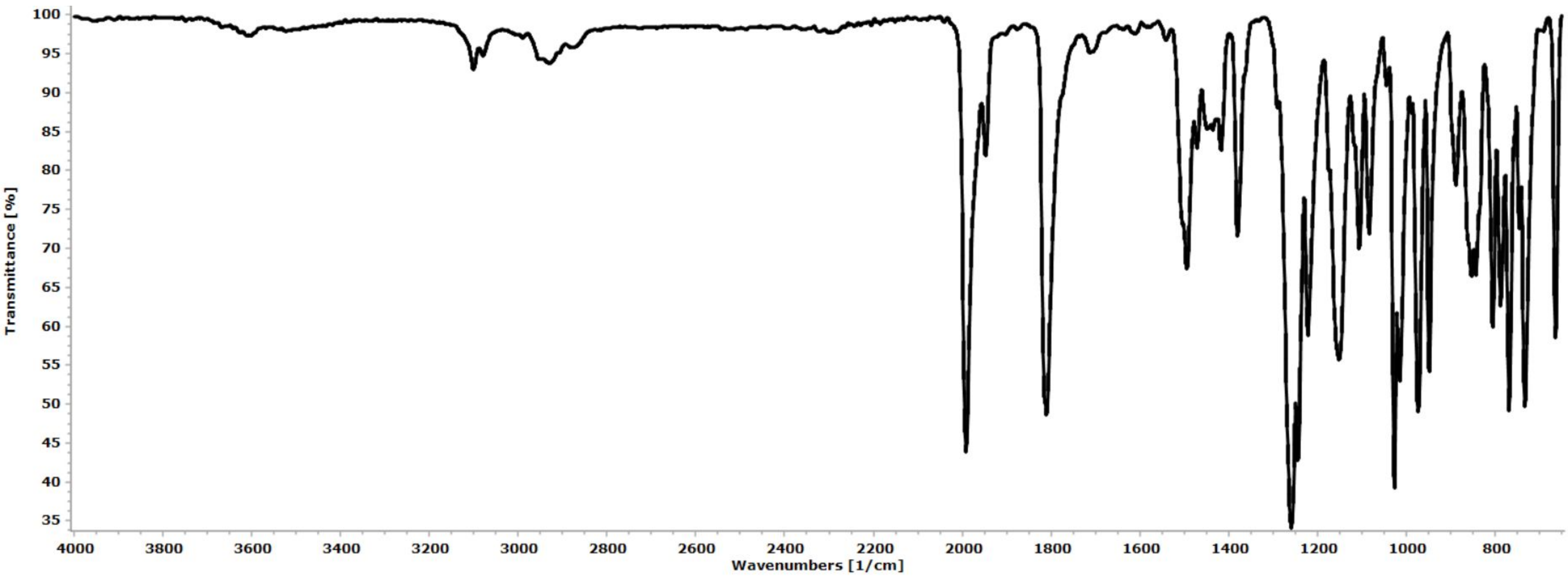

Figure S5. Solid-state IR spectrum (650-4000 cm^{-1}) of [Fe_2Cp_2(CO)(DMSO)(μ-CO)(μ-CNMe$_2$)]CF_3SO_3, [**6**]CF_3SO_3.

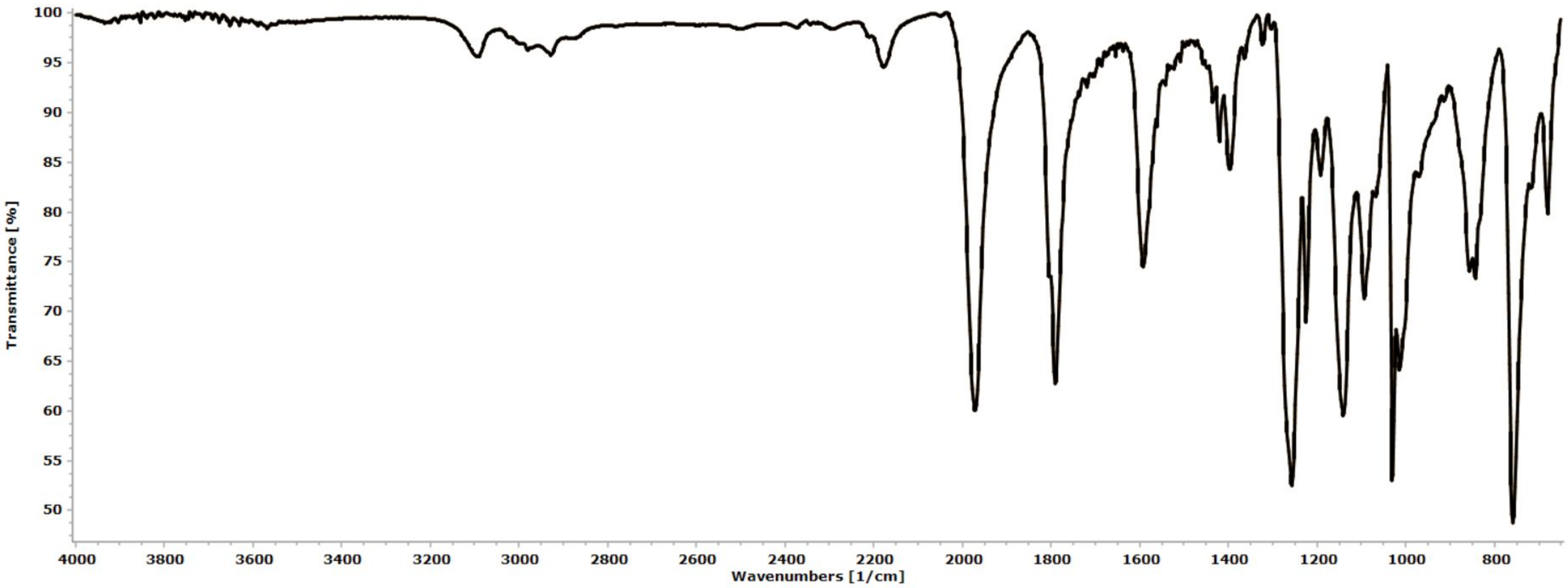

NMR spectra in organic solvents

Figure S6. ^{1}H NMR spectrum (401 MHz, acetone-d_6) of $[Fe_2Cp_2(CO)_2(\mu\text{-}CO)(\mu\text{-}CSEt)]CF_3SO_3$, [**1**]$CF_3SO_3$.

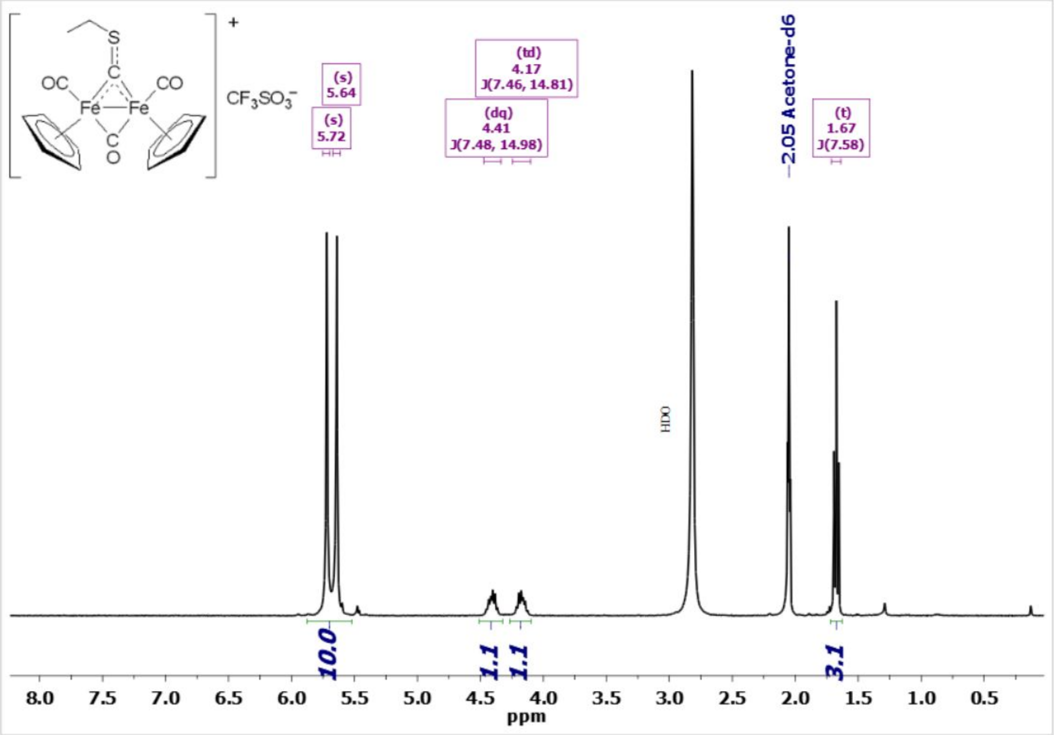

Figure S7. ^{13}C{^{1}H} NMR spectrum (101 MHz, acetone-d_6) of $[Fe_2Cp_2(CO)_2(\mu\text{-}CO)(\mu\text{-}CSEt)]CF_3SO_3$, [**1**]$CF_3SO_3$.

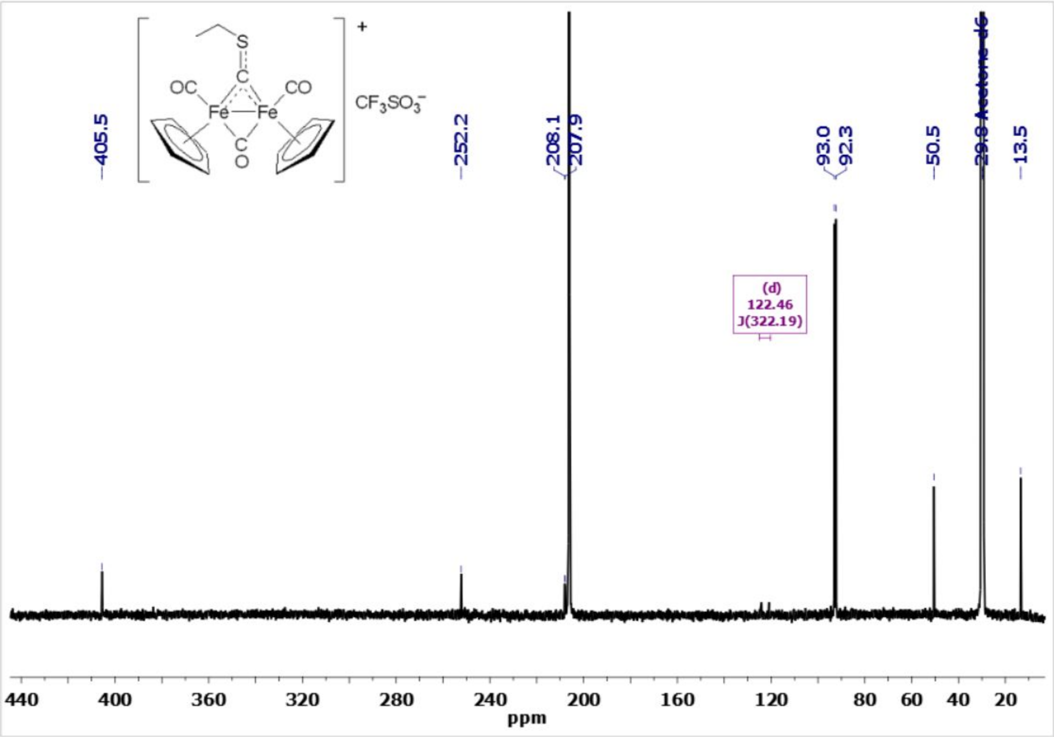

Figure S8. ^{1}H NMR spectrum (401 MHz, CD_3OD) of $K[Fe_2Cp_2(CO)_3(CNCH_2CO_2)]$, K[**2**].

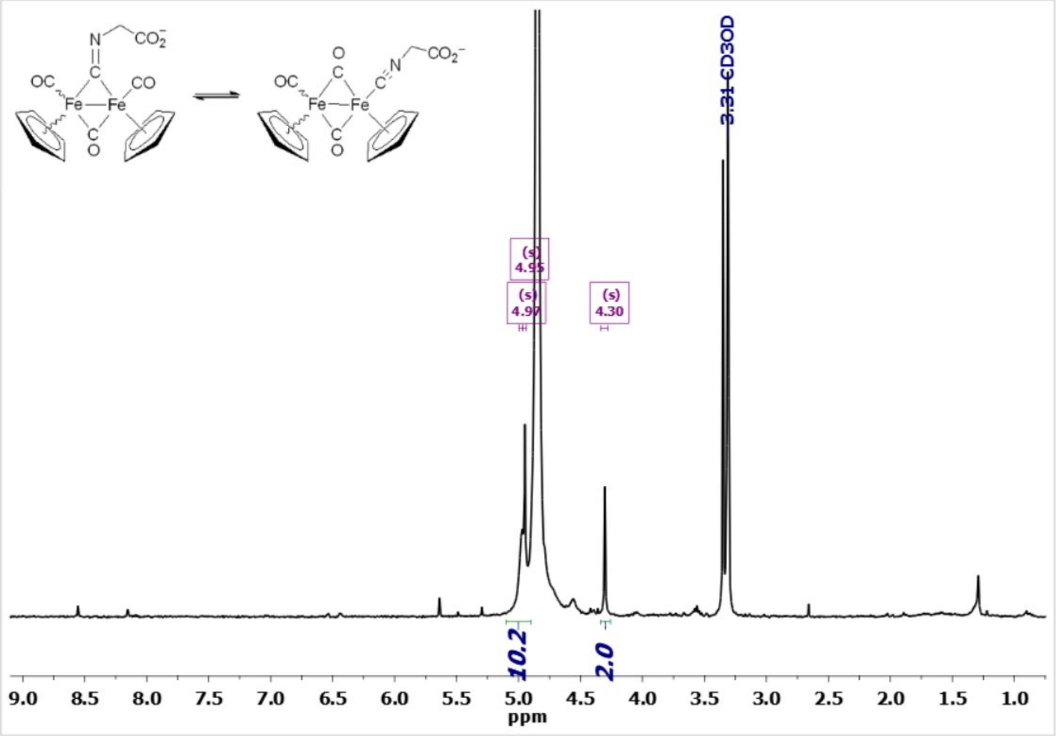

Figure S9. $^{13}C\{^{1}H\}$ NMR spectrum (101 MHz, DMSO-d_6) of $K[Fe_2Cp_2(CO)_3(CNCH_2CO_2)]$, K[**2**].

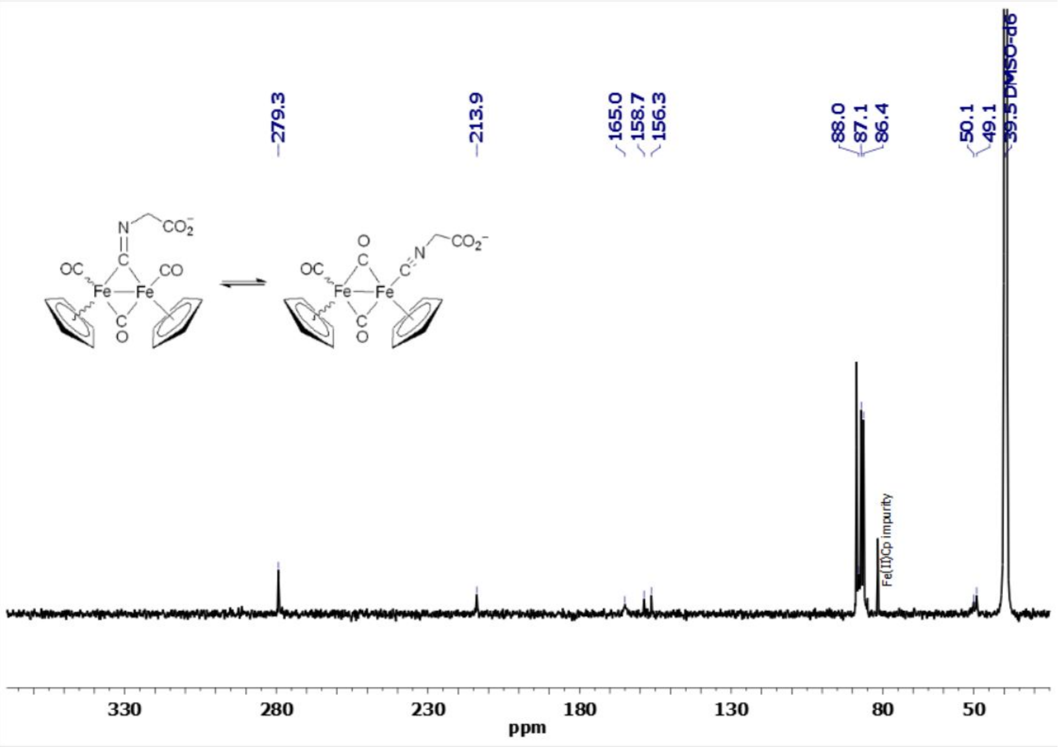

Figure S10. ^{1}H NMR spectrum (401 MHz, CD_3OD) of $[Fe_2Cp_2(CO)_2(\mu\text{-}CO)(\mu\text{-}CNMe_2)]NO_3$, [**3**]$NO_3$. Inexact integral is due to a longer relaxation time of the Cp ligands.

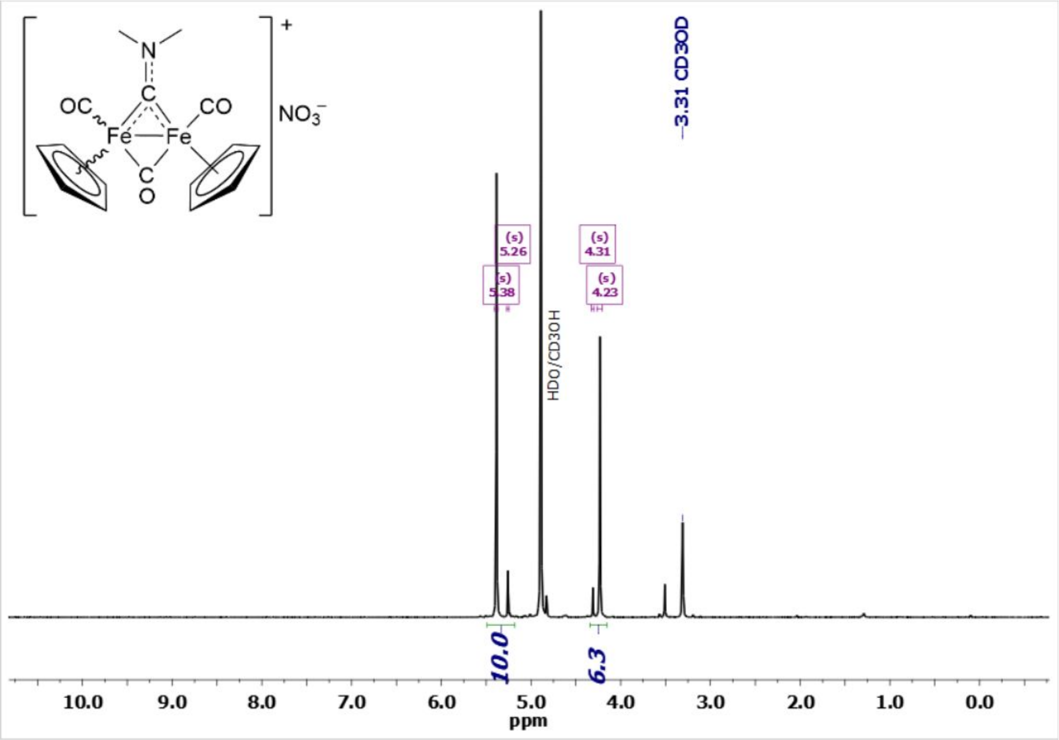

Figure S11. ^{1}H NMR spectrum (401 MHz, acetone-d_6) of $[Fe_2Cp_2(CO)(PTA)(\mu\text{-}CO)(\mu\text{-}CNMeXyl)]CF_3SO_3$, [**4**]$CF_3SO_3$ (*Z*/*E* ratio *ca*. 10).

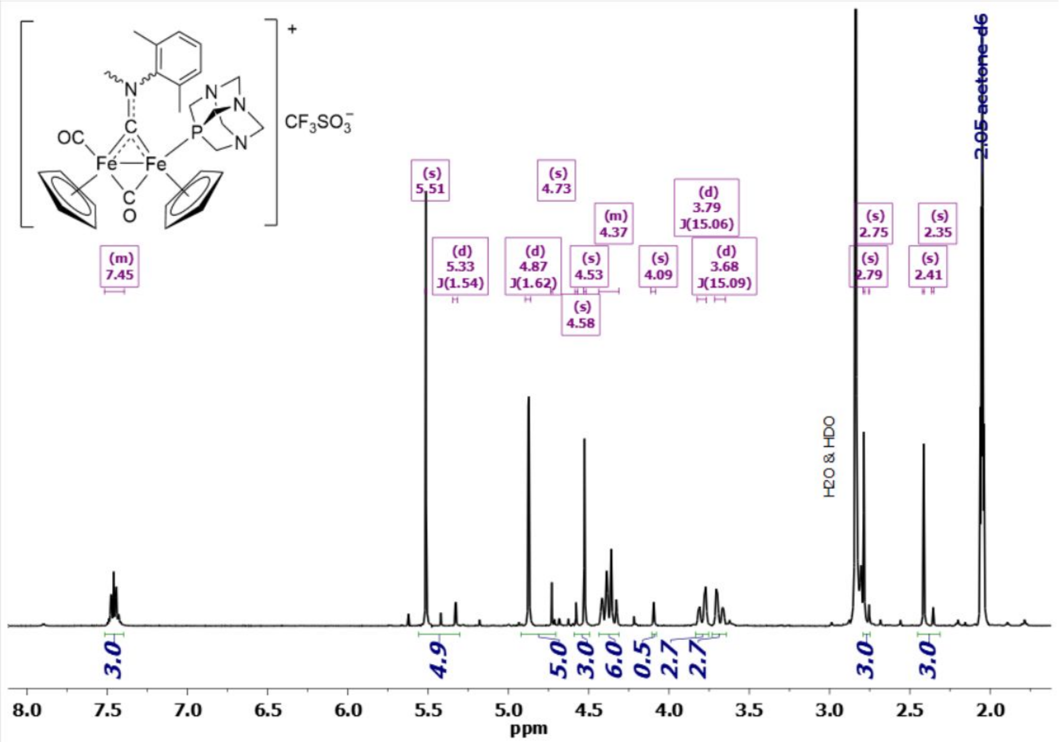

Figure S12. $^{13}C\{^1H\}$ NMR spectrum (101 MHz, acetone-d$_6$) of $[Fe_2Cp_2(CO)(PTA)(\mu\text{-}CO)(\mu\text{-}CNMeXyl)]CF_3SO_3$, [**4**]$CF_3SO_3$. Signals at 210 and 23 ppm are due to an impurity of acetone-d$_6$.

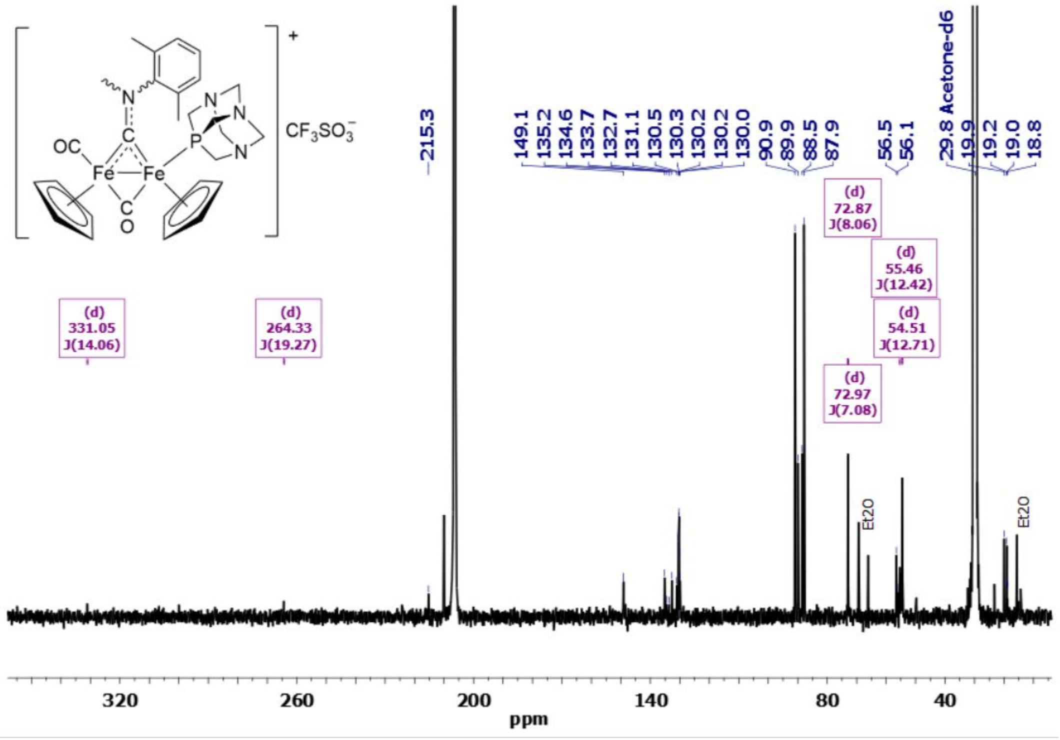

Figure S13. $^{31}P\{^1H\}$ NMR spectrum (162 MHz, acetone-d$_6$) of $[Fe_2Cp_2(CO)(PTA)(\mu\text{-}CO)(\mu\text{-}CNMeXyl)]CF_3SO_3$, [**4**]$CF_3SO_3$ (*Z*/*E* ratio *ca*. 10).

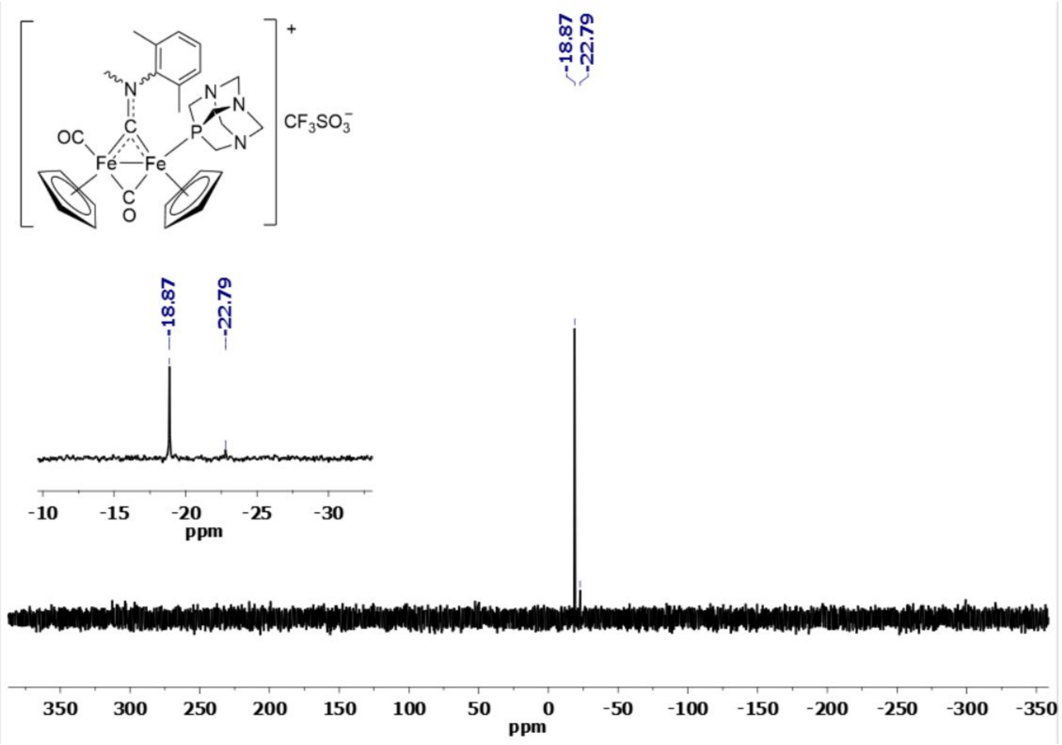

Figure S14. ^{1}H (black line) and ^{1}H-NOE (red line: irradiation at 5.51 ppm; blue line: irradiation at 4.87 ppm) NMR spectra (401 MHz, acetone-d$_6$) of [$Fe_2Cp_2(CO)(PTA)(\mu\text{-}CO)(\mu\text{-}CNMeXyl)$]$CF_3SO_3$, [**4**]$CF_3SO_3$ (*Z* isomer).

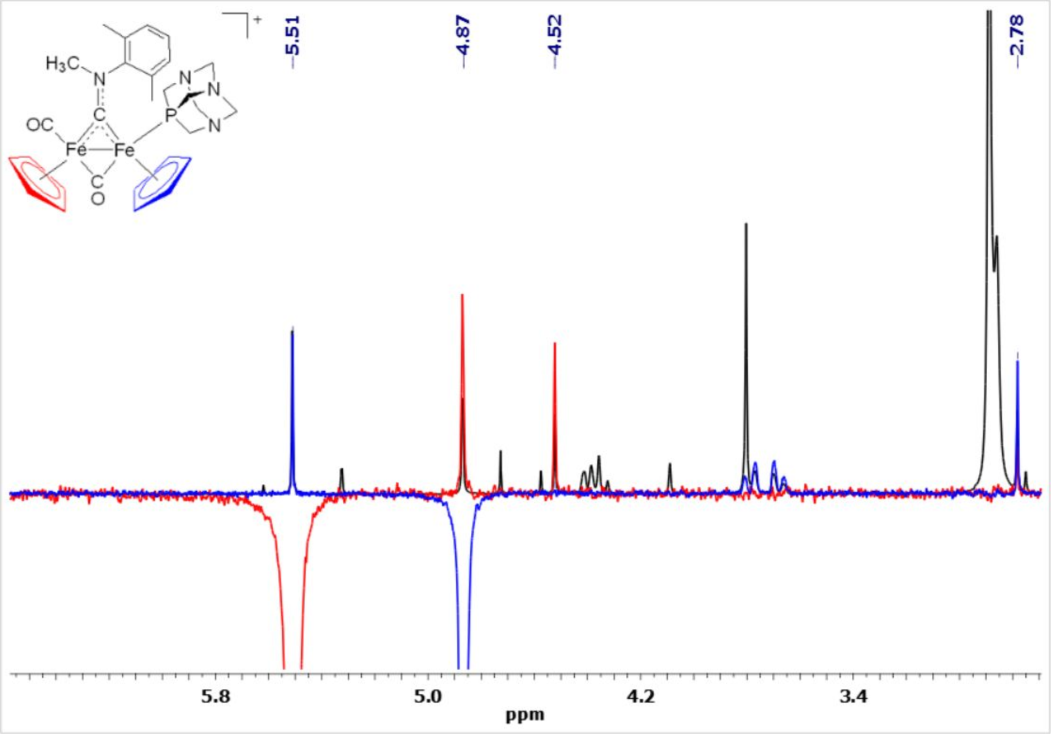

Figure S15. ^{1}H (black line) and ^{1}H-NOE (blue line: irradiation at 5.33 ppm; red line: irradiation at 4.73 ppm) NMR spectra (401 MHz, acetone-d$_6$) of [$Fe_2Cp_2(CO)(PTA)(\mu\text{-}CO)(\mu\text{-}CNMeXyl)$]$CF_3SO_3$, [**4**]$CF_3SO_3$ (*E* isomer).

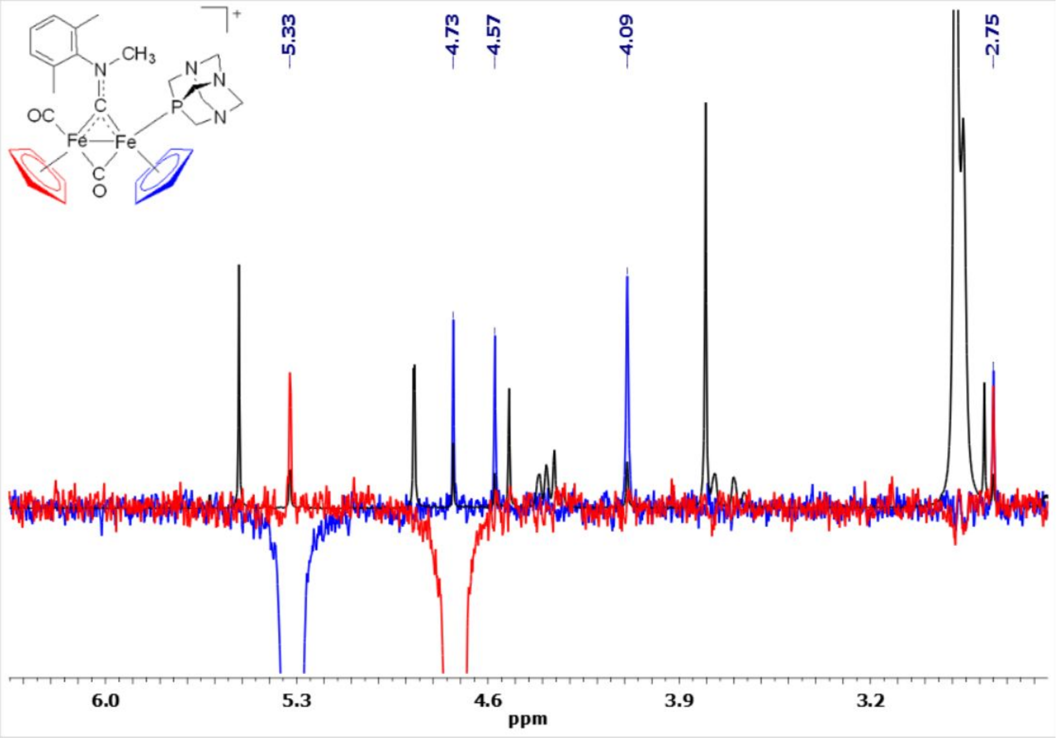

Figure S16. ^{1}H NMR spectrum (401 MHz, acetone-d_6) of $[Fe_2Cp_2(CO)(DMSO)(\mu\text{-}CO)(\mu\text{-}CNMe_2)]CF_3SO_3$, [**6**]$CF_3SO_3$. Integrals refer to signals of the major (*cis*) isomer.

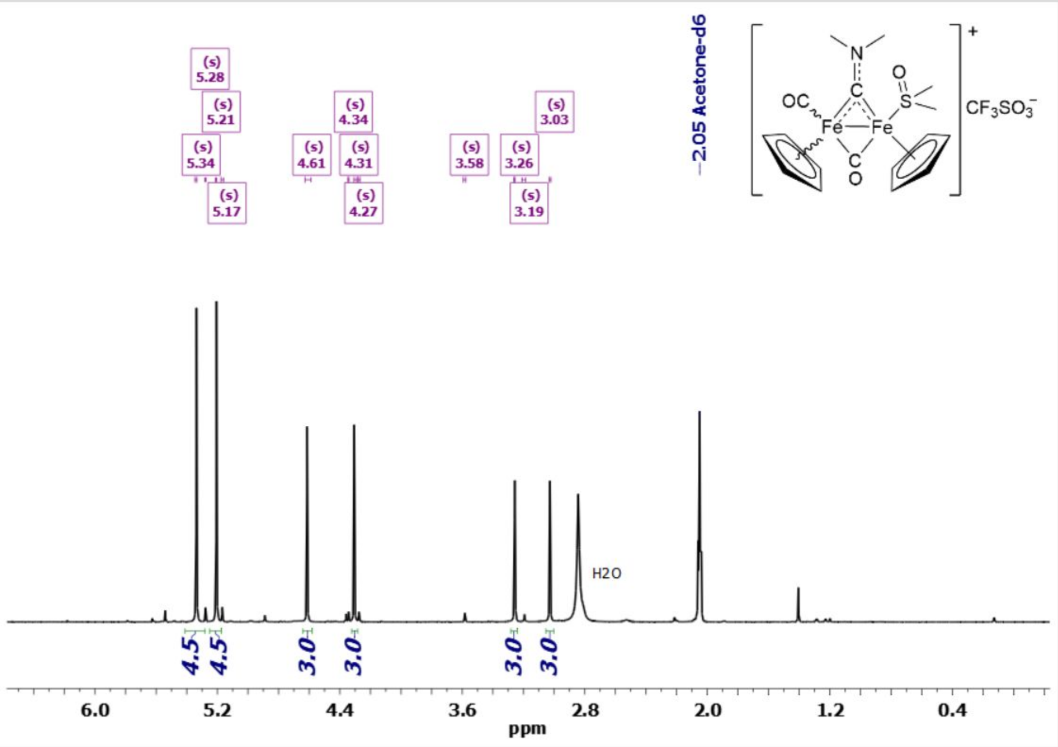

Figure S17. $^{13}C\{^{1}H\}$ NMR spectrum (101 MHz, acetone-d_6) of $[Fe_2Cp_2(CO)(DMSO)(\mu\text{-}CO)(\mu\text{-}CNMe_2)]CF_3SO_3$, [**6**]$CF_3SO_3$.

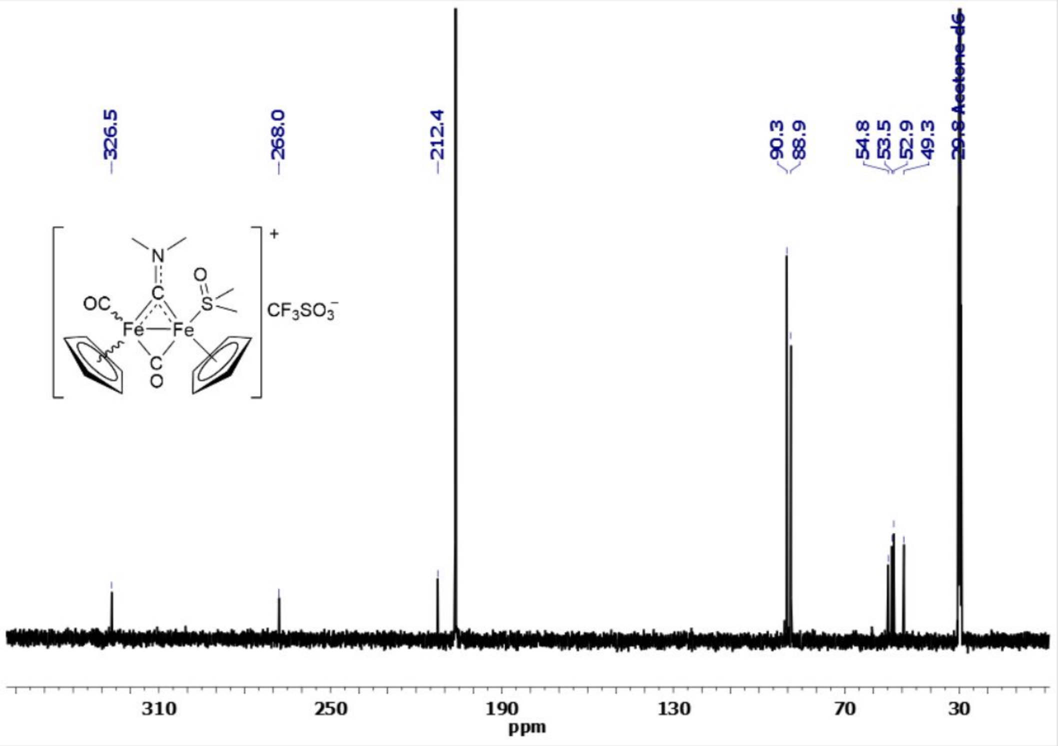

^{1}H, ^{13}C NMR and UV-Vis spectra in water (D_2O)

Figure S18. ^{1}H NMR spectrum (401 MHz, D_2O) of $[Fe_2Cp_2(CO)_2(\mu\text{-}CO)(\mu\text{-}CSEt)]CF_3SO_3$, [**1**]$CF_3SO_3$.

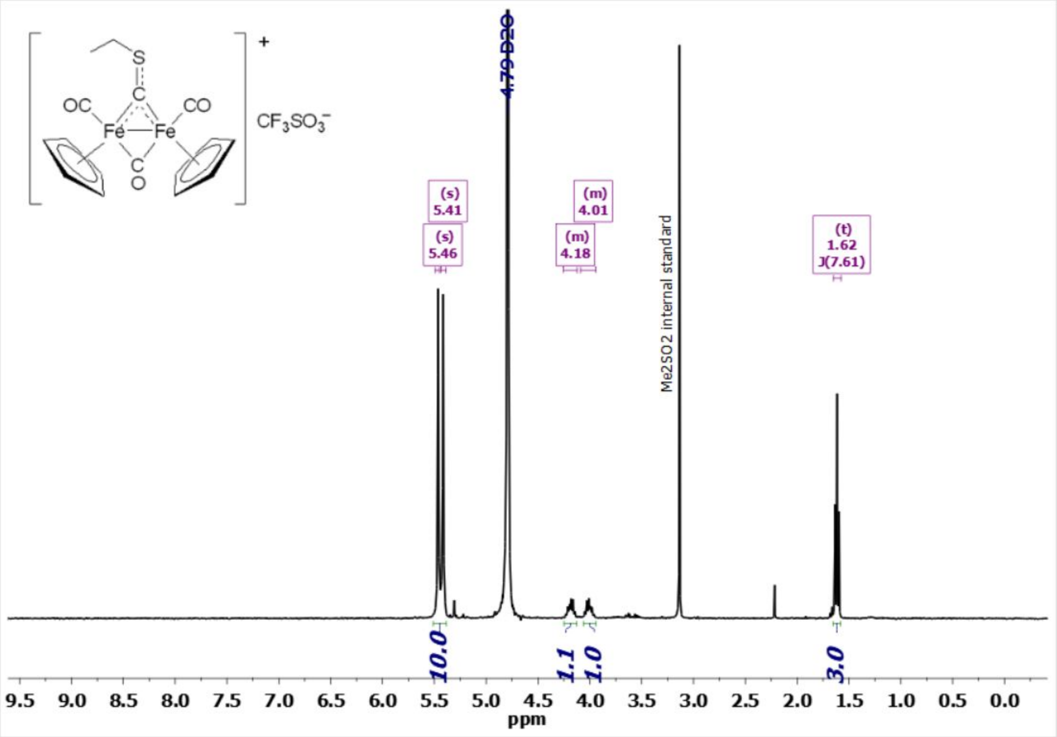

Figure S19. ^{1}H NMR spectrum (401 MHz, D_2O) of $K[Fe_2Cp_2(CO)_3(CNCH_2CO_2)]$, K[**2**].

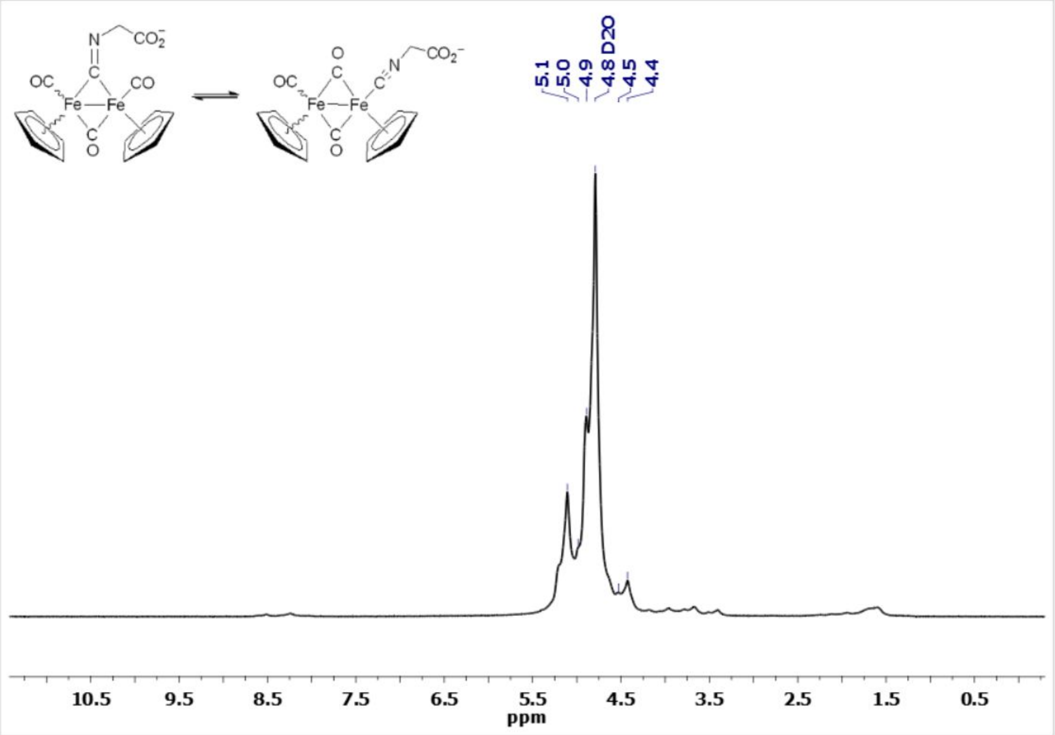

Figure S20. ^{1}H NMR spectrum (401 MHz, D_2O) of $[Fe_2Cp_2(CO)_2(\mu\text{-}CO)(\mu\text{-}CNMe_2)]NO_3$, [**3**]$NO_3$. Inexact integral is due to a longer relaxation time of the Cp ligands.

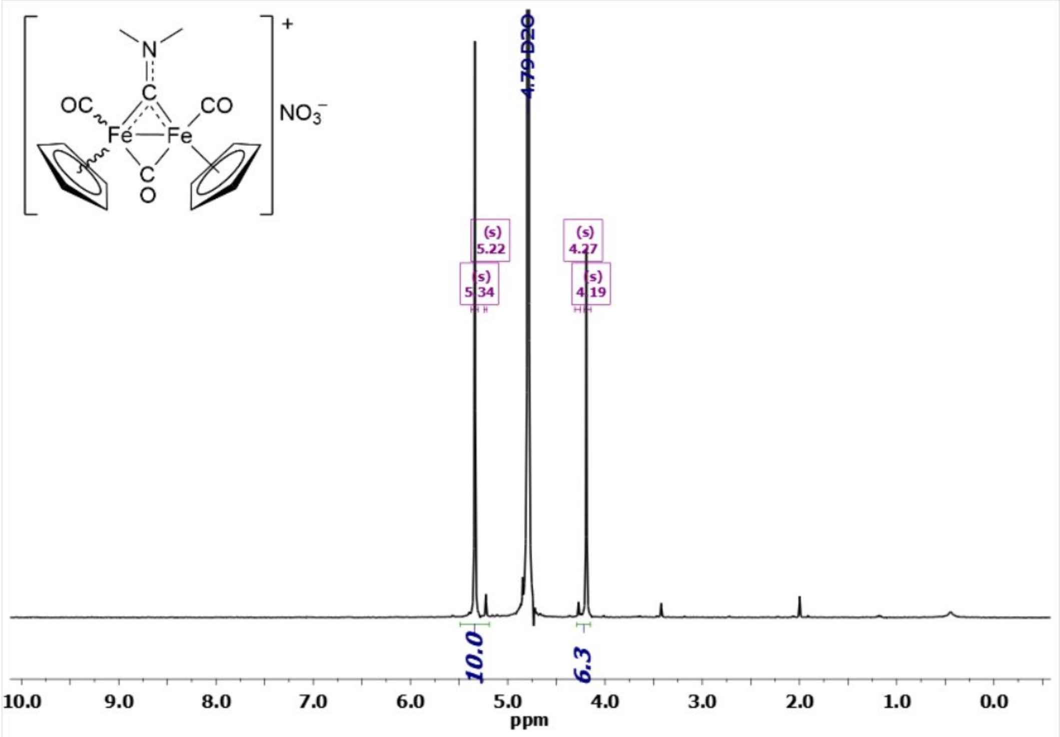

Figure S21. ^{1}H NMR spectrum (401 MHz, D_2O) of $[Fe_2Cp_2(CO)(PTA)(\mu\text{-}CO)(\mu\text{-}CNMeXyl)]CF_3SO_3$, [**4**]$CF_3SO_3$.

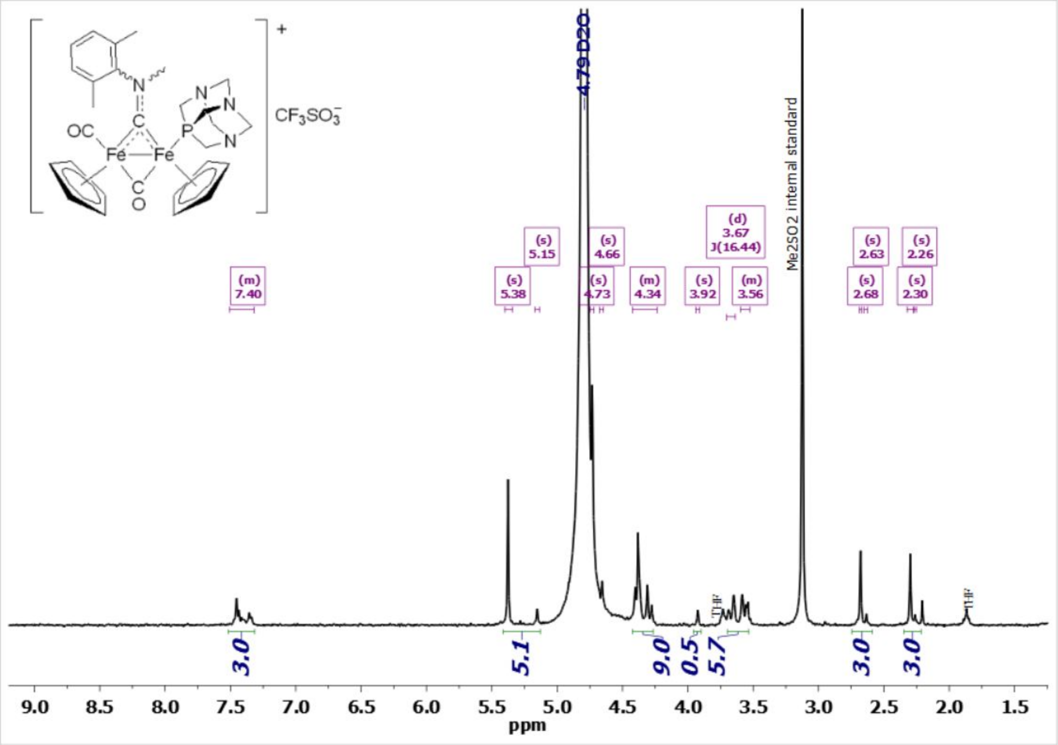

Figure S22. ^{1}H NMR spectrum (401 MHz, D_2O) of $[Fe_2Cp_2(CO)_2(\mu\text{-}CO)\{\mu\text{-}\eta^1:\eta^3\text{-}C(4\text{-}C_6H_4CO_2H)CHCNMe_2\}]CF_3SO_3$, [**5**]$CF_3SO_3$.

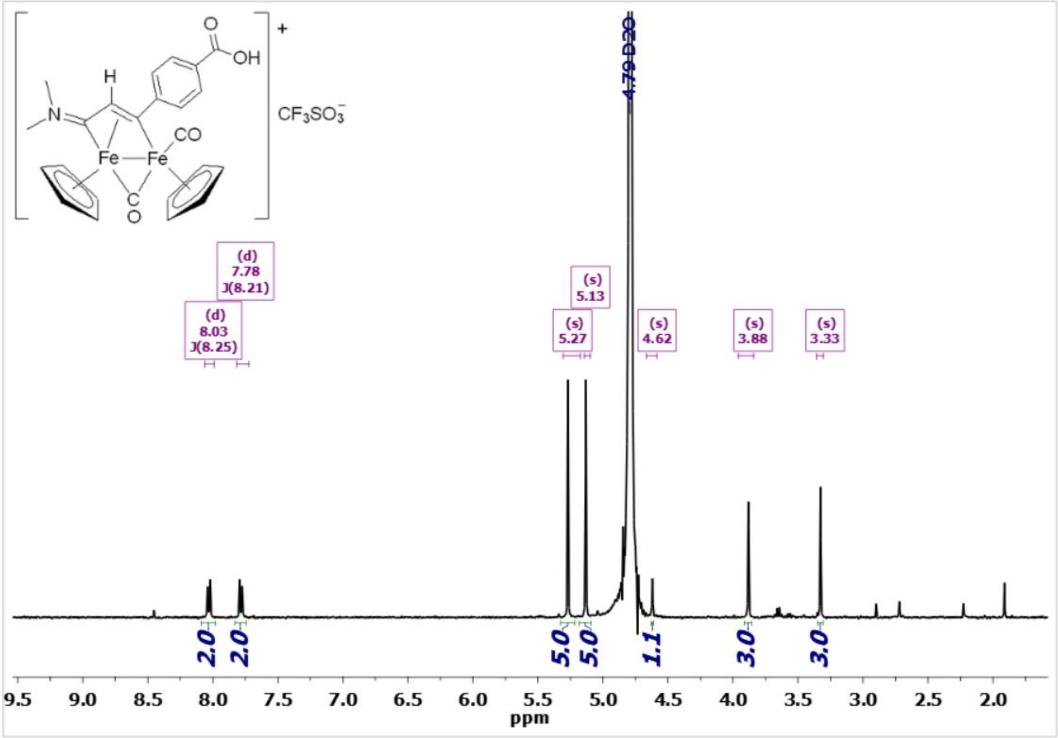

Figure S23. ^{13}C{^{1}H} NMR spectrum (101 MHz, D_2O) of $K[Fe_2Cp_2(CO)_3(CNCH_2CO_2)]$, K[**2**].

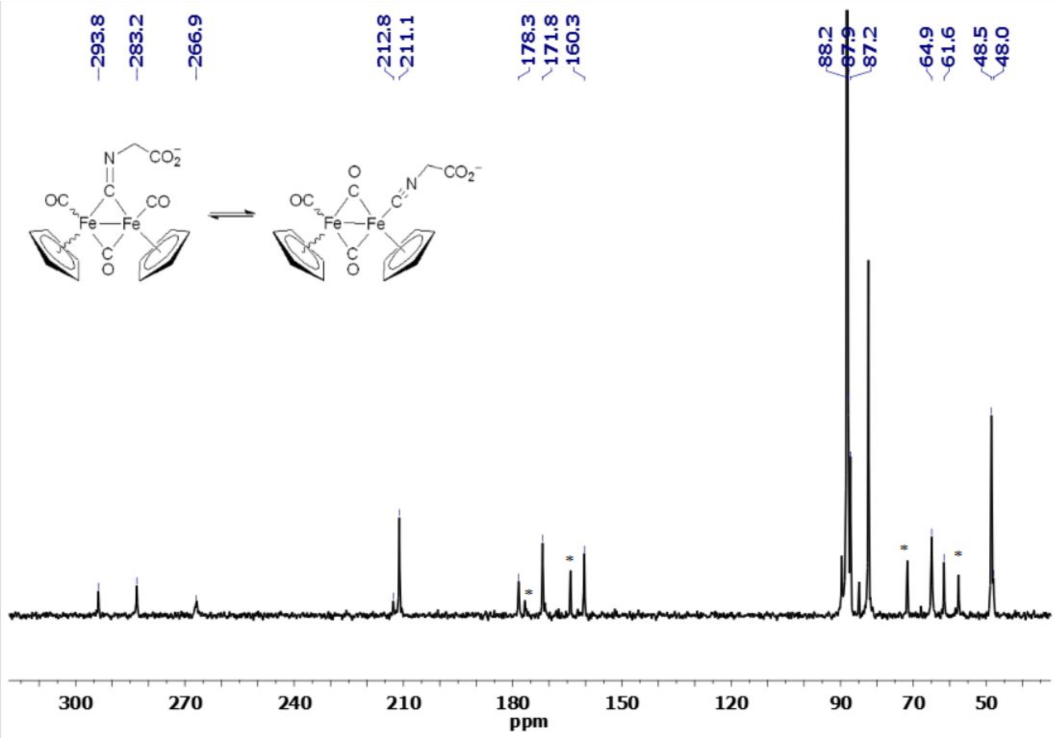

Figure S24. UV-Vis spectra (240-750 cm^{-1}) of diiron complexes in aqueous solution; concentration range: $3.7 \cdot 10^{-4}$ – $1.9 \cdot 10^{-3}$ $mol \cdot L^{-1}$.

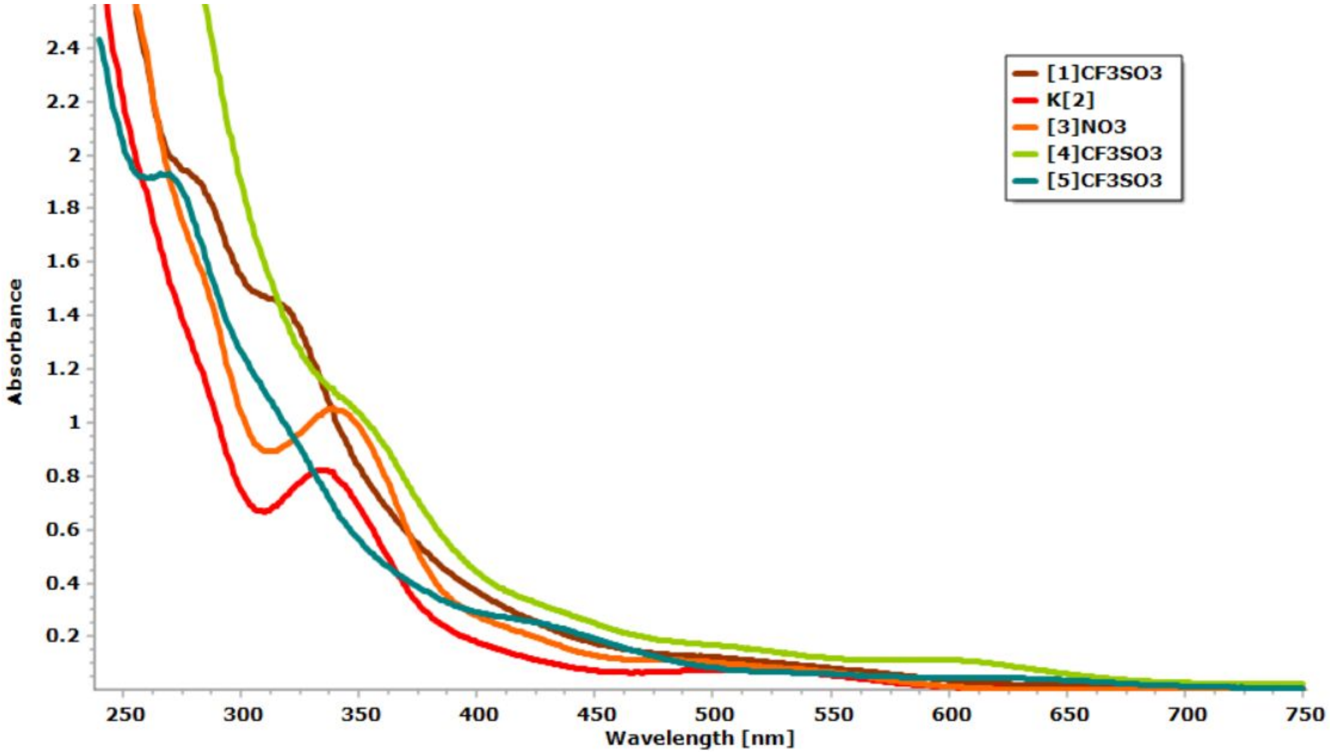

^{13}C NMR, IR and UV-vis spectra upon UV irradiation

Figure S25. $^{13}C\{^{1}H\}$ NMR spectra of [**3**]NO_3 in D_2O after different periods of exposure to 350 nm radiation ($E_v \approx 6$ mW/cm^2): 0 h **(a)**, 10 min **(b)**; 60 min **(c)**; 4 h **(d)**.

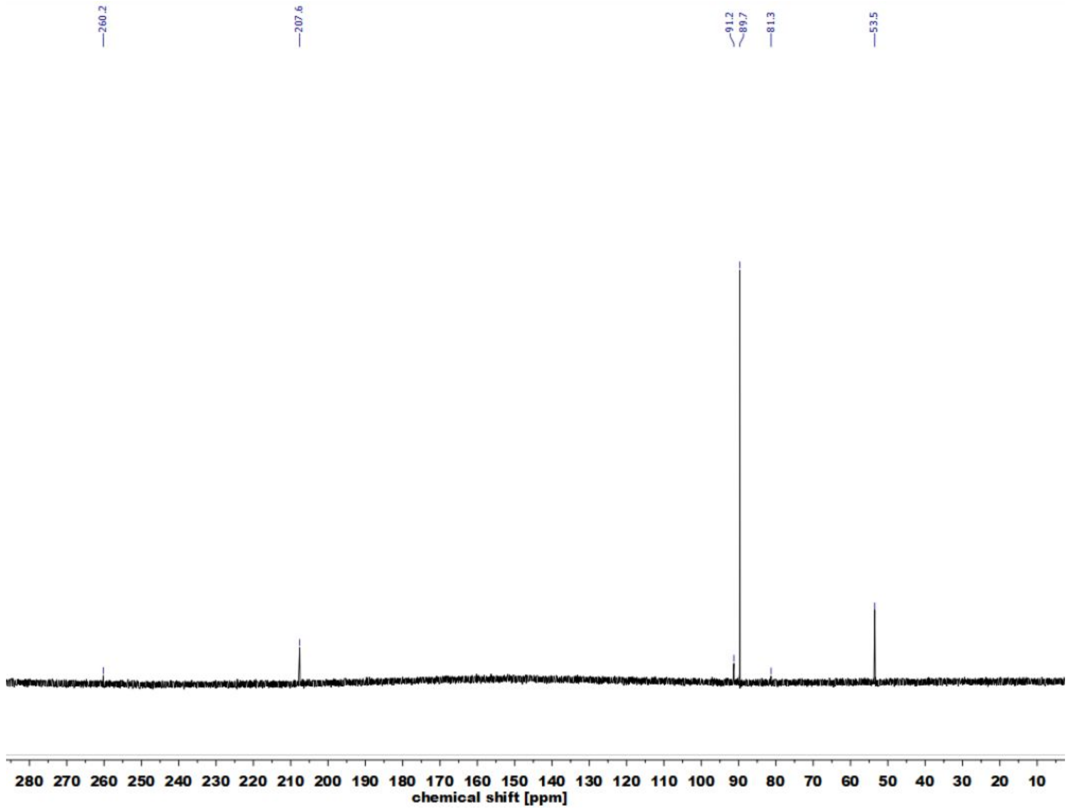

(a)

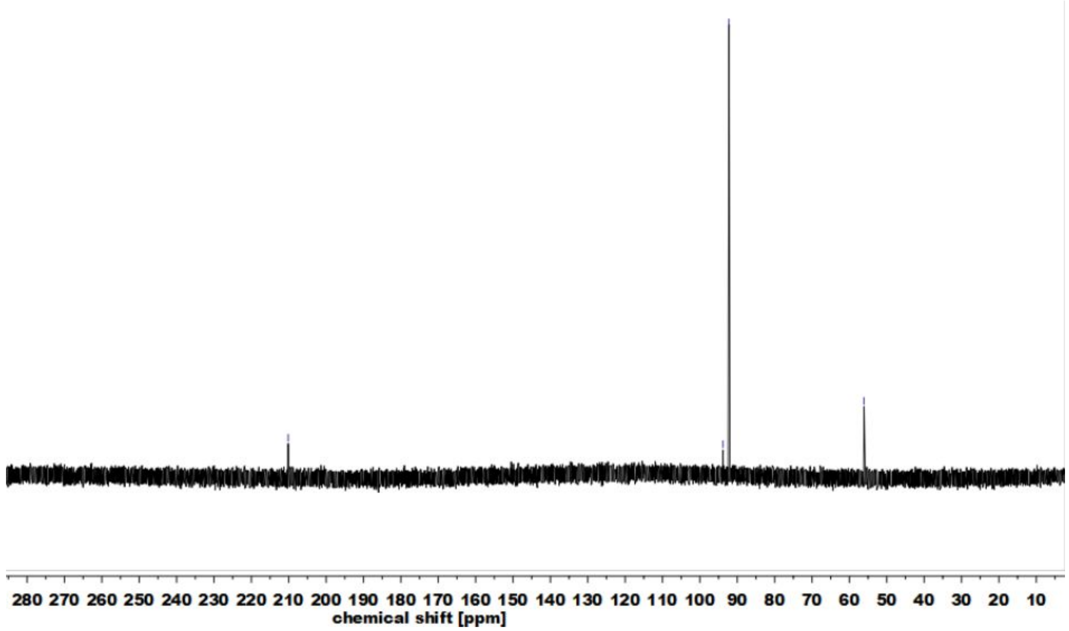

(b)

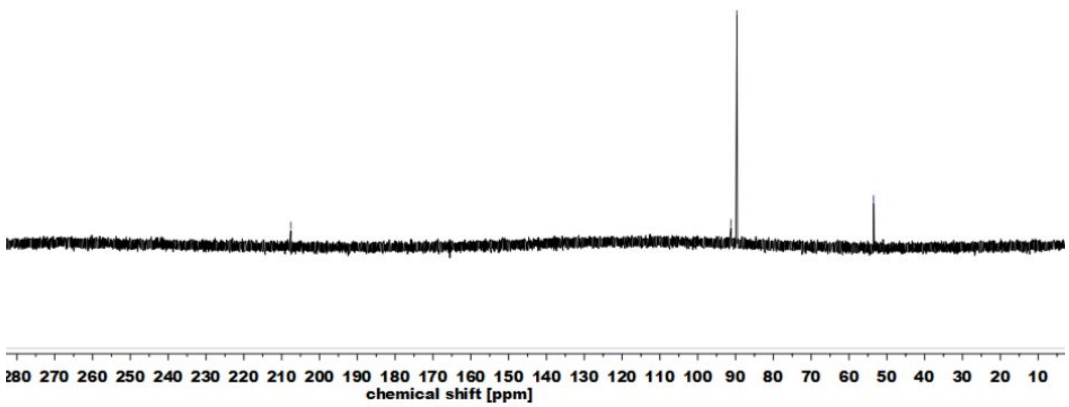

(c)

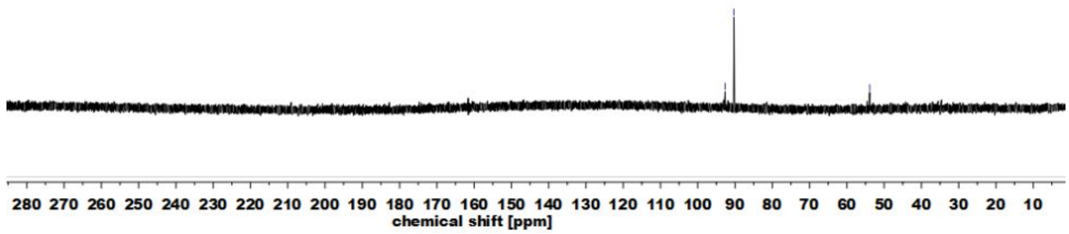

(d)

Figure S26. IR (ATR) spectra measured for [**3**]NO_3 in 0.8% (v/v) DMSO in water after different periods of exposure to 350 nm radiation ($E_v \approx 6$ mW/cm^2) at 37 °C and volatiles removal under vacuum.

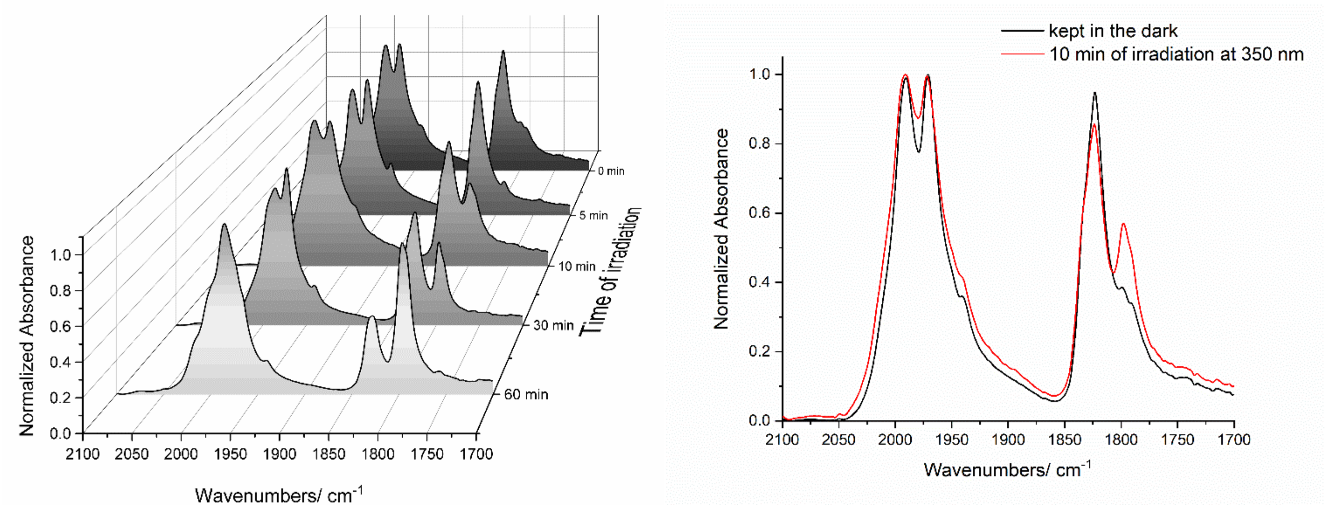

Figure S27. IR (ATR) spectra measured for [**1**]CF_3SO_3 in 0.8% (v/v) DMSO in water after different periods of exposure to 350 nm radiation (E_v ≈ 6 mW/cm^2) at 37 °C and volatiles removal under vacuum.

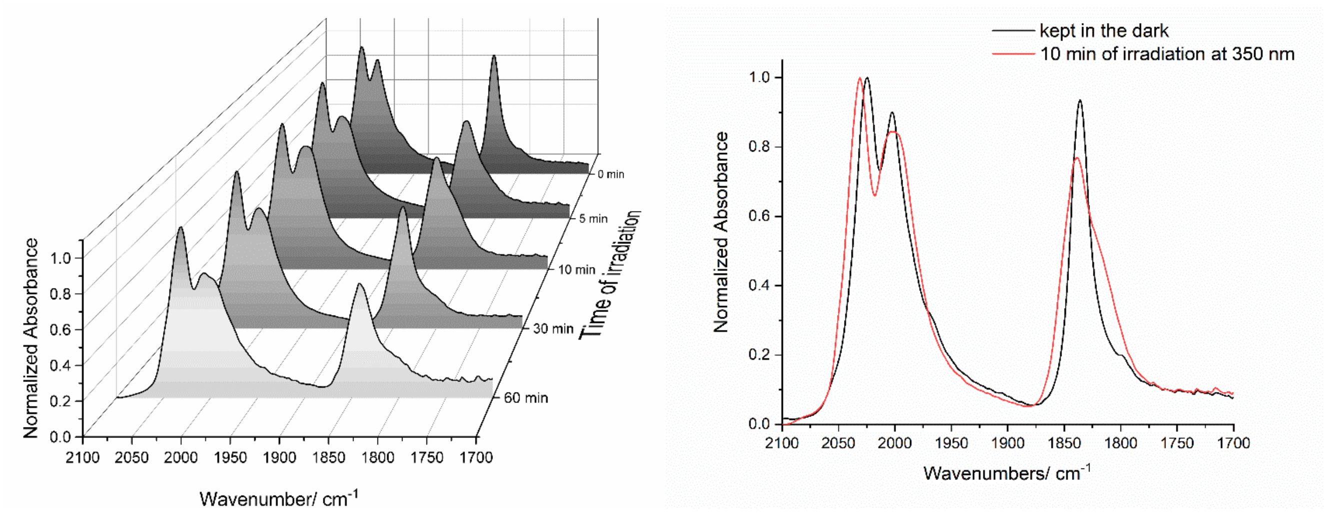

Figure S28. IR (ATR) spectra measured for K[**2**] in H_2O after different periods of exposure to 350 nm radiation (E_v ≈ 6 mW/cm^2) at 37 °C and volatiles removal under vacuum.

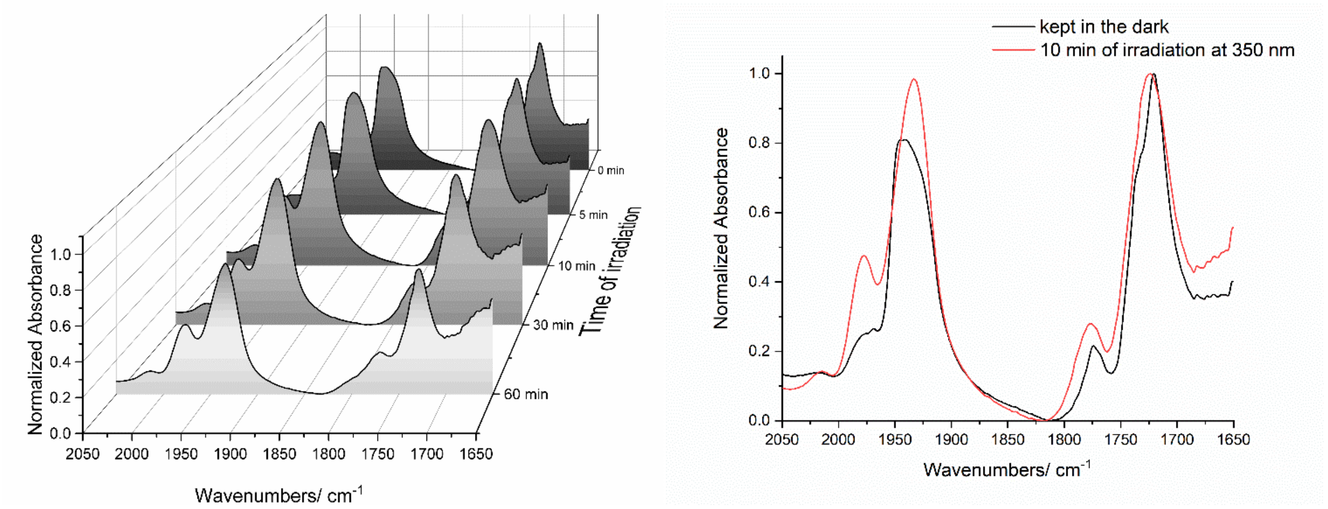

Figure S29. IR (ATR) spectra measured for [**5**]CF_3SO_3 in MeCN after different periods of exposure to 350 nm radiation ($E_v \approx 6$ mW/cm^2) at 37 °C and volatiles removal under vacuum.

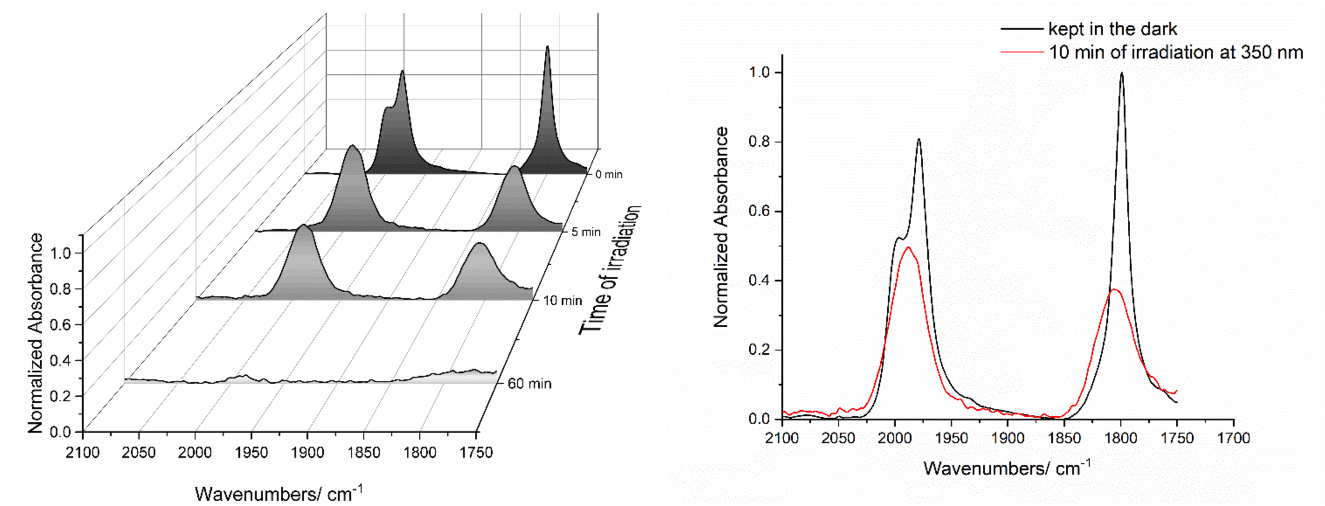

Figure S30. IR (ATR) spectra measured for [**4**]CF_3SO_3 in MeCN after different periods of exposure to 350 nm radiation ($E_v \approx 6$ mW/cm^2) at 37 °C and volatiles removal under vacuum. Differences in the IR relative intensity of the carbonyl stretching bands (1995, 1945 cm^{-1}) are presumably related to *Z* to *E* isomerization.

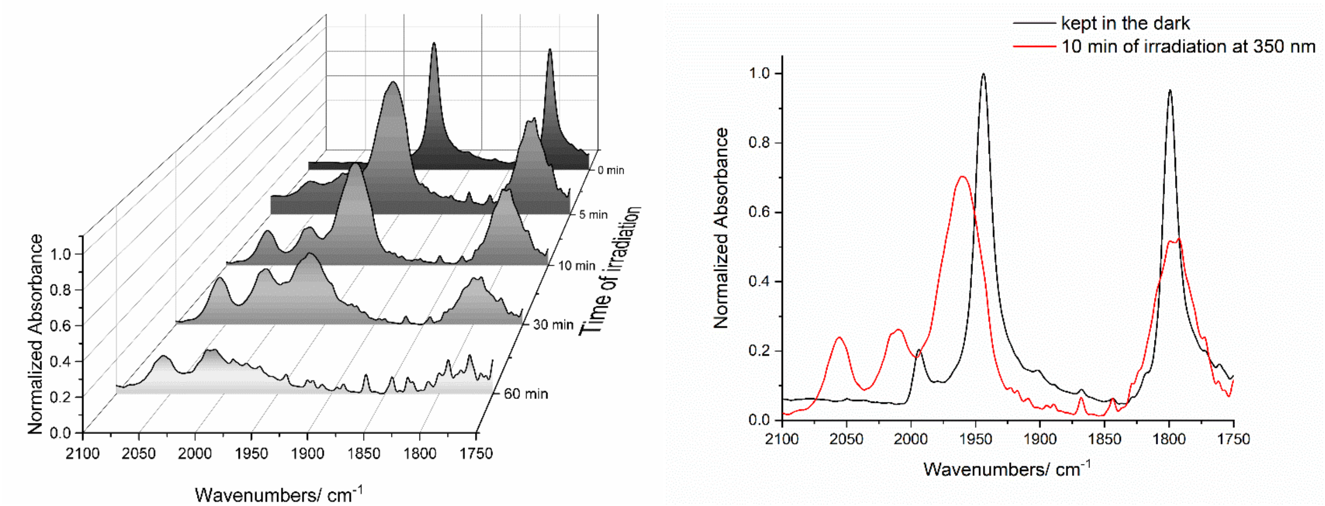

Cell viability experiments

Figure S31. MTS assay of [**1**]CF_3SO_3 (0 - 100 µM) in A431 **(a)** and HEK293 **(b)** cell lines, measured after 24 and 48 h; with or without 10 min exposure to 350 nm radiation (E_v = 6 mW/cm^2) at 37 °C. The percentage of cell viability is expressed relative to untreated cells (ANOVA at α = 0.05).

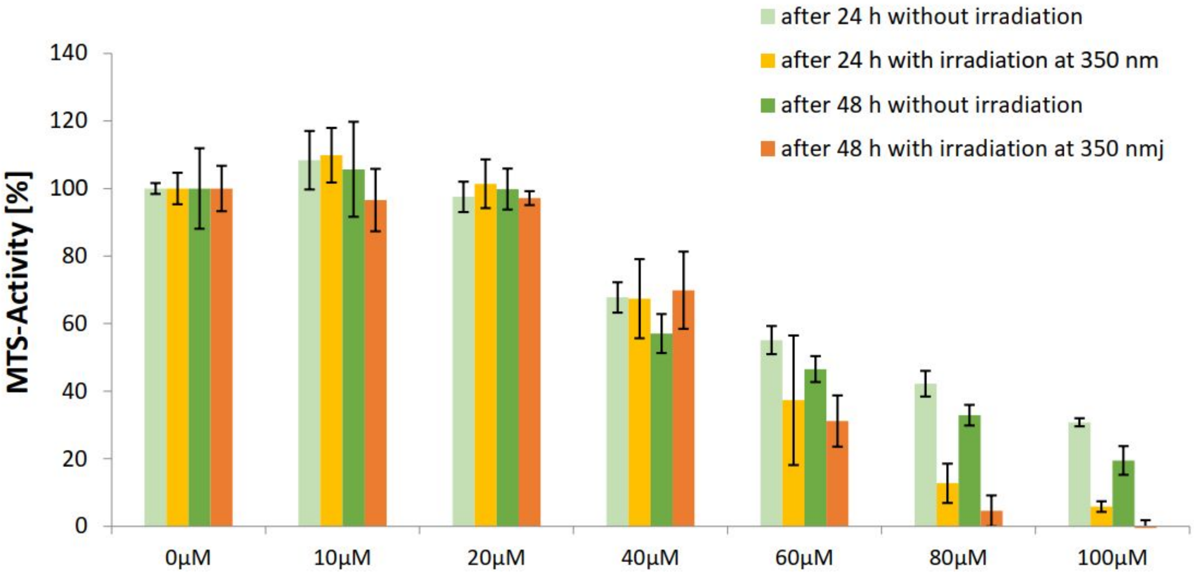

(a)

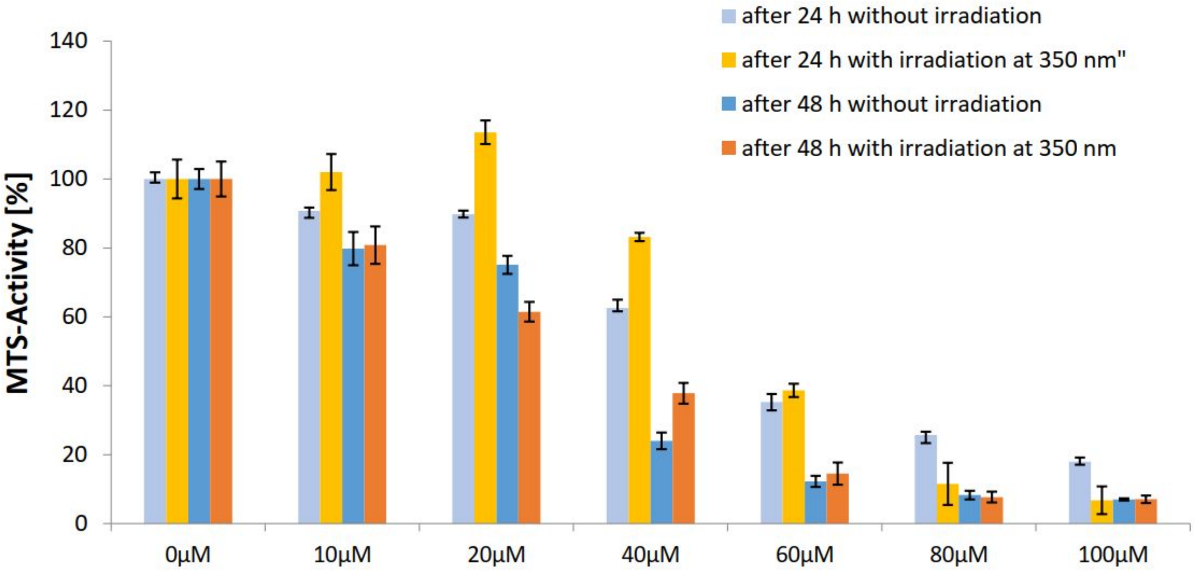

(b)

Figure S32. MTS assay of K[**2**] (0 - 100 µM) in A431 **(a)** and HEK293 **(b)** cell lines, measured after 24 and 48 h; with or without 10 min exposure to 350 nm radiation (E_v = 6 mW/cm^2) at 37 °C. The percentage of cell viability is expressed relative to untreated cells (ANOVA at α = 0.05).

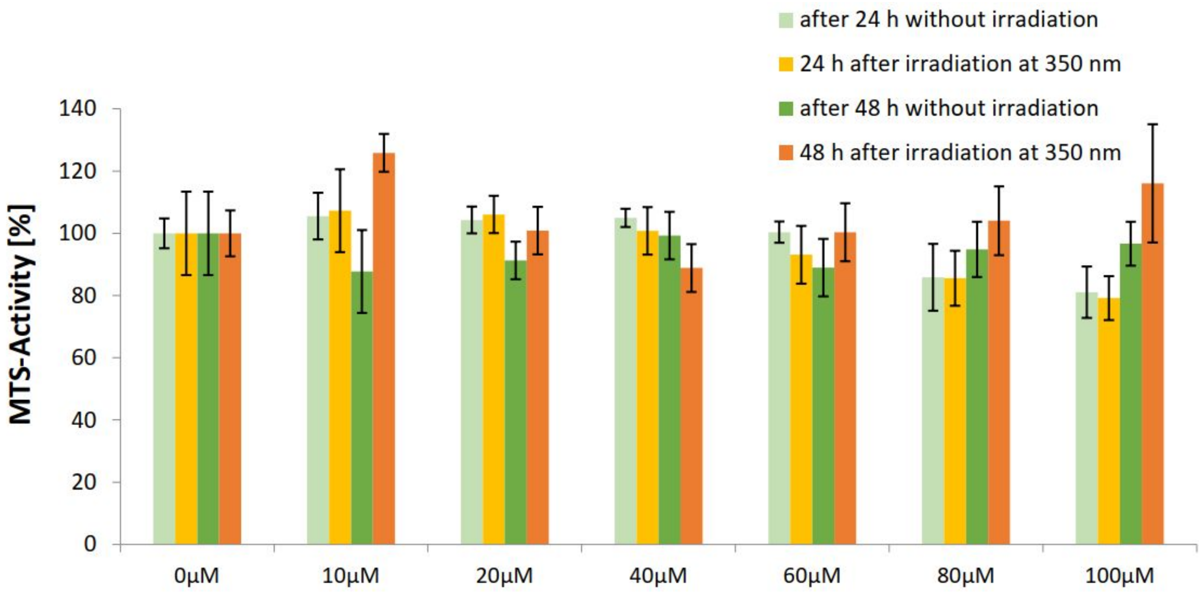

(a)

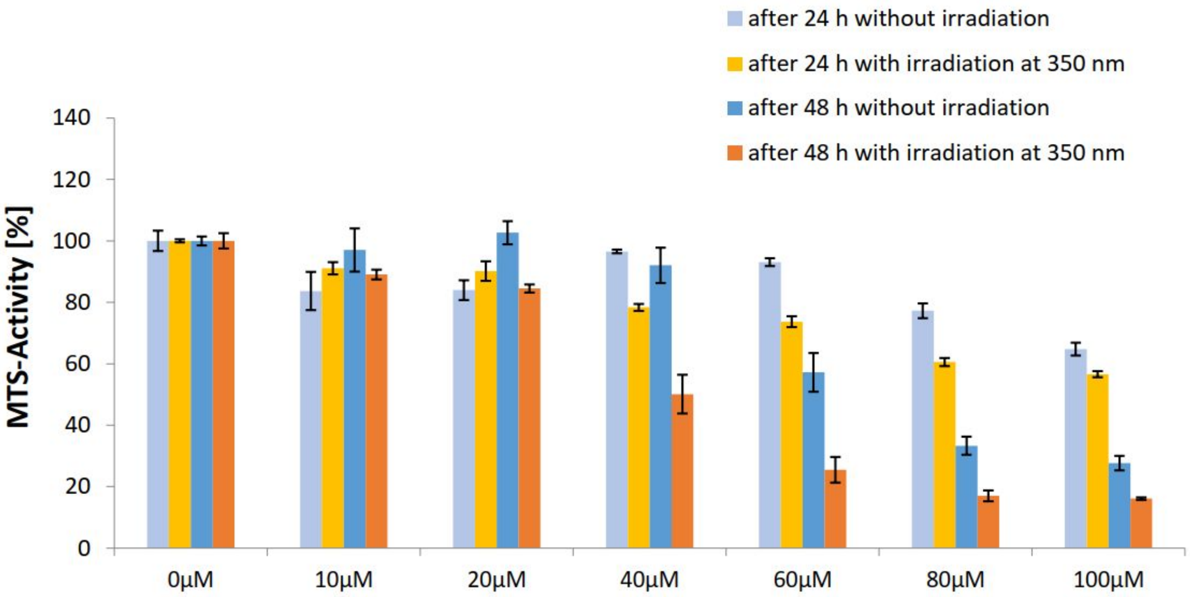

(b)

Figure S33. MTS assay of [**3**]NO_3 (0 - 125 µM) in A431 **(a)** and HEK293 **(b)** cell lines, measured after 24 and 48 h; with or without 10 min exposure to 350 nm radiation (E_v = 6 mW/cm^2) at 37 °C. The percentage of cell viability is expressed relative to untreated cells (ANOVA at α = 0.05).

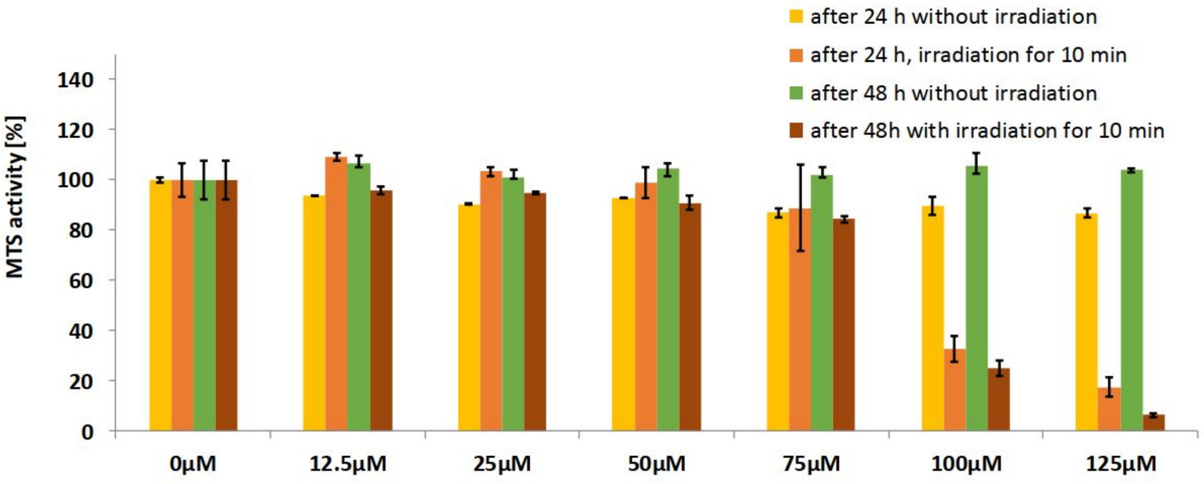

(a)

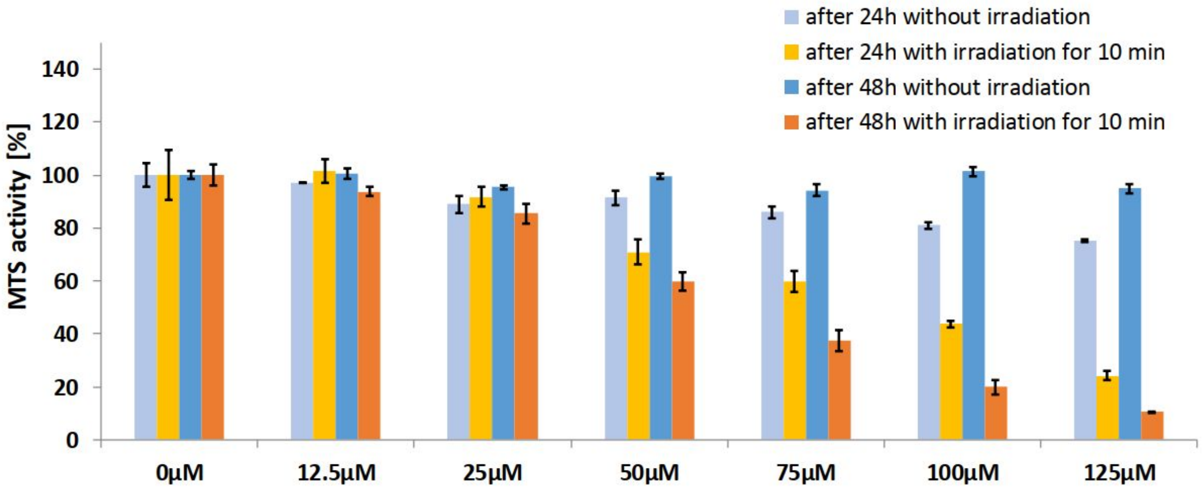

(b)

Figure S34. MTS assay of [**4**]CF_3SO_3 (0 - 100 µM) in A431 **(a)** and HEK293 **(b)** cell lines, measured after 24 and 48 h; with or without 10 min exposure to 350 nm radiation (E_v = 6 mW/cm^2) at 37 °C. The percentage of cell viability is expressed relative to untreated cells (ANOVA at α = 0.05).

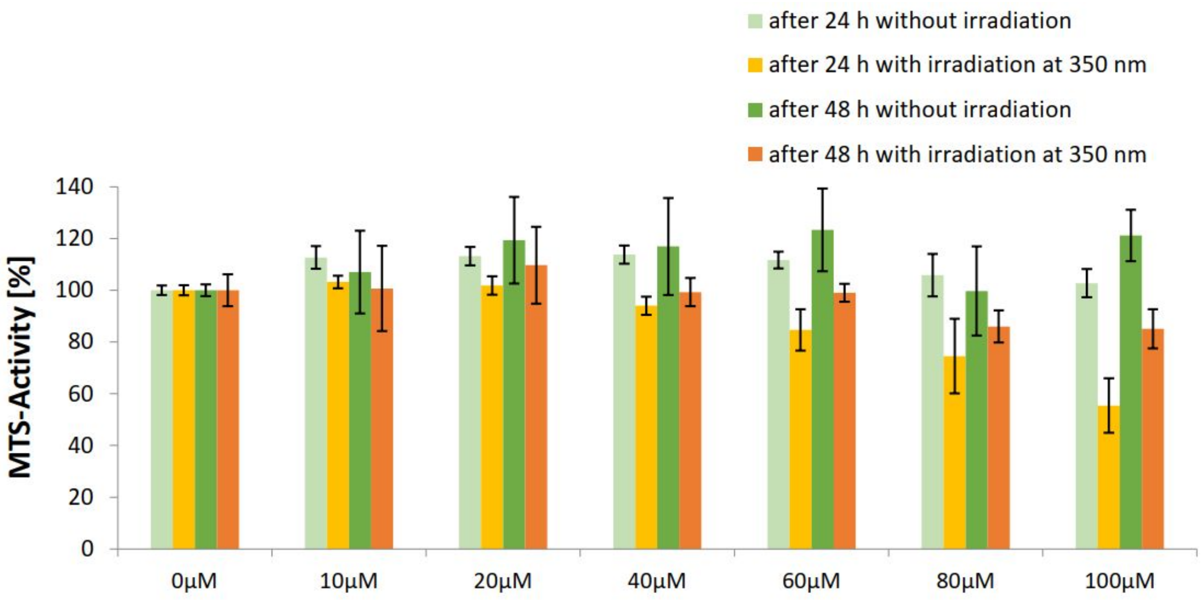

(a)

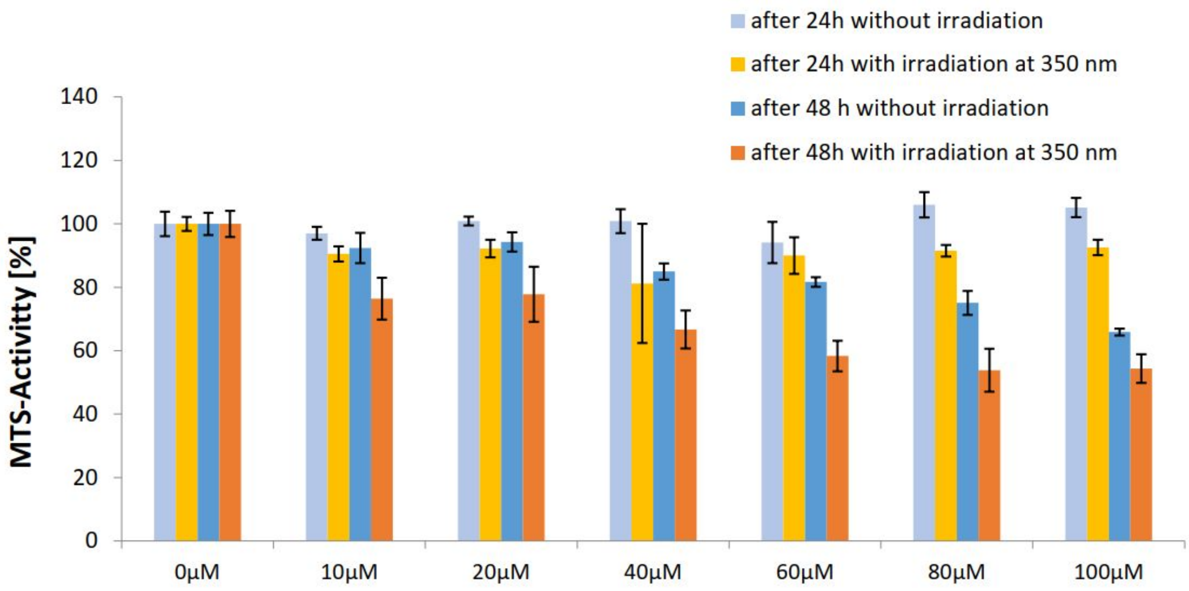

(b)

Figure S35. MTS assay of [**5**]CF_3SO_3 (0 - 100 µM) in A431 **(a)** and HEK293 **(b)** cell lines, measured after 24 and 48 h; with or without 10 min exposure to 350 nm radiation (E_v = 6 mW/cm^2) at 37 °C. The percentage of cell viability is expressed relative to untreated cells (ANOVA at α = 0.05).

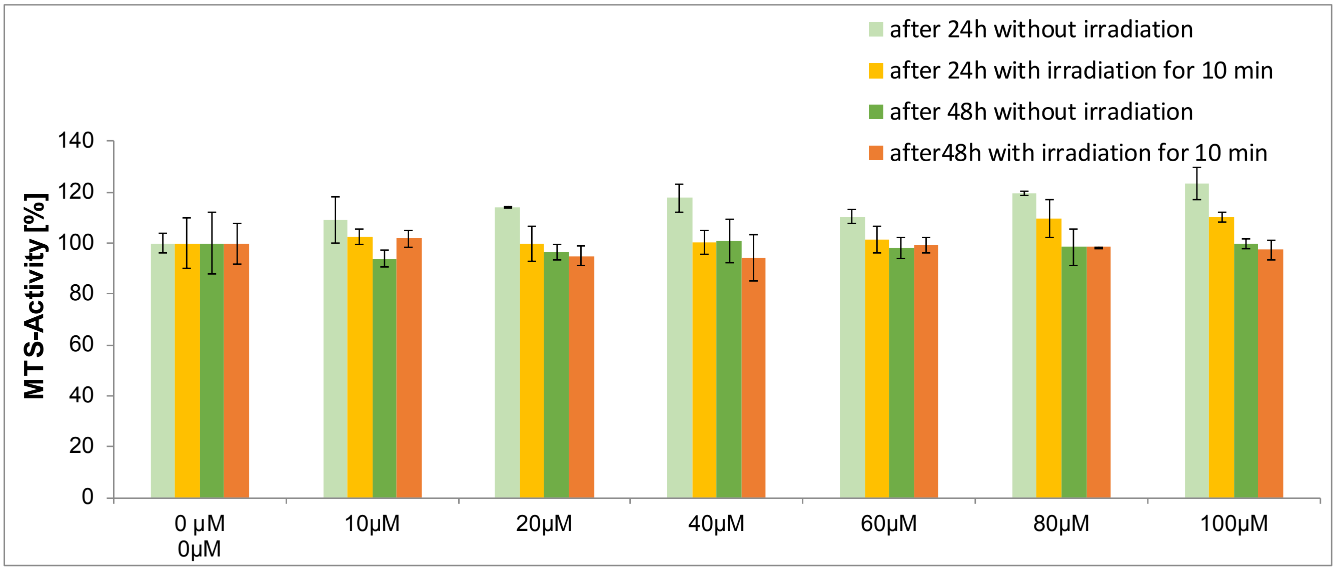

(a)

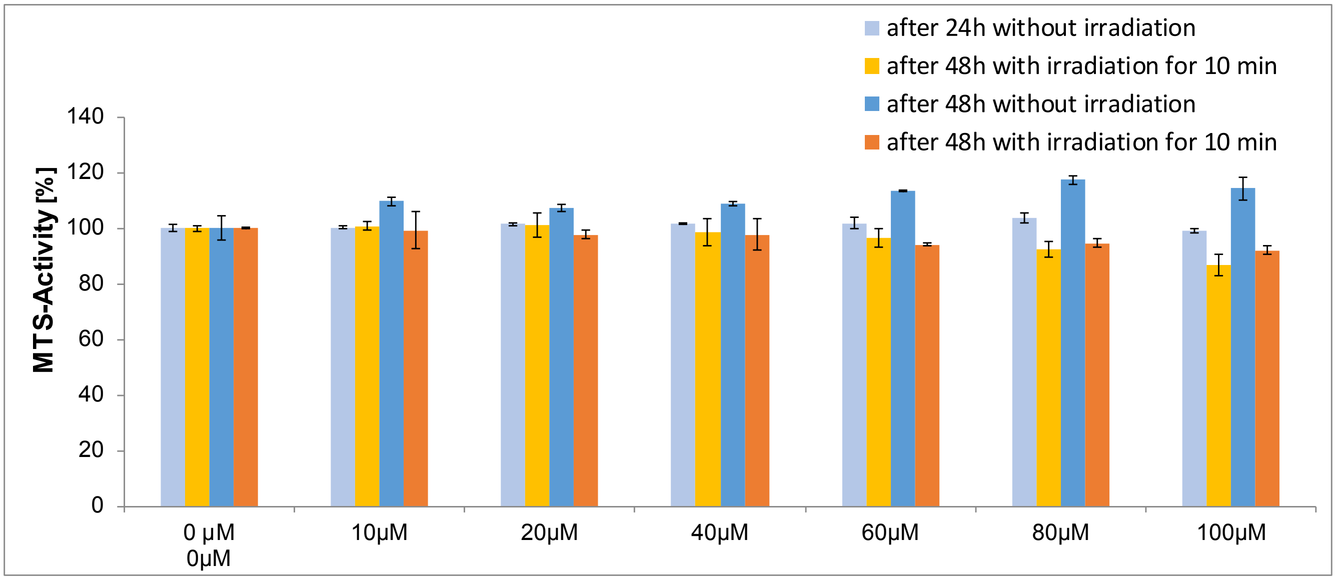

(b)

Figure S36. Fitted Hill slope of [**1**]CF_3SO_3 (0-100 µM) in A431 (*left*) and HEK293 (*right*) cell lines, kept in the dark (*top*) and exposed to 350 nm radiation (E_v = 6 mW/cm^2) at 37 °C for 10 min (*bottom*). MTS activity was measured after 48 h.

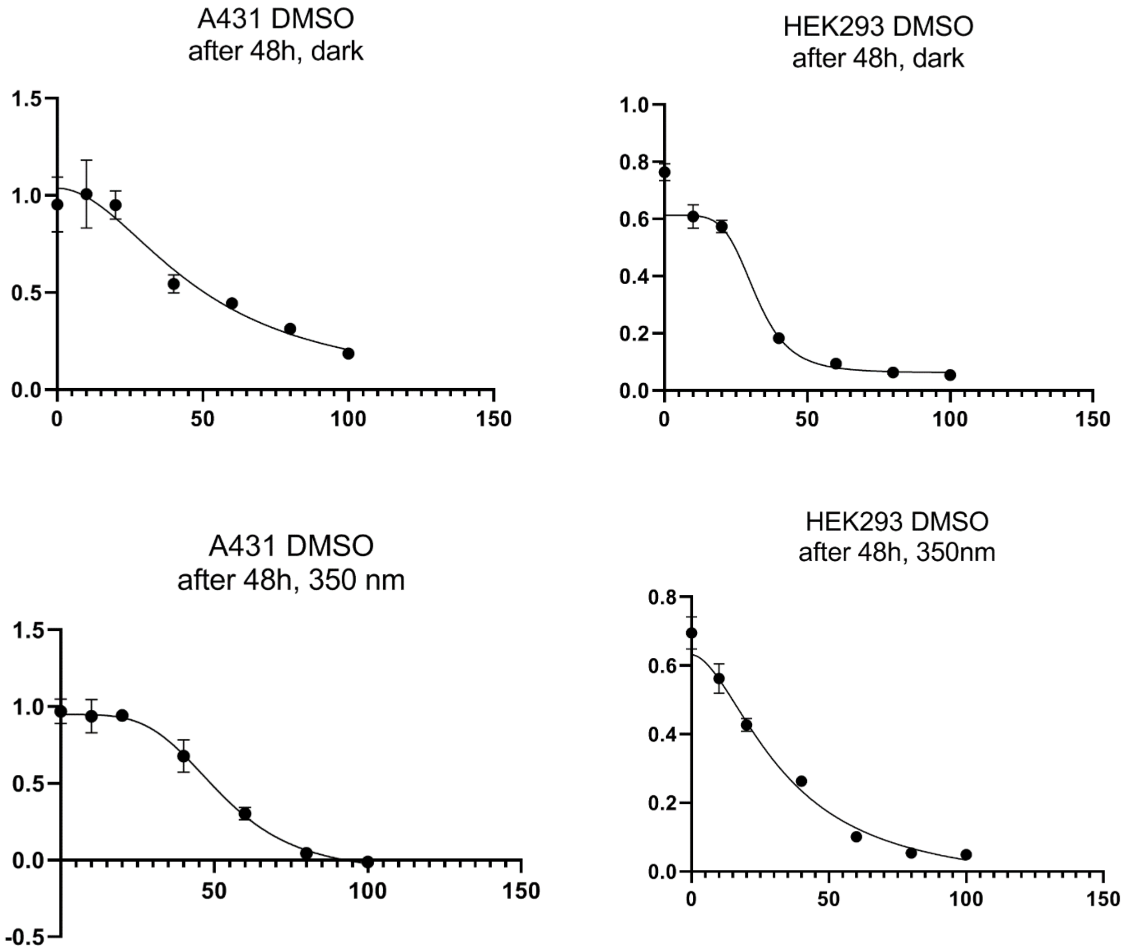

Figure S37. Fitted Hill slope of [**3**]NO_3 (0-125 µM) in A431 (*left*) and HEK293 (*right*) cell lines exposed to 350 nm radiation (E_v = 6 mW/cm^2) for 10 min at 37 °C. MTS activity measured after 48 h.

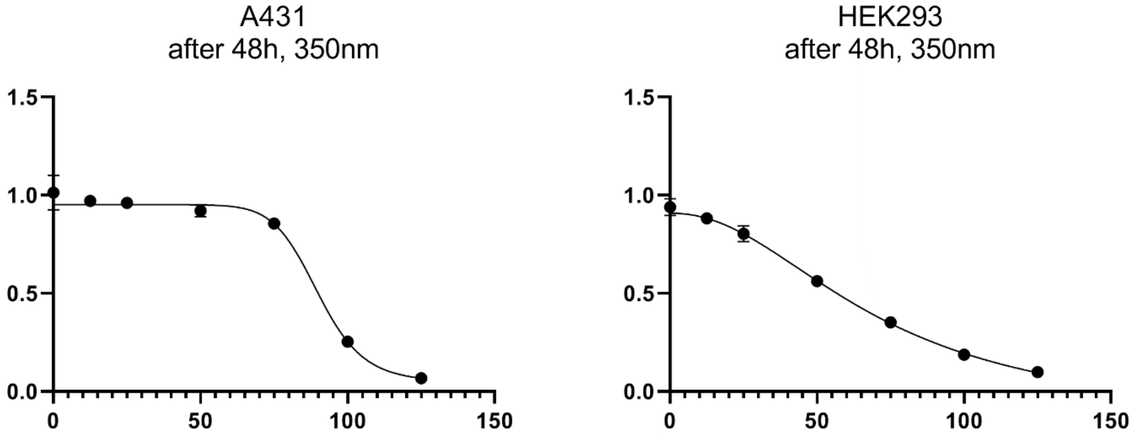

Supplement: Supplementary file 1 — ic2c00504_si_001.pdf [file ic2c00504_si_001.pdf]
